# SCOPE 2021: a new scorecard for osteoporosis in Europe

**DOI:** 10.1007/s11657-020-00871-9

**Published:** 2021-06-02

**Authors:** John A. Kanis, Nicholas Norton, Nicholas C. Harvey, Trolle Jacobson, Helena Johansson, Mattias Lorentzon, Eugene V. McCloskey, Carl Willers, Fredrik Borgström

**Affiliations:** 1 Mary McKillop Institute for Health Research, Australian Catholic University, Melbourne, Australia; 2 Centre for Metabolic Bone Diseases, University of Sheffield Medical School, University of Sheffield, Beech Hill Road, Sheffield, S10 2RX UK; 3 Quantify Research, Stockholm, Sweden; 4 MRC Lifecourse Epidemiology Unit, University of Southampton, Southampton, UK; 5 NIHR Southampton Biomedical Research Centre, University of Southampton and University Hospital Southampton NHS Foundation Trust, Southampton, UK; 6 Geriatric Medicine, Institute of Medicine, Sahlgrenska Academy, University of Gothenburg, Gothenburg, Sweden; 7 MRC Versus Arthritis Centre for Integrated Research in Musculoskeletal Ageing, Mellanby Centre for Bone Research, University of Sheffield, Sheffield, UK; 8 Department of Neurobiology, Care Sciences and Society, Karolinska Institutet, Stockholm, Sweden; 9 Department of Learning, Informatics, Management and Ethics (LIME), Karolinska Institutet, Stockholm, Sweden

**Keywords:** SCOPE, Scorecard, Osteoporosis, Epidemiology, Burden of disease, Cost, European Union, Treatment uptake, Treatment gap, Service provision, Service uptake, Policy framework

## Abstract

***Summary*:**

This scorecard summarises key indicators of the burden of osteoporosis and its management in the 27 member states of the European Union, as well as the UK and Switzerland. The resulting scorecard elements, assembled on a single sheet, provide a unique overview of osteoporosis in Europe.

**Introduction:**

The scorecard for osteoporosis in Europe (SCOPE) is a project of the International Osteoporosis Foundation (IOF) that seeks to raise awareness of osteoporosis care in Europe. The aim of this project was to develop a scorecard and background documents to draw attention to gaps and inequalities in the provision of primary and secondary prevention of fractures due to osteoporosis.

**Methods:**

The SCOPE panel reviewed the information available on osteoporosis and the resulting fractures for each of the 27 countries of the European Union plus the UK and Switzerland (termed EU27+2). The information obtained covered four domains: background information (e.g. the burden of osteoporosis and fractures), policy framework, service provision and service uptake, e.g. the proportion of men and women at high risk that do not receive treatment (the treatment gap).

**Results:**

There was a marked difference in fracture risk among the EU27+2 countries. Of concern was the marked heterogeneity in the policy framework, service provision and service uptake for osteoporotic fracture that bore little relation to the fracture burden. For example, despite the wide availability of treatments to prevent fractures, in the majority of the EU27+2, only a minority of patients at high risk receive treatment even after their first fracture. The elements of each domain in each country were scored and coded using a traffic light system (red, orange, green) and used to synthesise a scorecard. The resulting scorecard elements, assembled on a single sheet, provide a unique overview of osteoporosis in Europe.

**Conclusions:**

The scorecard enables healthcare professionals and policy makers to assess their country’s general approach to the disease and provide indicators to inform the future provision of healthcare.

## About SCOPE

The mission of the **s**core**c**ard for **o**steo**p**orosis in **E**urope (SCOPE) project is to raise awareness of osteoporosis care in Europe. SCOPE permits an in-depth comparison of the quality of care of osteoporosis across the 27 member states of the European Union (EU27), together with the UK and Switzerland (termed EU27+2).

Osteoporosis is a complex, chronic disease that can be treated and managed in a number of ways. Improvements in medication and diagnostic techniques in the past 30 years have provided highly effective ways to reduce the risk of osteoporotic fractures. In Europe, however, research has shown significant heterogeneity in the different national approaches to the management of the disease.

The scorecard summarises key indicators of the burden of osteoporosis and its management in each of the member states of the European Union to draw attention to the disparities in healthcare provision that can serve in the setting of benchmarks to inform patients, healthcare providers and policy makers in the EU. This update of the original SCOPE publication and scorecard compares the original results from 2010 to data as recent as 2019. The newer data provides a more recent overview, as well as a way to compare management of osteoporosis over time, within and between the EU27+2 countries.

In developing this scorecard, the aim is to stimulate a balanced, common and optimal approach to the management of osteoporosis throughout the EU27+2.

**Table of contents**

- Letter to all Europeans
- Introduction
- 1. Burden of disease
- 2. Policy framework
- 3. Service provision
- 4. Service uptake
- 5. Scorecard
- Acknowledgements
- Abbreviations and glossary

## A letter to all Europeans

The statistics are startling.

One in three women and at least one in six men will suffer an osteoporotic fracture in their lifetime. For every minute that passes eight new fracture cases arise in the EU. It is estimated that more than 23 million men and women are at high risk of osteoporotic fractures in the European Union.

Osteoporosis and the 4.3 million fragility fractures that it causes cost the health care systems of Europe in excess of €56 billion each year based on data for 2019. Only 3% of this money was spent on medical treatment. But numbers do not tell the full story. For the individuals who suffer fractures as a result of the disease, the stories are personal. Pain, disability, reduced mobility and long-term disability are all too frequent. Additionally, some fractures related to osteoporosis result in death. Nearly a quarter of a million deaths occur each year in Europe as a direct consequence of hip or spine fractures.

SCOPE is committed to helping individuals reduce their risk of osteoporosis and to ensuring that all Europeans have access to the best diagnosis and treatment. Components that are critical to achieving this goal include government policy, access to risk assessments, and access to medications. This update of the scorecard allows Europeans to measure how well their country is able to access these elements through publicly funded health care systems. It also provides a new benchmark to follow trends in osteoporosis management, and to measure future progress.

Our research reveals that facilities and access to testing for osteoporosis is far from adequate. Access to drug treatment that can help prevent fractures varies markedly from country to country; in some member states, individuals with osteoporosis are restricted from accessing effective treatment options. Less than half of women at high risk of fracture are treated despite the high cost of fractures and the availability of affordable medications.

Action is required. The national osteoporosis societies within the International Osteoporosis Foundation are calling for a Europe-wide strategy and parallel national strategies to provide coordinated osteoporosis care and to reduce debilitating fractures and their impact on individual lives and the health care system. We welcome the opportunity to partner with governments at the national and European level to develop and implement these strategies. Together we can improve bone health for all in Europe.


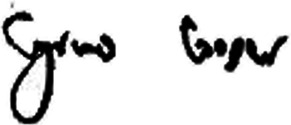
Cyrus Cooper

President of IOF


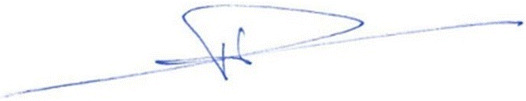
Philippe Halbout

CEO of IOF


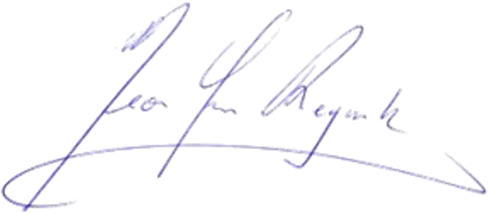
Jean-Yves Reginster

President of the IOF Committee of National Societies


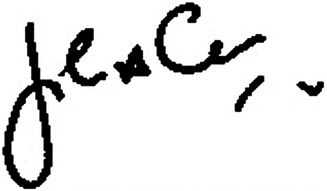
John A Kanis

Chair of SCOPE

## Introduction

### The basis for SCOPE

SCOPE 2021 comprises a compendium of information available on the burden of osteoporosis and healthcare provision and uptake in the EU 27+2. Its history begins over 20 years ago with a series of regional audits of the International Osteoporosis Foundation (IOF) [1–3]. The information base has been supplemented with reports undertaken through the IOF on the burden of osteoporosis in the whole or selected countries of the EU, as well as the UK and Switzerland [4–7]. This information base was broadened and updated by IOF to inform the SCOPE panel members through its outreach to over 40 national osteoporosis societies throughout Europe by means of a structured questionnaire that was sent to all IOF national societies and key opinion leaders in each country.

From the information available, the SCOPE panel developed indicators of osteoporosis that could be applied to each member state under four broad domains:

- Burden of disease—including the burden of osteoporosis, fractures and forecasts for the future.
- Policy framework—such as the availability of public health programmes.
- Service provision—including assessment and treatments of osteoporosis.
- Service uptake—e.g. the proportion of men and women at high risk that do not receive treatment (the treatment gap).

For each domain, a synthesis was summarised, and tabular information provided for each member state which appears in the body of the report. For key indicators, termed scorecard elements, the information was scored and the basis for the score allocation provided. For example, the remaining lifetime risk of a hip fracture at the age of 50 years ranged from 7.0% (Romania) to 25.1% (Sweden) in women from the different countries of the EU. Countries were categorised by tertile of fracture risk. High risk countries were colour coded red, intermediate risk coded orange and low risk countries coded green. A similar ‘traffic light’ approach was applied to each element in each domain. The resulting scorecard elements were then assembled on a single sheet to provide a unique overview of osteoporosis in Europe. It will enable healthcare professionals and policy makers to assess their country’s general approach to the disease and provide indicators to inform future provision of healthcare.

The first scorecard, published in 2013, reviewed the state of osteoporosis in 2010 [8]. The present scorecard not only updates this to 2019 but, because of the use of consistent methodology also permits some comparisons over time.

Some caveats are appropriate in the interpretation of scores. Green is not necessarily ‘good’, and red is not necessarily ‘bad’. An example of the former is the treatment gap (the proportion of high-risk patients given specific treatment for osteoporosis). Whereas countries coded green have a treatment gap of 30-60%, treatment gaps of less than 20% might be an appropriate target. Coding all countries red would, however, not permit the comparative performance of one country against another. Other examples are highlighted in the text.

In the development of a scorecard, a first step is to define what is to be measured. There is of course not one element that captures all the functional aspects of health care. That the cost of fractures in 2019 is €5.5 billion in the UK and €9.4 billion in Italy says little except one country spends more than the other. No single metric can suffice. Various integrated and multidimensional performance measurement systems have been developed [9, 10] that are suited to examine complex organisations. A problem in international comparison is the difficulty of comparing like with like when the methods of data capture differ. This in turn limits the scorecard to the art of the possible (that which can be measured) rather than the art of the ideal (that one would wish to measure)

### Osteoporosis

Osteoporosis is characterized by reduced bone mass and disruption of bone architecture, resulting in increased bone fragility and increased fracture risk [11]. The publication of a World Health Organization (WHO) report on the assessment of fracture risk and its application to screening for postmenopausal osteoporosis in 1994 provided diagnostic criteria for osteoporosis based on the measurement of bone mineral density (BMD) and recognized osteoporosis as an established and well-defined disease that affected more than 75 million people in the US, Europe and Japan [12].

BMD is most often given as a *T* score that describes the number of SDs by which the BMD in an individual differs from the mean value expected in young healthy women. The operational definition of osteoporosis is defined as a value for BMD 2.5 SD or more below the young female adult mean (*T* score less than or equal to –2.5 SD) [13]. BMD at the femoral neck is the international reference standard [14]. The consequences of low BMD reside in the fractures that arise. The relationship between BMD and fracture is continuous in that the lower the BMD, the higher the fracture risk [15].

### Osteoporotic fractures

The definition of an osteoporotic fracture is not straightforward and the terms osteoporosis, fragility fracture and osteoporotic fractures have inherent ambiguities. An approach adopted widely is to consider low-energy fractures as being osteoporotic. This has the merit of recognizing the multifactorial causation of fracture. However, with high-energy trauma, osteoporotic individuals are more likely to fracture than those without osteoporosis [16]. There is also a disparity between low-energy fractures and fractures associated with reductions in BMD [17, 18]. The classification is therefore incomplete. An alternative approach is to designate an osteoporotic fracture as one sustained in an individual with osteoporosis as defined by the *T* score and World Health Organization criteria but this has inherent conceptual and practical difficulties [19]. Thus, a minority of fragility fractures occur in individuals with a BMD *T* score of less than − 2.5 SD [17, 18]. The approach we have taken is to identify sites of fracture that increase in frequency the lower the BMD and the incidence of which increase progressively with age after the age of 50 years [20]. The most common fractures associated with osteoporosis defined in this way are those at the hip, spine, forearm and humerus but many other fractures after the age of 50 years are associated with low BMD and should be regarded as osteoporotic [18]. These include fractures of the ribs, tibia, pelvis and other femoral fractures.

For the purpose of this report, the term osteoporosis is used in a generic sense rather than a specific sense unless otherwise specified. For example, the ‘cost of osteoporosis’ refers to the cost of fractures at sites associated with osteoporosis irrespective of the *T* score.

The incidence of fragility fractures increases markedly with age, though the rate of rise with age differs for different fracture outcomes. For this reason, the proportion of fractures at any site also varies with age. For example, forearm fractures account for a greater proportion at younger ages than in the elderly. Conversely, hip fractures are rare at the age of 50 years but become the predominant osteoporosis fracture from the age of 75 years. In women, the median age for distal forearm fractures is around 65 years and for hip fracture, 80 years. Thus, both the number of fractures and the type of fracture are critically dependent on the age of the populations at risk.

Hip fracture is the most serious osteoporotic fracture. Hip fracture is painful and nearly always necessitates hospitalization and surgical intervention. Up to 20% of patients die in the first year following hip fracture, mostly as a result of serious underlying medical conditions [21] and less than half of survivors regain the level of function that they had prior to the hip fracture [22]. Thus, not all deaths associated with hip fracture are due to the hip fracture event and it is estimated that approximately 30% of deaths are causally related. When this is taken into account, hip fracture causes more deaths in Sweden than road traffic accidents and about the same number as those caused by breast cancer [23]. An updated comparison, presented in *Chapter 1e*, indicates that fragility fractures in Sweden account for more deaths than many chronic noncommunicable diseases including cerebrovascular disease, lung cancer, chronic lower respiratory disease and diabetes.

#### References

1. Foundation IO (1998) Report on osteoporosis in the European Community: A call to action. Action for prevention. Nyon, Switzerland https://www.iofbonehealth.org/sites/default/files/PDFs/EU%20Reports/eu_report_1998.pdf. Accessed 23 July 2020
2. Foundation IO (2001) Osteoporosis in the European Community: A call to action. An audit of policy developments since 1998. Nyon, Switzerland https://www.iofbonehealth.org/osteoporosis-european-community-call-action. Accessed 23 July 2020
3. Foundation IO (2010) Osteoporosis in the European Union in 2008. Ten years of progress and ongoing challenges. Nyon Switzerland https://www.iofbonehealth.org/osteoporosis-european-union-ten-years-progress-and-ongoing-challenges. Accessed 23 July 2020
4. Borgstrom F, Karlsson L, Ortsater G, Norton N, Halbout P, Cooper C, Lorentzon M, McCloskey EV, Harvey NC, Javaid MK, Kanis JA (2020) Fragility fractures in Europe: burden, management and opportunities. Arch Osteoporos 15:59
5. Hernlund E, Svedbom A, Ivergard M, Compston J, Cooper C, Stenmark J, McCloskey EV, Jonsson B, Kanis JA (2013) Osteoporosis in the European Union: medical management, epidemiology and economic burden. A report prepared in collaboration with the International Osteoporosis Foundation (IOF) and the European Federation of Pharmaceutical Industry Associations (EFPIA). Arch Osteoporos 8:136
6. Strom O, Borgstrom F, Kanis JA, Compston J, Cooper C, McCloskey EV, Jonsson B (2011) Osteoporosis: burden, health care provision and opportunities in the EU: a report prepared in collaboration with the International Osteoporosis Foundation (IOF) and the European Federation of Pharmaceutical Industry Associations (EFPIA). Arch Osteoporos 6:59–155
7. Svedbom A, Hernlund E, Ivergard M, Compston J, Cooper C, Stenmark J, McCloskey EV, Jonsson B, Kanis JA (2013) Osteoporosis in the European Union: a compendium of countryspecific reports. Arch Osteoporos 8:137
8. Kanis JA, Borgstrom F, Compston J, Dreinhofer K, Nolte E, Jonsson L, Lems WF, McCloskey EV, Rizzoli R, Stenmark J (2013) SCOPE: a scorecard for osteoporosis in Europe. Arch Osteoporos 8:144
9. Ghalayini Alaa M, Noble James S (1996) The changing basis of performance measurement. International Journal of Operations & Production Management 16:63–80
10. Wongrassamee S, Gardiner P, Simmons J (2003) Performance Measurement Tools: The Balanced Scorecard and the EFQM Excellence Model. Measuring Business Excellence:7
11. Anonymous (1993) Consensus development conference: diagnosis, prophylaxis, and treatment of osteoporosis. Am J Med 94:646–650
12. World Health Organization (1994) Assessment of fracture risk and its application to screening for postmenopausal osteoporosis. Report of a WHO Study Group, World Health Organ Tech Rep Ser
13. Kanis JA, Melton LJ 3rd, Christiansen C, Johnston CC, Khaltaev N (1994) The diagnosis of osteoporosis. J Bone Miner Res 9:1137–1141
14. Kanis JA, McCloskey EV, Johansson H, Oden A, Melton LJ 3rd, Khaltaev N (2008) A reference standard for the description of osteoporosis. Bone 42:467–475
15. Johnell O, Kanis JA, Oden A, Johansson H, De Laet C, Delmas P, Eisman JA, Fujiwara S, Kroger H, Mellstrom D, Meunier PJ, Melton LJ 3rd, O'Neill T, Pols H, Reeve J, Silman A, Tenenhouse A (2005) Predictive value of BMD for hip and other fractures. J Bone Miner Res 20:1185–1194
16. Sanders KM, Pasco JA, Ugoni AM, Nicholson GC, Seeman E, Martin TJ, Skoric B, Panahi S, Kotowicz MA (1998) The exclusion of high trauma fractures may underestimate the prevalence of bone fragility fractures in the community: the Geelong Osteoporosis Study. J Bone Miner Res 13:1337–1342
17. Leslie WD, Schousboe JT, Morin SN, Martineau P, Lix LM, Johansson H, McCloskey EV, Harvey NC, Kanis JA (2020) Fracture risk following high-trauma versus low-trauma fracture: a registry-based cohort study. Osteoporos Int 31:1059–1067
18. Seeley DG, Browner WS, Nevitt MC, Genant HK, Scott JC, Cummings SR (1991) Which fractures are associated with low appendicular bone mass in elderly women? The Study of Osteoporotic Fractures Research Group. Ann Intern Med 115:837–842
19. Kanis JA, McCloskey EV, Harvey NC, Johansson H, Leslie WD (2015) Intervention Thresholds and the Diagnosis of Osteoporosis. J Bone Miner Res 30:1747–1753
20. Kanis JA, Oden A, Johnell O, Jonsson B, de Laet C, Dawson A (2001) The burden of osteoporotic fractures: a method for setting intervention thresholds. Osteoporos Int 12:417–427
21. Keene GS, Parker MJ, Pryor GA (1993) Mortality and morbidity after hip fractures. Bmj 307:1248–1250
22. Melton LJ 3rd (2003) Adverse outcomes of osteoporotic fractures in the general population. J Bone Miner Res 18:1139–1141
23. Kanis JA, Oden A, Johnell O, De Laet C, Jonsson B, Oglesby AK (2003) The components of excess mortality after hip fracture. Bone 32:468–473

## Chapter 1: Burden of disease

### 1a—Healthcare cost of osteoporotic fractures

#### Domain

Burden of disease—background information

#### Background and aims

Cost of illness studies can take a societal perspective (including all cost incurred directly or indirectly by society) or a payer perspective (usually includes all costs carried by the healthcare and social system). Both play an important role in the understanding of disease management and may aid decisions concerning societal resource allocation for research, development, and funding of new treatments. Results from cost of illness studies can also be utilised to monitor medical progress.

The main objective of this section is to provide detail on the current cost of osteoporotic fractures in the countries of the European Union.

#### Methods

The cost of osteoporotic fractures was updated using the same methodological approach as used in the previous SCOPE study [24, 25]. The fracture costs were first determined without intangible costs (i.e. the monetary value of Quality-Adjusted Life-Years (QALYs) lost due to death and disability) [24]. Costs of fracture-related productivity losses were not included.

This type of cost mainly occurs in the working population. The median age of retirement in Europe is 65 years [26]. Estimates on fracture related productivity costs are very scarce in the literature. Therefore, it is difficult to impute values for all fracture types for all countries in the analysis. Most fractures occur in elderly retired patients. However, in Sweden, about 20% of fractures occur in pre-retirement ages [27]. In six European countries participating in the ICUROS study, the average number of days off work in the preretirement population was 103 days/1000/year, of which 57% was due to hip fracture, 27% from vertebral fracture and 16% from other fractures [28]. The cost of osteoporotic fracture is therefore somewhat underestimated in this analysis.

Fractures were categorised by site, comprising hip, vertebral, distal forearm, and other osteoporotic fractures. Other fractures consisted of humerus, ribs, tibia, pelvis, and other femoral fractures. Since the previous SCOPE study, empirical but incomplete fracture cost estimates were either updated or added for Belgium, Estonia, France, Greece, Ireland, Italy, Netherlands, Portugal, Slovenia, Spain, Switzerland and the UK. For countries where fracture costs were unavailable for the previous SCOPE study or for this study, costs were imputed from the nearest country available by adjusting for differences in healthcare price levels between the relevant countries. In this case, no intervention cost estimates were available for Malta and Cyprus, so the cost per capita in Italy and Greece, respectively, was used as proxies to estimate the intervention costs. All costs were adjusted for inflation to match the price level of year 2019. Swiss estimates for the total cost in 2010 were added separately, as these were not available in the original SCOPE study [29].

Costs were divided into the cost of incident fractures in 2019 (i.e. those costs that were incurred the first year after fracture), the ongoing cost in 2019 of fractures occurring before 2019 (long-term disability), and the cost of intervention for osteoporosis. Hip fracture costs in the second and following years after the event related to institutionalisation were based on the proportion of patients that became dependent in the long-term. Due to lack of information, institutionalisation costs were only ascribed to hip fractures. Long-term medical care costs related to hip and vertebral fracture were imputed, based on a UK estimate [30]. It was conservatively assumed that ‘other fractures’ did not incur any longer-term costs after the first year. Because this provides an underestimate of the actual cost of fracture an additional analysis, not included in the first SCOPE-study, was conducted. By assuming that the morbidity loss of fractures is proportional to the fracture costs, it is possible to impute the missing cost elements for non-hip fractures using hip fracture morbidity equivalents [31]. More details on this approach are described in Kanis et al [31].

The health burden of fragility fractures was additionally measured in terms of QALYs lost. The QALY is a multidimensional outcome measure that incorporates both the quality (health related) and quantity (length) of life. The value of a QALY was set at value of 2× GDP per capita [32].

#### Results

The direct cost of incident fractures in the EU27+2 in 2019 was €36.3 billion (Table 1). Added to this was the ongoing cost in 2019 resulting from fractures that occurred before 2019, which amounted to €19.0 billion (long-term disability). The cost of pharmacological intervention (assessment and treatment) was €1.6 billion. Thus, the total direct cost in the EU27+2 (excluding the value of QALYs lost) amounted to €56.9 billion in 2019. First year, subsequent year, and pharmacological costs accounted for 64%, 33% and 3% of the costs, respectively. In 2010, the total direct cost in the EU27 (excluding the value of QALYs lost) amounted to €37.4 billion. First year costs (€24.6 billion), subsequent year costs (€10.7 billion) and pharmacological costs (€2.1 billion) accounted for 66, 29 and 5% of the 2010 total, respectively. Thus, over a nine-year period, direct costs have increased by 64% without a commensurate increment in pharmacological costs.

**Table 1:** Cost of osteoporosis in the EU27+2 in 2019 (€ million, 2019)

Country	Incident fracture costs	Long-term disability costs	Intervention costs	Total costs	Cost per capita (€) 2010	Cost per capita (€) 2019	QALYs lost (€m) 2010	QALYs lost (€m) 2019
Austria	833.52	468.10	41.70	1343.32	104.84	151.84	2100	4111
Belgium	766.36	321.85	33.97	1122.18	62.91	98.25	1914	3079
Bulgaria	135.09	41.30	9.19	185.58	6.62	26.42	130	327
Croatia	71.30	58.55	6.08	135.93	-	31.75	-	373
Cyprus	64.09	12.71	8.92	85.73	51.87	72.08	86	95
Czech Republic	260.88	121.34	14.05	396.27	28.69	37.29	695	1350
Denmark	852.75	548.37	51.15	1452.27	209.68	250.50	1881	3096
Estonia	18.05	11.89	1.68	31.62	24.28	23.94	65	106
Finland	406.60	190.90	13.62	611.12	78.36	110.75	915	1423
France	5047.97	1769.89	162.22	6980.07	84.98	104.20	9170	12001
Germany	10235.08	3345.62	249.36	13830.06	121.40	166.77	16473	28232
Greece	694.70	203.51	80.46	978.68	66.22	91.23	1394	1518
Hungary	348.93	79.65	20.85	449.44	22.07	46.01	512	890
Ireland	290.84	135.72	37.73	464.29	55.18	95.66	470	1456
Italy	5438.79	3749.16	258.61	9446.55	129.12	156.32	9680	14980
Latvia	28.04	18.75	1.84	48.63	18.76	25.24	79	170
Lithuania	53.14	35.08	2.79	91.01	15.45	32.63	89	258
Luxembourg	28.26	10.78	1.58	40.62	47.45	66.84	163	317
Malta	18.59	8.41	2.07	29.06	45.25	60.10	26	65
Netherlands	652.72	708.35	42.82	1403.88	55.18	81.47	2056	3735
Poland	332.89	347.32	13.52	693.73	17.66	18.27	1094	2172
Portugal	523.86	464.82	14.82	1003.51	59.59	97.60	640	720
Romania	91.02	150.13	16.17	257.32	6.62	13.21	374	1035
Slovakia	135.24	41.73	16.68	193.66	22.07	35.55	312	724
Slovenia	60.81	26.74	8.15	95.69	30.90	46.29	185	302
Spain	1813.37	2197.98	302.95	4314.30	69.53	92.34	3610	6224
Sweden	1440.28	848.47	44.63	2333.37	176.58	229.14	2942	4457
Switzerland	2624.76	745.65	59.91	3430.32	190.22	402.78	3242	5166
UK	3031.07	2339.81	111.21	5482.09	96.01	82.45	9599	14465
EU27+2	36298.99	19002.57	1628.74	56930.30	85.80*	109.12	69898*	112850

*The EU27+2 estimates for cost per capita in 2010 and QALYs lost in 2010 do not include values for Croatia, as the data were not available

Whilst the proportion of pharmacological intervention costs to total costs in 2019 was low on average, some intercountry variation was observed: the lowest proportion of costs attributable to intervention was observed in Portugal (1.5%) and the highest costs in Cyprus (10.4%). Hip fractures were estimated to account for 57% of the total costs, vertebral fractures for 10%, distal forearm fractures for 2% and other for 32%.

**Fig. 1:**
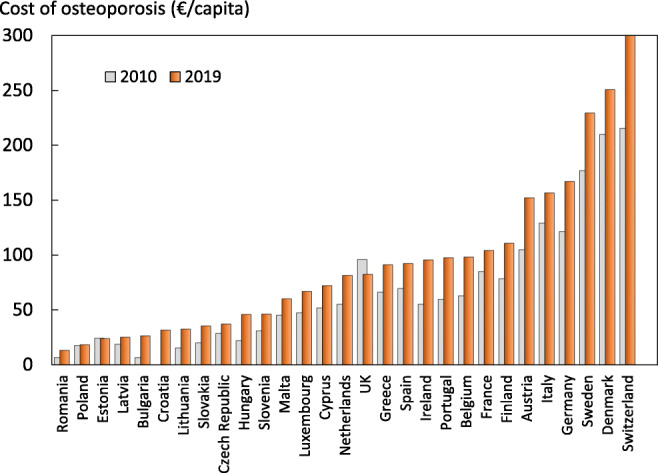
Cost of fragility fractures expressed as cost/capita in 2019 and 2010. The 2010 values are shown as grey bars

In 2019, the average direct cost of osteoporotic fractures was €109.12 for each individual in the EU27+2, while in 2010 the average for the EU27 was €85.77 (after adjusting for inflation). There was a large variation in the ‘osteoporosis tax’ (cost per capita) which was highest in Switzerland (€403/person) and Denmark (€251), and lowest in Romania (€13) and Poland (€18). Changes in the osteoporosis tax since 2010 are given in Fig. 1. In 2019 the osteoporosis tax had increased in all countries except for the UK and Estonia.

**Fig. 2:**
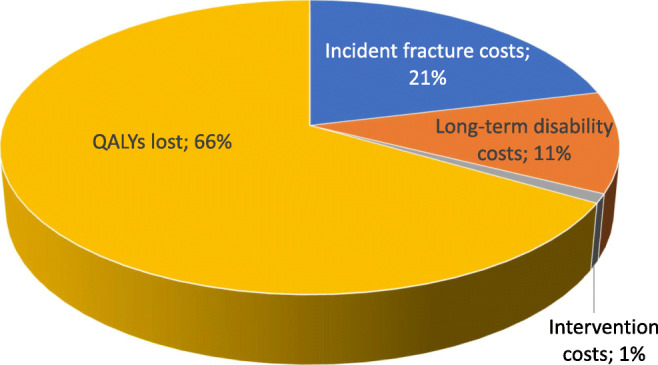
Components (%) of the cost of osteoporosis and fractures

The cost of QALYs lost in the EU27+2 was substantial, amounting to €112.9 billion and giving a direct and indirect cost total (including QALYs) of €169.8 billion in 2019. Intervention costs amounted to about 1% of the total direct and indirect costs (Fig. 2) and 3% of the direct costs.

Compared with 2010, the cost per capita (after adjusting 2010 costs for inflation) has increased most in percentage terms in Bulgaria (+299%) and Lithuania (+111%). The percentage change was lowest in the UK (−14%) and Estonia (−1%). QALY loss has increased by percentage the most in Ireland (+ 210%) and Romania (+177%), whilst Greece (+9%) and Cyprus (10%) had had the smallest increases in lost QALYs. The large changes in cost per capita for Bulgaria [33], Estonia [34] and Ireland [35] can be explained by updates in the estimates for fracture incidence and/or fracture related costs. The small change in the UK can be explained by an update in the fracture cost inputs [36, 37].

**Fig. 3:**
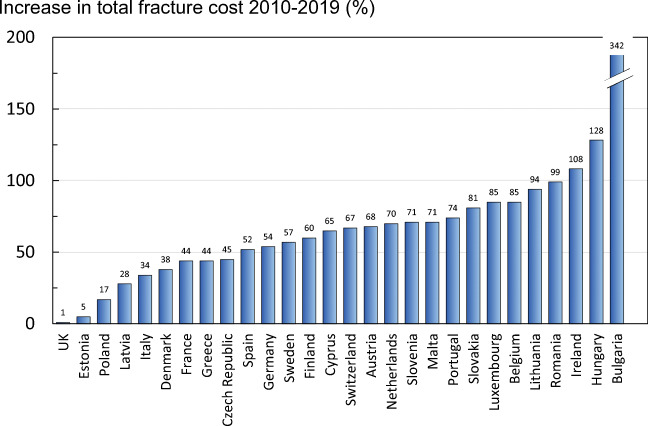
Increase in total fracture cost (%) in 2019 compared with 2010 values

The percentage increase in total cost for 2019 is presented in Fig. 3. As shown in Table 1, the countries with the largest absolute increase in total cost were Germany (+€4.8 billion), Italy (+€2.4 billion) and France (+€2.1 billion). This absolute increase was mainly associated with the larger populations in these countries. Bulgaria (+342%) and Hungary (+128%) have seen the largest percentage increase in total costs, while the UK (+1%) and Estonia (+5%) have seen the smallest increase. When using morbidity equivalents to also account for the long-term cost of non-hip fractures, the total direct cost for the UE27+2 increases by 20%, to 68.4 billion.

#### Comment

There are few directly comparable studies in other noncommunicable diseases. The European Heart Network has estimated that for cardiovascular disease, healthcare costs, productivity losses, and informal care comprised 53, 26 and 21%, respectively. Costs for pharmacological treatment accounted for 25% of the healthcare expenditure, substantially higher than that for osteoporosis. [38] For cerebrovascular disease, it has been estimated that healthcare costs, productivity losses, and informal care comprised 61, 18 and 21%, respectively. The cost for pharmacological treatment accounted for 3% of the total cost for cerebrovascular disease, which is comparable to that for osteoporosis [39].

#### References

24. Hernlund E, Svedbom A, Ivergard M, Compston J, Cooper C, Stenmark J, McCloskey EV, Jonsson B, Kanis JA (2013) Osteoporosis in the European Union: medical management, epidemiology and economic burden. A report prepared in collaboration with the International Osteoporosis Foundation (IOF) and the European Federation of Pharmaceutical Industry Associations (EFPIA). Arch Osteoporos 8:136
25. Kanis JA, Borgstrom F, Compston J, Dreinhofer K, Nolte E, Jonsson L, Lems WF, McCloskey EV, Rizzoli R, Stenmark J (2013) SCOPE: a scorecard for osteoporosis in Europe. Arch Osteoporos 8:144
26. Finnish Centre for Pensions (ETK) (2019) Retirement ages in different countries. Accessed 2020-03-31 2020 https://www.etk.fi/en/the-pension-system/international-comparison/retirementages/
27. Kanis JA, Johnell O, Oden A, Sembo I, Redlund-Johnell I, Dawson A, De Laet C, Jonsson B (2000) Long-term risk of osteoporotic fracture in Malmö. Osteoporos Int 11:669–674
28. Borgstrom F, Karlsson L, Ortsater G, Norton N, Halbout P, Cooper C, Lorentzon M, McCloskey EV, Harvey NC, Javaid MK, Kanis JA (2020) Fragility fractures in Europe: burden, management and opportunities. Arch Osteoporos 15:59
29. Svedbom A, IvergardM, Hernlund E, Rizzoli R, Kanis JA (2014) Epidemiology and economic burden of osteoporosis in Switzerland. Arch Osteoporos 9:187
30. Gutierrez L, Roskell N, Castellsague J, Beard S, Rycroft C, Abeysinghe S, Shannon P, Gitlin M, Robbins S (2012) Clinical burden and incremental cost of fractures in postmenopausal women in the United Kingdom. Bone 51:324–331
31. Kanis JA, Oden A, Johnell O, Jonsson B, de Laet C, Dawson A (2001) The burden of osteoporotic fractures: a method for setting intervention thresholds. Osteoporos Int 12:417–427
32. Borgstrom F, Johnell O, Kanis JA, Jonsson B, Rehnberg C (2006) At what hip fracture risk is it cost-effective to treat? International intervention thresholds for the treatment of osteoporosis. Osteoporos Int 17:1459–1471
33. Kirilova E, Johansson H, Kirilov N, Vladeva S, Petranova T, Kolarov Z, Liu E, Lorentzon M, Vandenput L, Harvey NC, McCloskey E, Kanis JA (2020) Epidemiology of hip fractures in Bulgaria: development of a country-specific FRAX model. Arch Osteoporos 15:28
34 Jürisson M, Pisarev H, Kanis J, Borgström F, Svedbom A, Kallikorm R, Lember M, Uusküla A (2016) Quality of life, resource use, and costs related to hip fracture in Estonia. Osteoporos Int 27:2555–2566
35. Hiligsmann M, McGowan B, Bennett K, Barry M, Reginster JY (2012) The clinical and economic burden of poor adherence and persistencewith osteoporosismedications in Ireland. ValueHealth 15:604–612
36. Gutiérrez L, Roskell N, Castellsague J, Beard S, Rycroft C, Abeysinghe S, Shannon P, Robbins S, Gitlin M (2011) Study of the incremental cost and clinical burden of hip fractures in postmenopausal women in the United Kingdom. J Med Econ 14:99–107

### 1b – Economic framework

#### Domain

Burden of disease—background information

#### Background and aims

Cost of illness studies provides no direct guidance on how resources should be allocated but may provide relevant information concerning the consequences of a disease in order to inform policy. Such data may aid decisions concerning societal resource allocation for research, development, and funding of new treatments. Results from cost-of-illness studies can also be used to assess the long-term consequences and value of medical progress.

The objective of this background section is to estimate the current cost of osteoporotic fractures in 27 EU countries, as well as Switzerland and the UK (hereafter referred to as the EU27+2), set against the wealth of the nation and the portion of that wealth allocated to healthcare. A more detailed consideration of the cost is given in Chapter 1a.

#### Methods

Direct costs of fractures in men and women from the EU27+2 aged 50 years or more were expressed as a proportion of total health care spending in the respective country [40], and as cost per capita of the general population [40–43].

#### Results

Health care spending varied markedly between countries, ranging from €1.3 billion in Cyprus to €371.4 billion in Germany (Table 2). The total spent on healthcare in the EU27+2 amounted to €1.6 trillion, with the cost of osteoporotic fractures representing approximately 3.5% of healthcare spending (i.e. €55.3 billion in 2019). This demonstrates a very substantial impact of fragility fractures on the present healthcare budgets of the EU countries.

**Table 2:** Cost of osteoporotic fractures in relation to the population and health care spending (2019)

Country	Population (thousands)	Health care spending (millions €)	Healthcare spending (% GDP)	Healthcare spending (€ per capita)	Fracture cost (% health care spending)
Austria	8,847	38,746.40	10.4	4,379.59	3.4
Belgium	11,422	45,746.49	10.3	4,005.10	2.4
Bulgaria	7,024	4,214.84	8.1	600.04	4.2
Croatia	4,089	3,350.71	6.8	819.36	3.9
Cyprus	1,189	1,323.06	6.7	1,112.50	5.8
Czech Republic	10,626	13,959.61	7.2	1,313.76	2.7
Denmark	5,797	29,811.80	10.1	5,142.23	4.7
Estonia	1,321	1,529.83	6.4	1,158.18	2.0
Finland	5,518	20,768.83	9.2	3,763.80	2.9
France	66,987	261,591.87	11.3	3,905.10	2.6
Germany	82,928	371,370.83	11.2	4,478.24	3.7
Greece	10,728	14,601.06	8.0	1,361.07	6.2
Hungary	9,769	8,597.02	6.9	880.05	5.0
Ireland	4,854	21,289.77	7.2	4,386.47	2.0
Italy	60,431	153,854.16	8.8	2,545.94	6.0
Latvia	1,927	1,621.85	5.3	841.85	2.9
Lithuania	2,790	2,745.30	6.5	984.14	3.2
Luxembourg	608	3,053.81	5.5	5,024.96	1.3
Malta	484	1,064.06	9.3	2,200.61	2.5
Netherlands	17,231	75,008.25	10.1	4,353.09	1.8
Poland	37,979	30,787.16	6.5	810.65	2.2
Portugal	10,282	17,587.68	9.0	1,710.57	5.6
Romania	19,474	9,749.41	5.2	500.64	2.5
Slovakia	5,447	5,764.15	6.7	1,058.22	3.1
Slovenia	2,067	3,546.49	8.2	1,715.46	2.5
Spain	46,724	104,267.79	8.9	2,231.58	3.8
Sweden	10,183	52,765.82	11.0	5,181.67	4.3
Switzerland	8,517	74,947.83	12.3	8,800.26	4.5
UK	66,489	227,158.96	9.6	3,416.49	2.4
EU27+2	521,730	1,600,824.83	9.4	3,068.30	3.5

The share of health care spending allocated to osteoporosis varied across countries, ranging from 1.3% in Luxembourg to 6.2% in Greece (Table 2). As might be expected, there was a significant but modest positive relationship between the amount spent on osteoporosis, gross domestic product (GDP) and the incidence of osteoporotic fractures.

**Fig. 4:**
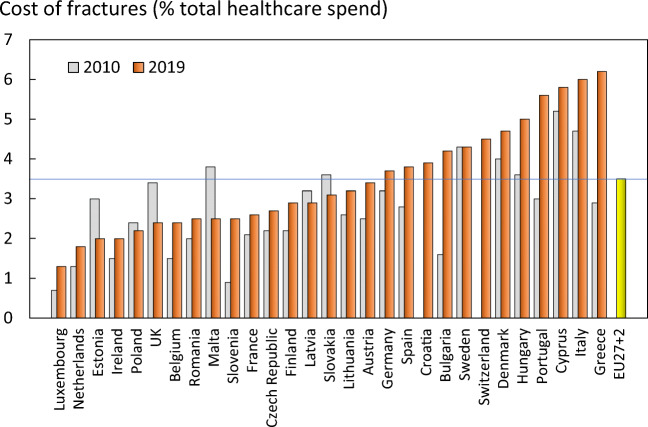
Proportion (%) of the total direct healthcare spend in the EU27+2 countries allocated to osteoporotic fractures. Bars in grey denote values for 2010

The percentage of healthcare spending devoted to osteoporotic fractures has increased in the majority of countries since 2010 (Fig. 4). The most marked increases were seen in Greece (+3.3%), Portugal (+2.6%) and Bulgaria (+2.6%). Of the six counties with a decrease in healthcare spending, the largest reductions in the percentage of healthcare spending for osteoporotic fractures occurred in Malta (−1.3%), Estonia (−1.0%) and the UK (−1.0%).

The estimated cost of osteoporosis may be compared to the cost of other diseases. However, given that the EU27+2 is a relatively new construct, few directly comparable studies exist. Furthermore, methodological differences render some studies difficult to compare. There are a few studies available that were conducted in a similar geographic area, with comparable methodology. A 2010 report issued by the European Brain Council estimated the societal costs for several brain disorders in Europe. After inflating these estimates to 2019 Euros, the annual societal costs were estimated at €118.5 billion for dementia, €49.0 billion for headache, €16.5 billion for multiple sclerosis, and €15.7 billion for Parkinson’s disease [44].

The annual cost of coronary heart disease and cerebrovascular disease in the European Union (25 countries in 2003) has been estimated by Leal et al in 2006 [45]. After adjusting their estimates for inflation, coronary heart disease and cerebrovascular disease in the EU were estimated cost approximately €58.4 billion and €44.1 billion, respectively, in 2019 prices. The cost of epilepsy in the European Union (25 countries in 2004) has been estimated at €15.5 billion, when adjusted to 2019 prices. Healthcare costs were estimated to comprise 18% of costs, whereas direct medical costs and productivity losses represented 27% and 55%, respectively [46]. Thus, in common with other non-communicable diseases, osteoporosis has major economic consequences for society.

#### Comment

It should be noted that not all fracture-related costs come from the countries’ healthcare budgets (e.g. long-term care and variable reimbursement policies). Data on healthcare spending are from 2017.

#### References

40. World Health Organization (WHO) (2020) Indicators and data. WHO. Accessed 2020-05-26 2020 https://apps.who.int/nha/database/Select/Indicators/en
41. Hernlund E, Svedbom A, Ivergard M, Compston J, Cooper C, Stenmark J, McCloskey EV, Jonsson B, Kanis JA (2013) Osteoporosis in the European Union: medical management, epidemiology and economic burden. A report prepared in collaboration with the International Osteoporosis Foundation (IOF) and the European Federation of Pharmaceutical Industry Associations (EFPIA). Arch Osteoporos 8:136
42. Kanis JA, Borgstrom F, Compston J, Dreinhofer K, Nolte E, Jonsson L, Lems WF, McCloskey EV, Rizzoli R, Stenmark J (2013) SCOPE: a scorecard for osteoporosis in Europe. Arch Osteoporos 8:144
43. United Nations (UN) (2020) World Population Prospects 2019. UN https://population.un.org/wpp/ Accessed 9 Apr 2020
44. Gustavsson A, Svensson M, Jacobi F, Allgulander C, Alonso J, Beghi E, Dodel R, Ekman M, Faravelli C, Fratiglioni L, Gannon B, Jones DH, Jennum P, Jordanova A, Jonsson L, Karampampa K, KnappM, Kobelt G, Kurth T, Lieb R, LindeM, Ljungcrantz C, Maercker A, Melin B, Moscarelli M, Musayev A, Norwood F, Preisig M, Pugliatti M, Rehm J, Salvador-Carulla L, Schlehofer B, Simon R, Steinhausen HC, Stovner LJ, Vallat JM, Van den Bergh P, van Os J, Vos P, Xu W, Wittchen HU, Jonsson B, Olesen J (2011) Cost of disorders of the brain in Europe 2010. Eur Neuropsychopharmacol 21:718–779
45. Leal J, Luengo-Fernandez R, Gray A, Petersen S, Rayner M (2006) Economic burden of cardiovascular diseases in the enlarged European Union. Eur Heart J 27:1610–1619
46. Pugliatti M, Beghi E, Forsgren L, Ekman M, Sobocki P (2007) Estimating the cost of epilepsy in Europe: a review with economic modeling. Epilepsia 48:2224–2233

### 1c—Men and women with osteoporosis

#### Domain

Burden of disease—background information

#### Background and aims

Osteoporosis is operationally defined by bone mineral density (BMD) measured using dual-energy X-ray absorptiometry (DXA). The diagnostic reference site is the femoral neck using the NHANES III reference data [47]. Osteoporosis is diagnosed when the BMD measured at the femoral neck is more than 2.5 standard deviations below the average value of the young white female population [48]. The aim of this background information was to document the burden of osteoporosis as judged by densitometric criteria.

#### Methods

Accurate estimates of the prevalence of osteoporosis require country-specific data on the distribution of femoral neck BMD. However, large population-based reference data are lacking in the EU27+2 countries. For the purposes of this report, it is assumed that the mean femoral neck BMD is similar across the EU27+2 countries at the age of 50 years as is the rate of bone loss at the femoral neck with age. The same assumptions have been used elsewhere [49–55]. On this basis, the prevalence of osteoporosis was calculated from the age and sex-specific BMD in the NHANES III study, presented in Hernlund et al 2013 and applied to the current population estimates for people ages 50+ [56, 57]. These prevalence estimates were then applied to the population demography in each country. The densitometric criteria to describe the prevalence of osteoporosis are in line with WHO recommendations [48] but are stricter than those that are commonly used on an operational basis.

#### Results

In 2019, there were approximately 32.0 million individuals with osteoporosis in the EU27+2, of which 6.5 million were men and 25.5 million were women, i.e. there were about four times as many women with osteoporosis as there were men. Of all member states, Germany was estimated to have the highest number of individuals with osteoporosis with approximately 1.2 million men, and 4.5 million women with osteoporosis. According to estimates from Hernlund et al 2013 [56], the prevalence of osteoporosis in the EU27 was 6.6% and 22.1%, respectively in men and women aged 50 years or more (Table 3). Note that proxy estimates from neighbouring countries were used for Croatia and Switzerland. In men over the age of 50 years, the prevalence of osteoporosis varied from 5.7% (Slovakia) to 6.9% (Sweden). In women, the prevalence ranged from 19.3% (Cyprus) to 23.4% (Italy).

**Table 3:** Estimated number and prevalence of men and women with osteoporosis in 2019

Country	Men with osteoporosis	Women with osteoporosis	Men and women with osteoporosis	Prevalence in male population aged 50+ (%)	Prevalence in female population aged 50+ (%)	Prevalence in total population (%)
Austria	113,230	438,894	552,124	6.5	22.2	5.5
Belgium	142,428	538,944	681,372	6.6	22.4	5.6
Bulgaria	82,432	337,744	420,176	6.4	20.9	5.6
Croatia	48,050	204,248	252,298	6.2	21.1	5.5
Cyprus	11,346	38,986	50,332	6.2	19.3	3.7
Czech Republic	114,600	457,776	572,376	6.0	20.4	5.0
Denmark	72,670	254,888	327,558	6.5	21.1	5.1
Estonia	13,082	68,820	81,902	6.2	22.2	5.8
Finland	69,376	266,815	336,191	6.4	21.5	5.7
France	802,660	3,188,700	3,991,360	6.7	22.5	5.5
Germany	1,159,884	4,499,208	5,659,092	6.6	22.6	6.1
Greece	143,796	539,883	683,679	6.9	22.3	5.7
Hungary	99,758	459,347	559,105	6.2	21.1	5.5
Ireland	47,120	162,200	209,320	6.2	20.0	3.7
Italy	878,922	3,479,814	4,358,736	6.9	23.4	6.3
Latvia	18,849	105,925	124,774	6.1	22.3	5.8
Lithuania	28,487	152,117	180,604	6.1	21.7	5.3
Luxembourg	6,466	23,100	29,566	6.1	21.0	4.3
Malta	5,015	18,216	23,232	5.9	19.8	4.9
Netherlands	215,901	760,240	976,141	6.3	20.8	4.9
Poland	367,430	1,617,246	1,984,676	5.8	20.1	4.8
Portugal	133,263	547,360	680,623	6.7	22.0	5.6
Romania	207,390	863,870	1,071,260	6.2	20.5	4.8
Slovakia	50,160	213,788	263,948	5.7	19.4	4.2
Slovenia	24,720	100,190	124,910	6.0	21.5	5.4
Spain	612,272	2,332,998	2,945,270	6.8	22.6	5.4
Sweden	130,755	452,480	583,235	6.9	22.4	5.6
Switzerland	111,210	412,450	523,660	6.6	22.6	6.1
UK	821,152	2,953,653	3,774,805	6.7	21.9	5.2
EU27+2	6,532,426	25,511,028	32,043,453	6.6	22.1	5.6

**Fig. 5:**
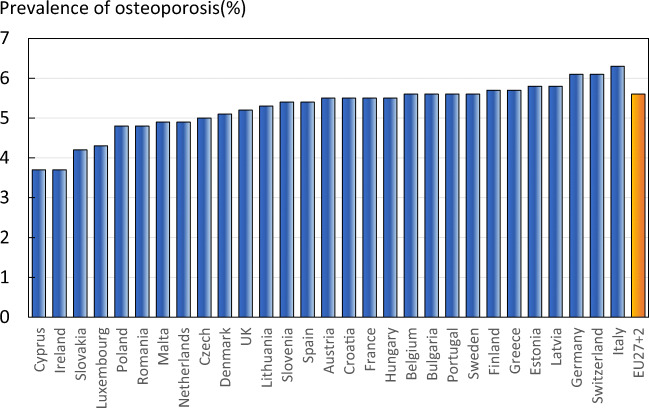
The prevalence of osteoporosis by country, and the average prevalence in the EU27+2

The prevalence of osteoporosis in the entire EU27+2 population (i.e. all ages) was 5.6% and ranged from 3.7% in Cyprus and Ireland, to 6.3% in Italy (Fig. 5).

Since the original SCOPE study, almost every country has experienced an increase in the number of individuals with osteoporosis. The countries with the largest absolute increases are Germany, the UK and Italy with 153.2, 141.7 and 129.7 thousand new women with osteoporosis, respectively. The same countries also had the largest increases in men with osteoporosis. Only one country, Latvia, has seen a decrease in both men and women with osteoporosis. This decrease is likely related to a progressive decline in the population of Latvia since the 1990’s.

#### Score allocation

Chapter 1a, 1b and 1c are not scored and not a score card element.

#### Comment

Although BMD is a strong predictor of fracture risk [58, 59], the prevalence of osteoporosis is not used as a scorecard element because the relationship of osteoporosis to fracture risk varies by age and between countries [60, 61]. For this reason, fracture risk is the preferred metric.

#### References

47. Looker AC, Wahner HW, Dunn WL, Calvo MS, Harris TB, Heyse SP, Johnston CC Jr, Lindsay R (1998) Updated data on proximal femur bone mineral levels of US adults. Osteoporos Int 8:468–489
48. Kanis JA, McCloskey EV, Johansson H, Oden A, Melton LJ 3rd, Khaltaev N (2008) A reference standard for the description of osteoporosis. Bone 42:467–475
49. Borgstrom F, Karlsson L, Ortsater G, Norton N, Halbout P, Cooper C, Lorentzon M, McCloskey EV, Harvey NC, Javaid MK, Kanis JA (2020) Fragility fractures in Europe: burden, management and opportunities. Arch Osteoporos 15:59
50. Cawston H, Maravic M, Fardellone P, Gauthier A, Kanis JA, Compston J, Borgstrom F, Cooper C, McCloskey E (2012) Epidemiological burden of postmenopausal osteoporosis in France from 2010 to 2020: estimations from a disease model. Arch Osteoporos 7:237–246
51. Gauthier A, Kanis JA, Jiang Y, Dreinhofer K, Martin M, Compston J, Borgstrom F, Cooper C, McCloskey E (2012) Burden of postmenopausal osteoporosis in Germany: estimations from a disease model. Arch Osteoporos 7:209–218
52. Gauthier A, Kanis JA, Jiang Y, Martin M, Compston JE, Borgstrom F, Cooper C, McCl o skey EV (2011) Epidemiological burden of postmenopausal osteoporosis in the UK from 2010 to 2021: estimations from a disease model. Arch Osteoporos 6:179–188
53. Gauthier A, Kanis JA, Martin M, Compston J, Borgstrom F, Cooper C, McCloskey E (2011) Development and validation of a disease model for postmenopausal osteoporosis. Osteoporos Int 22:771–780
54. Oden A, McCloskey EV, Johansson H, Kanis JA (2013) Assessing the impact of osteoporosis on the burden of hip fractures. Calcif Tissue Int 92:42–49
55. Strom O, Borgstrom F, Kanis JA, Compston J, Cooper C, McCloskey EV, Jonsson B (2011) Osteoporosis: burden, health care provision and opportunities in the EU: a report prepared in collaboration with the International Osteoporosis Foundation (IOF) and the European Federation of Pharmaceutical Industry Associations (EFPIA). Arch Osteoporos 6:59–155
56. Hernlund E, Svedbom A, Ivergard M, Compston J, Cooper C, Stenmark J, McCloskey EV, Jonsson B, Kanis JA (2013) Osteoporosis in the European Union: medical management, epidemiology and economic burden. A report prepared in collaboration with the International Osteoporosis Foundation (IOF) and the European Federation of Pharmaceutical Industry Associations (EFPIA). Arch Osteoporos 8:136
57. Kanis JA, Borgstrom F, Compston J, Dreinhofer K, Nolte E, Jonsson L, Lems WF, McCloskey EV, Rizzoli R, Stenmark J (2013) SCOPE: a scorecard for osteoporosis in Europe. Arch Osteoporos 8:144
58. Johnell O, Kanis JA, Oden A, Johansson H, De Laet C, Delmas P, Eisman JA, Fujiwara S, Kroger H, Mellstrom D, Meunier PJ, Melton LJ 3rd, O'Neill T, Pols H, Reeve J, Silman A, Tenenhouse A (2005) Predictive value of BMD for hip and other fractures. J Bone Miner Res 20:1185–1194
59. Marshall D, Johnell O,WedelH (1996) Meta-analysis of how well measures of bone mineral density predict occurrence of osteoporotic fractures. Bmj 312:1254–1259
60. Kanis JA, McCloskey EV, Harvey NC, Johansson H, Leslie WD (2015) Intervention Thresholds and the Diagnosis of Osteoporosis. J Bone Miner Res 30:1747–1753
61. Kanis JA, on behalf of the World Health Organization Scientific Group (2008) Assessment of Osteoporosis at the Primary Health Care Level.WHO Scientific Group technical report. University of Sheffield, UK. Accessed April 2020 http://www.shef.ac.uk/FRAX/pdfs/WHO_Technical_Report.pdf

### 1d—Epidemiology of hip fracture

#### Domain

Burden of disease—scorecard element

#### Background and aims

Fracture incidence is scantily documented in the EU. The fracture that has been evaluated most widely is hip fracture. Hip fractures account for the majority of health care expenditure, mortality and morbidity and can be used as a proxy for osteoporosis [62–65]. There is a marked difference in the incidence of hip fracture worldwide and probably in other osteoporotic fractures [66]. Indeed, the difference in incidence between countries within Europe is greater than the differences in incidence between sexes within a country [67, 68]. The EU comprises countries with some of the highest hip fracture rates, but the documentation of the size of the problem and the quality of data vary between countries.

The aim of this scorecard element was to summarise the information base available for the incidence of hip fracture.

#### Methods

Studies on hip fracture risk were identified from 1950 to November 2011 by a Medline OVID search [66]. Evaluable studies in each country were reviewed for quality and representativeness and a study (studies) chosen to represent that country for a previous European report [63, 69]. In essence, the rates used were those incorporated in the various country specific FRAX models. For this report, new hip fracture data were available for Bulgaria [70], Croatia [71], Estonia [72], and Switzerland [73], again rates used in the current FRAX models. Age-specific incidence rates between the ages of 50-89 years were age-standardised to the world population in 2020 in men and in women.

#### Results

National data on hip fracture rates were identified in 18 countries (Table 4). No data were available for 3 countries (Cyprus, Latvia, Luxembourg). In the remaining 8 countries, regional estimates were identified. For Slovenia data were available in women only.

**Table 4:** Age-standardized (2020) hip fracture rates (/100,000/year) in European countries

Country	Year	Sample	Incidence		F/M	Source
			Men	Women		
Austria	2001-5	National	298	608	2.0	[74]
Belgium	2005-7	National	221	471	2.1	[75–77]
Bulgaria	2015-7	Regional	188	411	2.2	[70]
Croatia	2012	National	188	406	2.2	[71]
Cyprus	-	-	-	-	-	
Czech Republic	2008-9	National	248	457	1.8	[66]
Denmark	2004	National	371	677	1.8	[78]
Estonia	2010	National	181	278	1.5	[72]
Finland	2000-6	National	234	382	1.6	[79]
France	2004	National	168	387	2.3	[80]
Germany	2003-4	National	225	470	2.1	[66]
Greece	1986-92	Regional	236	503	2.1	[67, 81–83]
Hungary	1999-2003	National	237	472	2.0	[66]
Ireland	2009-10	National	228	431	1.9	[84]
Italy	2008	National	204	476	2.3	[85]
Latvia	-	-	-	-	-	
Lithuania	2009-10	Regional	199	336	1.7	[86]
Luxembourg	-	-	-	-	-	
Malta	2003-07	National	205	454	2.2	[66]
Netherlands	2005	National	153	290	1.9	[87]
Poland	2008	Regional	149	263	1.8	[88]
Portugal	2006-10	National	149	381	2.6	[89]
Romania	2005-9	National	159	246	1.6	[66]
Slovakia	2007	National	266	451	1.7	[66]
Slovenia	2003	National	-	461	-	[66]
Spain	1984-91	Regional	132	330	2.5	[67, 90–92]
Sweden	1991	Regional	339	730	2.2	[66]
Switzerland	2000	National	227	510	2.2	[73, 93]
UK	1992–3	Regional	185	405	2.2	[66]

**Fig. 6:**
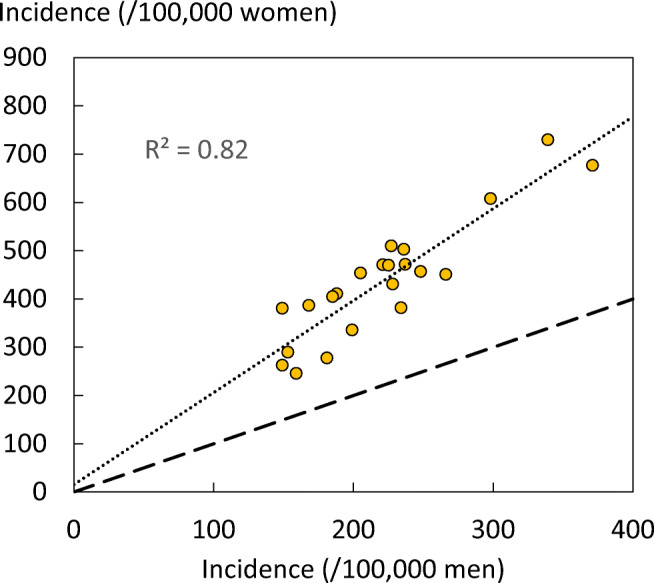
Correlation between men and women in the incidence of hip fracture in European countries. The dotted line shows the linear regression, and the dashed line shows the line of identity

As expected, hip fracture rates were higher in women than in men with a female/male ratio that ranged from 1.5 (Estonia) to 2.6 (Portugal). In women, there was a nearly three-fold range of hip fracture rates throughout the EU from 246/100,000 (Romania) to 730/100,000 (Sweden). In men, rates ranged from 132/100,000 (Spain) to 371/100,000 (Denmark). Thus, the international variation between countries was greater than the differences between men and women within countries (Fig. 6).

#### Score criteria

The age-standardised incidence was ranked. Women were chosen since fracture rates are more robust and it permitted the inclusion of Slovenia for which no data were available in men. The criteria for categorisation were chosen as described in Table 5.

**Table 5:**
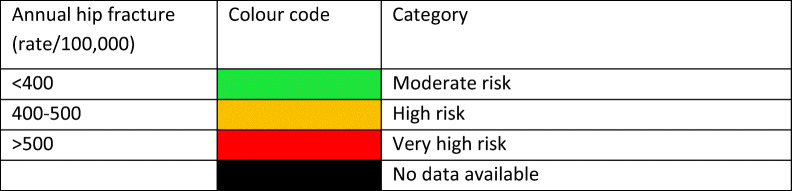
Criteria for allocating scores

#### Score allocation

**Fig. 7:**
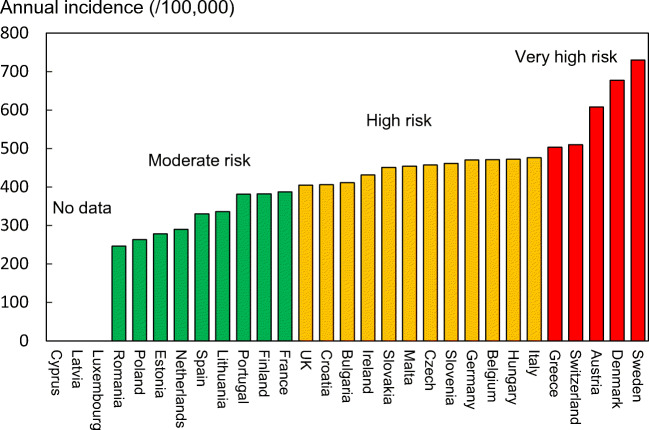
Annual incidence of hip fracture in women from countries of the EU age-standardized to the world population for 2020

The ranked incidence is shown in Fig. 7 and colour coded by category.

#### Comment

On an international scale, all countries were at moderate or high risk (150–250/100,000 and >250/100,000, respectively) [66]. Reasons for the large variation in fracture risk between countries are speculative, but, ecological studies have shown weak but significant relationship between hip fracture risk and latitude and socio-economic prosperity. As noted in the methods, hip fracture risks are those used in FRAX and more recent estimates for some countries will be available for future use.

#### References

62. Borgstrom F, Karlsson L, Ortsater G, Norton N, Halbout P, Cooper C, Lorentzon M, McCloskey EV, Harvey NC, Javaid MK, Kanis JA (2020) Fragility fractures in Europe: burden, management and opportunities. Arch Osteoporos 15:59
63. Kanis JA, Borgstrom F, Compston J, Dreinhofer K, Nolte E, Jonsson L, Lems WF, McCloskey EV, Rizzoli R, Stenmark J (2013) SCOPE: a scorecard for osteoporosis in Europe. Arch Osteoporos 8:144
64. Strom O, Borgstrom F, Kanis JA, Compston J, Cooper C, McCloskey EV, Jonsson B (2011) Osteoporosis: burden, health care provision and opportunities in the EU: a report prepared in collaboration with the International Osteoporosis Foundation (IOF) and the European Federation of Pharmaceutical Industry Associations (EFPIA). Arch Osteoporos 6:59–155
65. Svedbom A, Hernlund E, Ivergard M, Compston J, Cooper C, Stenmark J, McCloskey EV, Jonsson B, Kanis JA (2013) Osteoporosis in the European Union: a compendium of countryspecific reports. Arch Osteoporos 8:137
66. Kanis JA, Oden A, McCloskey EV, Johansson H, Wahl DA, Cooper C (2012) A systematic review of hip fracture incidence and probability of fracture worldwide. Osteoporos Int 23:2239–2256
67. Elffors I, Allander E, Kanis JA, Gullberg B, Johnell O, Dequeker J, Dilsen G, Gennari C, Lopes Vaz AA, Lyritis G et al (1994) The variable incidence of hip fracture in southern Europe: the MEDOS Study. Osteoporos Int 4:253–263
68. Johnell O, Gullberg B, Allander E, Kanis JA (1992) The apparent incidence of hip fracture in Europe: a study of national register sources. Osteoporos Int 2:298–302
69. Hernlund E, Svedbom A, Ivergard M, Compston J, Cooper C, Stenmark J, McCloskey EV, Jonsson B, Kanis JA (2013) Osteoporosis in the European Union: medical management, epidemiology and economic burden. A report prepared in collaboration with the International Osteoporosis Foundation (IOF) and the European Federation of Pharmaceutical Industry Associations (EFPIA). Arch Osteoporos 8:136
70. Kirilova E, Johansson H, Kirilov N, Vladeva S, Petranova T, Kolarov Z, Liu E, Lorentzon M, Vandenput L, Harvey NC, McCloskey E, Kanis JA (2020) Epidemiology of hip fractures in Bulgaria: development of a country-specific FRAX model. Arch Osteoporos 15:28
71. Poljicanin T (2012) Hospital morbidity, Croatian National Institute of Public Health - personal communication. Received by Kanis JA. Hip fracture data from 2012 https://www.sheffield.ac.uk/FRAX/
72. Jurisson M, Vorobjov S, Kallikorm R, Lember M, Uuskula A (2015) The incidence of hip fractures in Estonia, 2005-2012. Osteoporos Int 26:77–84
73. Lippuner K, Johansson H, Kanis JA, Rizzoli R (2009) Remaining lifetime and absolute 10-year probabilities of osteoporotic fracture in Swiss men and women. Osteoporos Int 20:1131–1140
74. Dimai HP (2008) Data from Statistics Austria - personal communication. Received by Kanis JA.
75. Hiligsmann M, Bruyere O, Ethgen O, Gathon HJ, Reginster JY (2008) Lifetime absolute risk of hip and other osteoporotic fracture in Belgian women. Bone 43:991–994
76. Hiligsmann M, Bruyere O, Roberfroid D, Dubois C, Parmentier Y, Carton J, Detilleux J, Gillet P, Reginster JY (2012) Trends in hip fracture incidence and in the prescription of antiosteoporosis medications during the same time period in Belgium (2000-2007). Arthritis Care Res (Hoboken) 64:744–750
77. Johansson H, Kanis JA, McCloskey EV, Oden A, Devogelaer JP, Kaufman JM, Neuprez A, Hiligsmann M, Bruyere O, Reginster JY (2011) A FRAX(R) model for the assessment of fracture probability in Belgium. Osteoporos Int 22:453–461
78. Abrahamsen B, Vestergaard P (2010) Declining incidence of hip fractures and the extent of use of anti-osteoporotic therapy in Denmark 1997-2006. Osteoporos Int 21:373–380
79. Kroger H (2008) Data from The National Research and Development Centre for Welfare and Health - personal communication. Received by Kanis JA. Hip fracture data from 2008 https://www.sheffield.ac.uk/FRAX/
80. Couris CM, Chapurlat RD, Kanis JA, Johansson H, Burlet N, Delmas PD, Schott AM (2012) FRAX(R) probabilities and risk of major osteoporotic fracture in France. Osteoporos Int 23:2321–2327
81. Dretakis EK, Giaourakis G, Steriopoulos K (1992) Increasing incidence of hip fracture in Crete. Acta Orthop Scand 63:150–151
82. Lyritis G (2012) Hip fracture data - Personal communication. Received by Kanis JA. Hip fracture data from 2012 https://www.sheffield.ac.uk/FRAX/
83. Paspati I, Galanos A, Lyritis GP (1998) Hip fracture epidemiology in Greece during 1977-1992. Calcif Tissue Int 62:542–547
84. McGowan B (2010) Hip fracture data: Publication planned - Personal communication. Received by Kanis JA. Hip fracture estimates from 2009-2010 https://www.sheffield.ac.uk/FRAX/
85. Piscitelli P, Chitano G, Johannson H, BrandiML, Kanis JA, Black DM(2013) Updated fracture incidence rates for the Italian version of FRAX(R). Osteoporos Int 24:859–866
86. Tamulaitiene M, Alekna V (2012) Incidence and direct hospitalisation costs of hip fractures in Vilnius, capital of Lithuania, in 2010. BMC Public Health 12:495
87. Lalmohamed A, Welsing PM, Lems WF, Jacobs JW, Kanis JA, Johansson H, De Boer A, De Vries F (2012) Calibration of FRAX (R) 3.1 to the Dutch population with data on the epidemiology of hip fractures. Osteoporos Int 23:861–869
88. Czerwinski E, Kanis JA, Osieleniec J, Kumorek A, Milert A, Johansson H, McCloskey EV, Gorkiewicz M (2011) Evaluation of FRAX to characterise fracture risk in Poland. Osteoporos Int 22:2507–2512
89. MarquesA,Mota A, CanhaoH, Romeu JC, Machado P, Ruano A, Barbosa AP, Dias AA, Silva D, Araujo D, Simoes E, Aguas F, Rosendo I, Silva I, Crespo J, Alves JD, Costa L, Mascarenhas M, Lourenco O, Ferreira PL, Lucas R, Roque R, Branco JC, Tavares V, Johansson H, Kanis J, Pereira da Silva JA (2013) A FRAX model for the estimation of osteoporotic fracture probability in Portugal. Acta Reumatol Port 38:104–112
90. Diez A, Puig J,MartinezMT, Diez JL, Aubia J, Vivancos J (1989) Epidemiology of fractures of the proximal femur associated with osteoporosis in Barcelona, Spain. Calcif Tissue Int 44:382–386
91. Izquierdo Sanchez M, Ochoa Sangrador C, Sanchez Blanco I, Hidalgo Prieto MC, Lozano del Valle F, Martin Gonzalez T (1997) Epidemiology of osteoporotic hip fractures in the province of Zamora (1993). Rev Esp Salud Publica 71:357–367
92. Sosa M, Segarra MC, Hernandez D, Gonzalez A, Liminana JM, Betancor P (1993) Epidemiology of proximal femoral fracture in Gran Canaria (Canary Islands). Age Ageing 22:285–288
93. Lippuner K, GolderM, Greiner R (2005) Epidemiology and direct medical costs of osteoporotic fractures in men and women in Switzerland. Osteoporos Int 16(Suppl 2):S8–s17

### 1e—Number of fragility fractures

#### Domain

Burden of disease—scorecard element

#### Background and aims

The most obvious and serious effect of osteoporosis is the fractures that occur as a consequence of increased bone fragility. This section determines the number of fractures associated with bone fragility in the EU27+2.

#### Methods

The fractures of interest include those at the hip, spine, and forearm as well as fractures at other vulnerable sites (humerus, ribs, tibia, pelvis and other femoral fractures) grouped as other fractures. Information on the incidence of fractures varies between the countries of the EU27+2. In general, reports on hip fracture incidence are more complete than for fractures at other sites (see Chapter 1d). The risks of hip fracture were the same as those used in estimating the FRAX algorithm by country. Hip fracture incidence for Switzerland in 2010 came from an publication that accompanied the original SCOPE study [94]. For the EU27+2 countries with incomplete information, incidence was taken from the nearest country where hip fracture incidence was available [95, 96]; Greek fracture risks were used as a proxy for Cyprus, Finnish fracture risks for Latvia and Belgian fracture risks for Luxembourg. Where the incidence of fractures other than the hip was not available, the incidence was imputed from the hip fracture incidence in the relevant country, using the relationship between hip fracture incidence and incidence of fracture in other sites in Sweden [97]. This assumption has been shown to be safe in studies reported from Canada [98], US [99], UK [100], Australia [101] and Moldova [102], despite marked differences in incidence between these countries [103]. This commonality of pattern is supported by register studies which indicate that, in those regions where hip fracture rates are high, so too is the risk of forearm fracture and spine fractures (requiring hospital admission) [104].

The number of fractures in each country for each fracture site was computed from the age- and sex-specific estimates of incidence and population demography for 2019 [105]. Crude incidence in each country was expressed as the number of fragility fractures per 1000 of the population aged 50 years or more. Where possible country specific data on number of fractures were compared with estimates from 2010 [95, 96].

#### Results

There were estimated to be 4.3 million new fragility fractures in the EU in 2019—equivalent to 11,705 fractures/day (or 487 per hour) (Table 6). About twice as many fractures occurred in women compared to men. Hip, vertebral, forearm and other fractures accounted for 19, 16, 15 and 50% of all fractures, respectively. The number of incident fractures by country is shown in Table 7. Germany had the highest number of fractures for all fracture types in both men and women—approximately 831,000 incident fractures in total—predominately reflecting a large population size and comparatively high fracture incidence. Malta and Luxembourg had the lowest number of fractures for all types—(About 3,200 and 4,000 incident fractures, respectively), reflecting small population sizes.

**Table 6:** Estimated number of incident fractures in the EU27+2 by site, 2019

Fracture site	Women	Men	Women and men
Hip fractures	603,967	222,741	826,708
Vertebral fractures	432,479	230,064	662,544
Forearm fractures	528,109	108,596	636,705
Other fractures	1,293,964	855,626	2,149,591
All fractures	2,858,519	1,417,028	4,275,547

**Table 7:** The number of new fragility fractures in 2019 in men and women by country, the population at risk (men and women aged 50 years or more) and the crude incidence (/1000 of the population)

Country	Fractures in 2010	Fractures in 2019	Population at risk (thousands) 2019	Rate/1000 in 2010	Rate/1000 in 2019
Austria	86536	110196	3719	28.5	29.6
Belgium	79893	100188	4564	20.2	22.0
Bulgaria	38198	56187	2904	13.3	19.3
Croatia	-	34864	1743	-	20.0
Cyprus	5129	6602	385	16.5	17.1
Czech Republic	72195	91349	4154	19	22.0
Denmark	66358	86153	2326	33.1	37.0
Estonia	8688	7870	521	17.9	15.1
Finland	36405	45254	2325	17.4	19.5
France	376774	483654	26152	16.6	18.5
Germany	724774	830848	37482	22	22.2
Greece	85518	99242	4505	20.2	22.0
Hungary	102457	86281	3786	27.8	22.8
Ireland	18085	32367	1571	14.5	20.6
Italy	465400	568424	27609	19.6	20.6
Latvia	14305	15752	784	17.6	20.1
Lithuania	15074	23148	1168	13.4	19.8
Luxembourg	2700	4053	216	17.1	18.8
Malta	2641	3232	177	17.4	18.3
Netherlands	75947	99610	7082	12.9	14.1
Poland	167664	205668	14381	12.6	14.3
Portugal	51821	70730	4477	13.2	15.8
Romania	94282	103035	7559	12.9	13.6
Slovakia	38634**	75722	1982	22.3	38.2
Slovenia	15510	16637	878	20.4	18.9
Spain	204151	285494	19327	12.8	14.8
Sweden	107046	123523	3915	30.7	31.6
Switzerland	74192	82488	3510	24.4	23.5
UK	447972*	526974	25743	20.7	20.5
EU27+2	-	4275547	214945	19.0	19.9

*The estimated fractures for 2010 in the UK were corrected from the original SCOPE study, due overestimation of the “other”-fractures category. The new estimates are based on the original UK hip fracture source, presented in Svedbom et al 2013 [106] and calculated using the same methods described in Chapter 3 of Hernlund et al 2013 [95]

**The large difference is because the estimate in 2010 relied on less accurate data. The data for 2019 uses new and more appropriate data on the incidence of hip fracture

When fracture numbers were expressed as a rate of the population at risk, there was a 3-fold range in risk that varied from 14.1/1000 in the Netherlands to 37.0/1000 in Denmark.

**Fig. 8:**
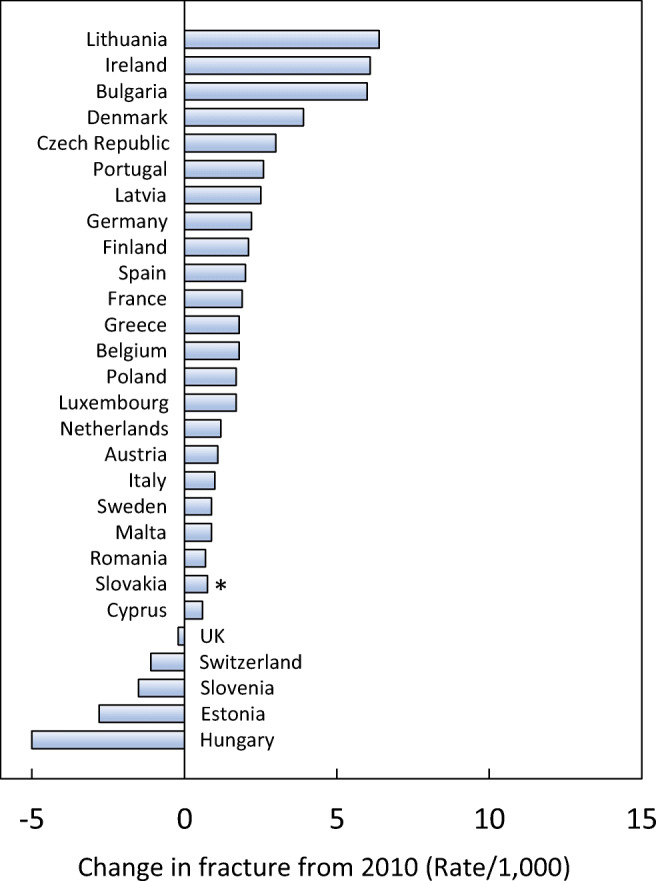
Change in crude incidence (annual rate/1000) of fragility fractures between 2010 and 2019. *Data for Slovakia imputed

Since 2010, the incidence for all fracture sites was estimated to have increased. Hip fracture incidence for both sexes increased the most (33%), whilst the smallest percentage increase was noted for the incidence of distal forearm fracture (14%). The rates of fractures per 1000 people in the population increased in the majority of countries since 2010. The rates were estimated to have increased most in Slovakia (+15.9/1000), Switzerland (+7.3/1000), Lithuania (+6.4/1000) and Ireland (+6.1/1000). Of the four countries where rates had decreased since 2010, the greatest changes were seen in Hungary (-5.0/1000), Estonia (-2.8/1000) and Slovenia (-1.5/1000) (Fig. 8).

**Fig. 9:**
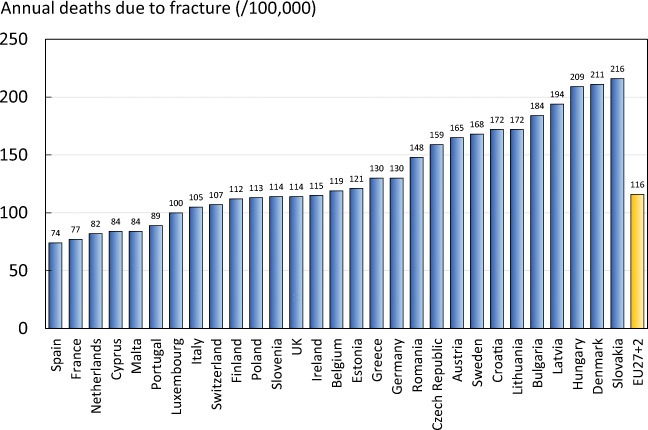
The number of deaths associated with fracture events expressed per 100,000 of the population age 50 years or more in the EU27+2

In addition to pain and disability, some fractures are associated with premature mortality. About 30% of deaths after a hip or clinical spine fracture can be attributed to the fracture event [107–109]. In the EU27+2, there were estimated to be 248,487 causally related deaths in 2019. Approximately 43% of fracture-related deaths in women were due to hip fractures, 53% to clinical vertebral and 3% to other fractures. Corresponding proportions for men were 34, 65 and 1%, respectively. Fracture-related deaths per 100,000 by country are shown in Fig. 9. Note that the variability in death rates is more a reflection of the variable incidence of fractures rather than in standards of care.

**Fig. 10:**
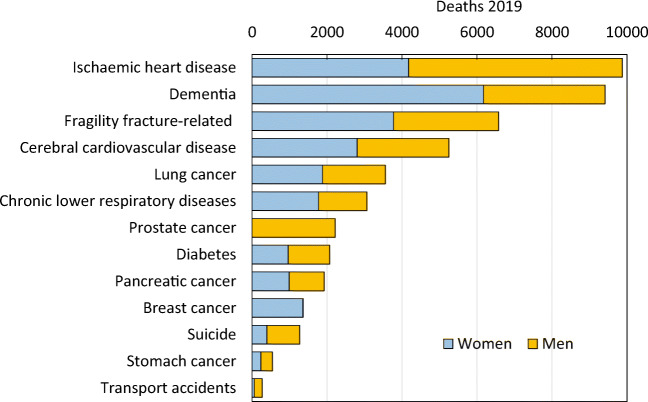
Comparison of the number of causally related deaths due to fracture compared with other causes of death in Sweden in 2019

In order to compare fracture-related deaths to deaths from other causes, estimates on causes of death in Sweden were retrieved from statistics published by the Swedish National Board of Health and Welfare (Socialstyrelsen) [110]. Figure 10 presents a comparison of major causes of death in 2019 to the number of fragility fracture-related deaths in Sweden. As can be seen in the table, the number of fracture-related deaths is comparable or exceeds some of the most common causes of death in Sweden. A similar comparison has been made previously [109]. It should be noted that Sweden is a high-risk country for fractures and associated deaths. Thus, the relative importance of deaths due to osteoporosis compared with other causes of death is likely to vary in different countries.

#### Score criteria

The number of fragility fractures in men and women combined in 2019 expressed/1000 of the population aged 50 years or more was categorised approximately by tertiles as given in Table 8.

**Table 8:**
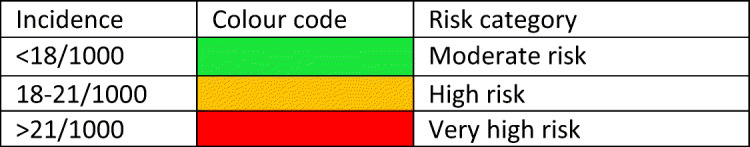
Criteria for allocating scores

#### Score allocation

**Fig. 11:**
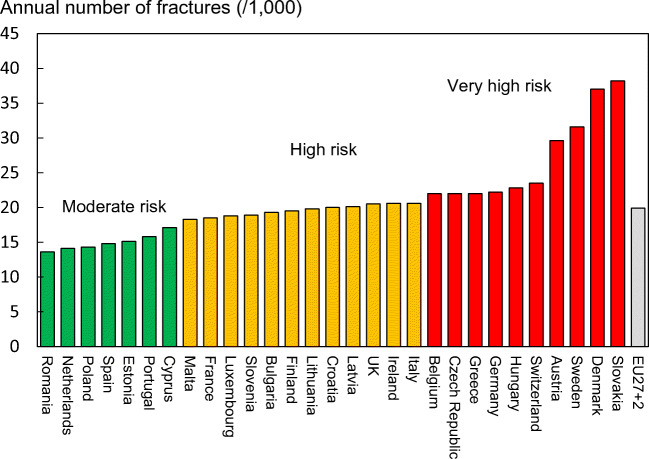
The annual number of fragility fractures in men and women combined expressed/1000 of the population aged 50 years or more, in 2019

Countries, ranked and categorised by risk, are shown in Fig. 11. The variation between countries reflects both the fracture risk and the distribution of age and sex in each country.

#### Comment

These estimates do not include individuals who in 2019 were suffering the consequences of fractures sustained in previous years. There are important data gaps in the documentation of the fracture burden between member states which form the component of a further scorecard element (*Chapter 2a*).

#### References

94. Svedbom A, IvergardM, Hernlund E, Rizzoli R, Kanis JA (2014) Epidemiology and economic burden of osteoporosis in Switzerland. Arch Osteoporos 9:187
95. Hernlund E, Svedbom A, Ivergard M, Compston J, Cooper C, Stenmark J, McCloskey EV, Jonsson B, Kanis JA (2013) Osteoporosis in the European Union: medical management, epidemiology and economic burden. A report prepared in collaboration with the International Osteoporosis Foundation (IOF) and the European Federation of Pharmaceutical Industry Associations (EFPIA). Arch Osteoporos 8:136
96. Kanis JA, Borgstrom F, Compston J, Dreinhofer K, Nolte E, Jonsson L, Lems WF, McCloskey EV, Rizzoli R, Stenmark J (2013) SCOPE: a scorecard for osteoporosis in Europe. Arch Osteoporos 8:144
97. Kanis JA, Oden A, Johnell O, Jonsson B, de Laet C, Dawson A (2001) The burden of osteoporotic fractures: a method for setting intervention thresholds. Osteoporos Int 12:417–427
98. Lam A, LeslieWD, Lix LM, Yogendran M, Morin SN, Majumdar SR (2014) Major osteoporotic to hip fracture ratios in canadian men and women with Swedish comparisons: a population-based analysis. J Bone Miner Res 29:1067–1073
99. Melton LJ 3rd, Crowson CS, O'Fallon WM (1999) Fracture incidence in Olmsted County, Minnesota: comparison of urban with rural rates and changes in urban rates over time. Osteoporos Int 9:29–37
100. Singer BR, McLauchlan GJ, Robinson CM, Christie J (1998) Epidemiology of fractures in 15,000 adults: the influence of age and gender. J Bone Joint Surg Br 80:243–248
101. Sanders KM, Seeman E, Ugoni AM, Pasco JA, Martin TJ, Skoric B, Nicholson GC, Kotowicz MA (1999) Age- and gender-specific rate of fractures in Australia: a population-based study. Osteoporos Int 10:240–247
102. Zakroyeva A, Lesnyak O, Cazac V, Groppa L, Russu E, Chislari L, Rotaru L, Johansson H, Harvey NC, McCloskey E, Lorentzon M, Kanis JA (2020) Epidemiology of osteoporotic fracture in Moldova and development of a country-specific FRAX model. Arch Osteoporos 15:13
103. Kanis JA, Oden A, McCloskey EV, Johansson H, Wahl DA, Cooper C (2012) A systematic review of hip fracture incidence and probability of fracture worldwide. Osteoporos Int 23:2239–2256
104. Johnell O, Gullberg B, Kanis JA (1997) The hospital burden of vertebral fracture in Europe: a study of national register sources. Osteoporos Int 7:138–144
105. World Health Organization (WHO) (2020) Indicators and data. WHO. Accessed 2020-05-26 2020 https://apps.who.int/nha/database/Select/Indicators/en
106. Svedbom A, Hernlund E, Ivergard M, Compston J, Cooper C, Stenmark J, McCloskey EV, Jonsson B, Kanis JA (2013) Osteoporosis in the European Union: a compendium of countryspecific reports. Arch Osteoporos 8:137
107. Johnell O, Kanis JA, Oden A, Sernbo I, Redlund-Johnell I, Petterson C, De Laet C, Jonsson B (2004) Mortality after osteoporotic fractures. Osteoporos Int 15:38–42
108. Kanis JA, Oden A, Johnell O, De Laet C, Jonsson B (2004) Excess mortality after hospitalisation for vertebral fracture. Osteoporos Int 15:108–112
109. Kanis JA, Oden A, Johnell O, De Laet C, Jonsson B, Oglesby AK (2003) The components of excess mortality after hip fracture. Bone 32:468–473
110. Socialstyrelsen, (SoS) (2019) Statistik om dödsorsaker. SoS. Accessed 2020-06-10 2020 https://www.socialstyrelsen.se/statistik-och-data/statistik/statistikamnen/dodsorsaker/

### 1f—Lifetime hip fracture probability

#### Domain

Burden of disease—scorecard element

#### Background and aims

The most serious consequence of osteoporosis in terms of morbidity, mortality and health care expenditure is hip fracture. In the EU, for example, hip fractures comprise only 17% of the total number of fragility fractures but account for 54% of the direct costs and 49% of deaths due to fracture [111–113].

The aim of this element is to provide estimates of the remaining lifetime probability of hip fracture in men and women at the age of 50 and 70 years.

#### Methods

Hip fracture probability was computed, taking both the risk of fracture and the risk of death into account [114]. The risk of hip fracture was updated from a systematic review of hip fracture incidence (see *Chapter 1d*) [115]. Where possible, the incidence of hip fracture was determined in men and women using 5-year age categories. Where 5-year age intervals were not available, 10-year intervals were used (intervals of greater than 10 years were an exclusion criterion). Mortality statistics from the WHO were used in 5 or 10 year age intervals for the year 2019 [116]. The remaining lifetime probabilities were calculated in men and women from the age of 50 and 70 years [117].

#### Results

Empirical data on hip fracture probabilities were available for 26 of the 29 countries (Table 9). No data were available for Cyprus, Latvia or Luxembourg. Data were available only for women from Slovenia.

**Table 9:** Remaining lifetime probability of hip fracture (%) at the ages of 50 and 70 years in men and women by country [113]

Country	Lifetime probability (%)			
	At age 50 years		At age 70 years	
	Men	Women	Men	Women
Austria	8.3	19.7	8.8	20.7
Belgium	7.8	18.2	8.3	18.9
Bulgaria	4.4	11.2	4.3	11.5
Croatia	5.1	11.4	5.1	11.3
Cyprus	-	-	-	-
Czech Republic	6.9	14.8	7.5	15.6
Denmark	10.6	22.1	11.1	23.6
Estonia	4.4	9.1	4.9	9.6
Finland	5.8	12.4	6.1	12.8
France	5.6	18.4	6.3	19.4
Germany	5.3	14.2	5.6	15.0
Greece	8	15.8	8.6	15.2
Hungary	4.1	10.6	5.2	12.0
Ireland	7.8	18.2	8.0	18.7
Italy	7.7	19.2	7.8	19.3
Latvia	-	-	-	-
Lithuania	4.4	11.3	5.3	11.9
Luxembourg	-	-	-	-
Malta	5.8	14.2	5.8	14.2
Netherlands	5.4	12.5	5.6	12.8
Poland	4.0	9.7	3.9	10.1
Portugal	4.8	14.4	5.3	14.9
Romania	3.8	7.0	3.7	7.2
Slovakia	9.5	20.3	9.9	20.3
Slovenia	-	11.6	-	12.0
Spain	4.0	12.1	4.3	12.6
Sweden	10.9	25.1	11.0	25.4
Switzerland	7.1	22.5	6.9	23.5
UK	4.8	13.8	5.0	14.6
EU27+2*	5.7	15.0	6.0	15.6

*The weighted averages for the EU27+2 only include countries for where estimates were available

The average remaining lifetime probability of hip fracture in women at the age of 50 years ranged from 7.0% (Romania) to 25.1% (Sweden). Thus, there was approximately a three-fold range of lifetime probabilities between countries. The average lifetime probability of hip fracture (weighted for population size) at the age of 50 years in the EU was 5.7% in men and 15.0% in women.

**Fig. 12:**
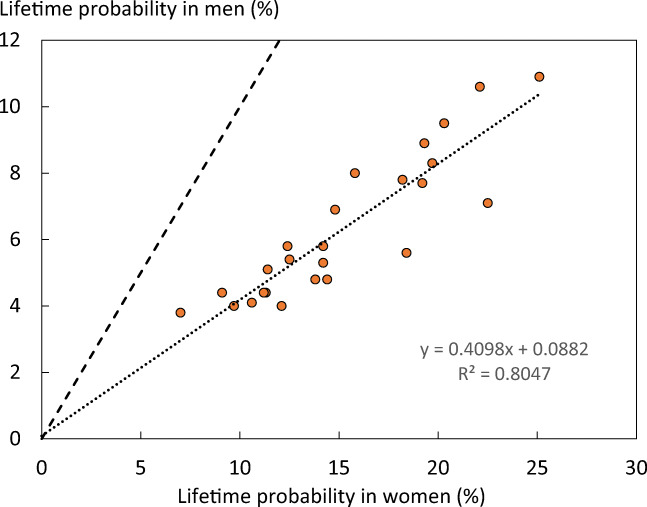
Remaining lifetime probability of hip fracture (%) in men and women from the age of 50 years. The dotted line shows the linear regression, and the dashed line shows the line of identity

Probabilities of hip fracture were approximately twofold lower in men than in women. In men, hip fracture probability at the age of 50 years ranged from 3.8% (Romania) to 10.9% (Sweden). There was a close correlation between hip fracture probability in men and women so that, in those countries where fracture probability was high in women, so too was it high in men (Fig. 12). In Sweden, which had the highest hip fracture probabilities, the hip fracture risk in men (10.9%) was higher than the hip fracture probability in women from Hungary, Poland or Romania.

#### Score criteria

The remaining lifetime probability of hip fracture at the age of 50 years was ranked. Women were chosen since this permitted the inclusion of Slovenia for which no data were available in men. The criteria for categorisation are shown in Table 10.

**Table 10:**
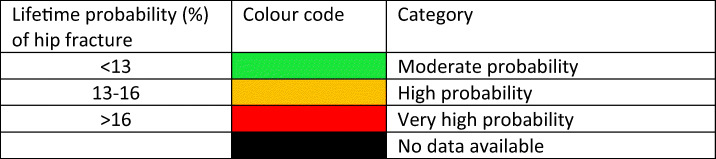
Criteria for allocating scores

#### Score allocation

**Fig. 13:**
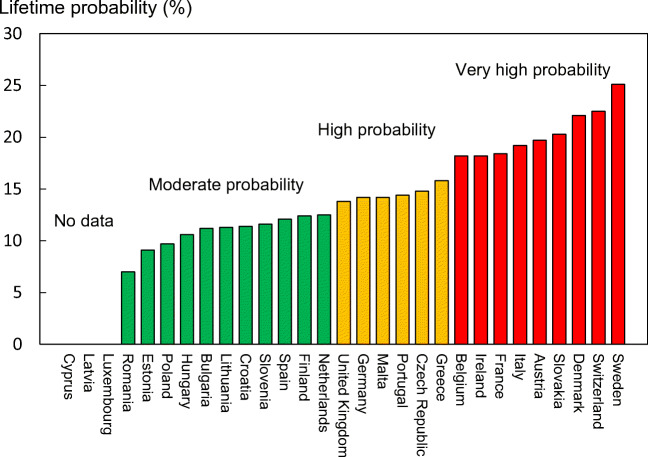
Remaining lifetime probability of hip fracture (%) in women in the EU27+2 from the age of 50 years

The ranked incidence is shown in Fig. 13 and colour coded by category.

#### Comment

Hip fracture probabilities from the age of 70 years were not markedly different from those from the age of 50 years. The reason for this is that increasing death and fracture hazards with age compete in the determination of probability.

#### References

111. Borgstrom F, Karlsson L, Ortsater G, Norton N, Halbout P, Cooper C, Lorentzon M, McCloskey EV, Harvey NC, Javaid MK, Kanis JA (2020) Fragility fractures in Europe: burden, management and opportunities. Arch Osteoporos 15:59
112. Hernlund E, Svedbom A, Ivergard M, Compston J, Cooper C, Stenmark J, McCloskey EV, Jonsson B, Kanis JA (2013) Osteoporosis in the European Union: medical management, epidemiologyand economic burden. A report prepared in collaboration with the International Osteoporosis Foundation (IOF) and theEuropean Federation of Pharmaceutical Industry Associations(EFPIA). Arch Osteoporos 8:136
113. Kanis JA, Borgstrom F, Compston J, Dreinhofer K, Nolte E, Jonsson L, Lems WF, McCloskey EV, Rizzoli R, Stenmark J (2013) SCOPE: a scorecard for osteoporosis in Europe. Arch Osteoporos 8:144
114. Kanis JA, Johnell O, Oden A, Johansson H, McCloskey E (2008) FRAX and the assessment of fracture probability in men and women from the UK. Osteoporos Int 19:385–397
115. Kanis JA, Oden A, McCloskey EV, Johansson H, Wahl DA, Cooper C (2012) A systematic review of hip fracture incidence and probability of fracture worldwide. Osteoporos Int 23:2239–2256
116. United Nations (UN) (2020) World Population Prospects 2019. UN. Accessed 2020-04-09 2020 https://population.un.org/wpp/
117. Kanis JA, Johnell O, Oden A, Sembo I, Redlund-Johnell I, Dawson A, De Laet C, Jonsson B (2000) Long-term risk of osteoporotic fracture in Malmö. Osteoporos Int 11:669–674

### 1g—Men and women at high fracture risk

#### Domain

Burden of disease—scorecard element

#### Background and aims

The advent of FRAX in 2008 provided a clinical tool for the calculation of fracture probability [118, 119]. Probability-based assessment is increasingly being incorporated into clinical guidelines in Europe [120, 121] and elsewhere [122, 123]. Unlike fracture incidence, the probability of fracture at any given age depends upon the hazard of death as well as the hazard of fracture over a defined interval (e.g. 10 years or lifetime). A major advantage of using fracture probability is that it standardises the output from the multiple risk factors that contribute to fracture risk including BMD to be incorporated as a single metric. FRAX models are also calibrated to country-specific epidemiology.

The ability to compute fracture probabilities in individuals permits an estimate of the prevalence of high-risk individuals within a given population where the population demography and the distribution of FRAX-based probabilities are known.

The aim of this score card element was to present the burden of osteoporosis in men and women in the EU 27+2 countries expressed as the proportion of the population with a 10-year probability of a major osteoporotic fracture (hip, spine, forearm or humerus) above a given threshold.

#### Methods

There is no international standard to define high fracture risk based on probabilities. In Europe, intervention thresholds are commonly defined as the ten-year probability of a major fracture that equals or exceeds that of a woman with a prior fragility fracture [120, 123–125], termed the probability fracture threshold. These country specific thresholds are used to determine treatment gaps in Europe (see *Chapters 4c and 4e*). In North America threshold risks have been set at probabilities of 10% and 20% [126, 127] and these were also used for this assessment.

The majority of EU member states have a country specific FRAX model. Where unavailable, a surrogate country was used, based on the assumption that the epidemiology of hip fracture was similar. For Cyprus, Malta was used; for Latvia, Lithuania was used; for Luxembourg, Belgium was used; and for Slovenia, Hungary was used.

The distribution of FRAX probabilities in men and women was simulated in 5-year age intervals for each member state between the ages of 50 to 89 years [128, 129] and applied to the demography of each country for 2019 [130]. Burden of disease was expressed as the number of men and women with a probability of major osteoporotic fracture above a threshold of 10% or 20%. Additionally, the number of men and women with a probability of major osteoporotic fracture above the country-specific thresholds for high risk and very high risk was determined [131]. For comparative purposes, the burden was expressed as the proportion of the population age 50-89 years with probabilities above these thresholds.

The number of men and women with a probability of major osteoporotic fracture above a threshold of 10% or 20% and with a probability of major osteoporotic fracture above the threshold for high risk was compared to data for 2010 in those countries where there was matching information [124, 125].

#### Results

It is estimated that approximately 15.6 million men and women in the EU27+2 had a 10-year fracture probability that was 20% or more. When a 10% threshold was used, the population at or over this risk rose to 49.3 million, representing respectively, 3% and 11% of the total EU population for 2019. With regard to age-specific thresholds, 23.9 million men and women had fracture probabilities equal to or greater than that of age matched women with a prior fragility fracture.

**Fig. 14:**
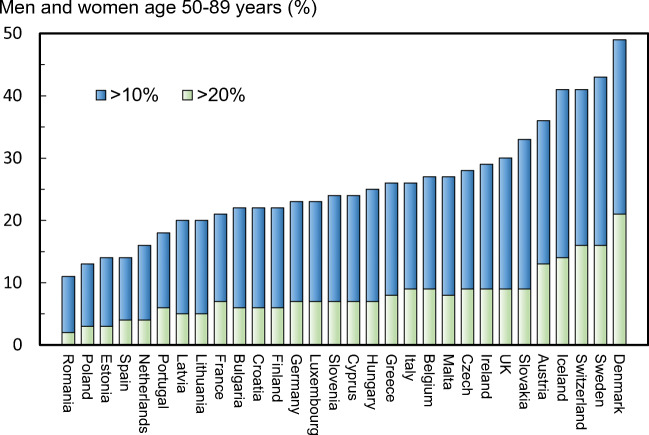
Proportion of men and women (%) aged 50–89 years with a 10-year probability of a major fracture that is 10% or more or 20% or more by member state

The proportion of the population aged 50 years or more that in 2019 had a fracture probability of 20% or more varied among member EU states, ranging from 2% in Romania to 21% in Denmark (Table 11). The proportion of the population aged 50 years or more that had a fracture probability of 10% or more ranged from 11% in Romania to 49% in Denmark (Table 11). Figure 14 shows the rank order of population burden.

**Table 11:** Number of men and women (thousands) and proportion of the population aged 50–89 years (%) with a 10-year probability of a major fracture that exceeds 10%, 20% or the country specific fracture threshold for high and very high risk

Country	Number of men and women (000)				Proportion of population age 50–89 years (%)			
	>20%	>10%	>High risk	>Very high risk	>20%	>10%	>High risk	>Very high risk
Austria	469	1297	386	234	13	36	11	6
Belgium	384	1185	512	315	9	27	12	7
Bulgaria	165	622	325	200	6	22	11	7
Croatia	103	371	196	120	6	22	11	7
Cyprus*	26	91	44	28	7	24	12	7
Czech	361	1144	471	288	9	28	12	7
Denmark	470	1116	252	148	21	49	11	6
Estonia	17	72	60	38	3	14	12	7
Finland	133	493	261	153	6	22	11	7
France	1856	5259	2892	1803	7	21	11	7
Germany	2719	8457	3846	2390	7	23	11	7
Greece	352	1158	534	336	8	26	12	8
Hungary	259	925	431	260	7	25	12	7
Ireland	145	446	179	109	9	29	12	7
Italy	2452	7024	3278	2073	9	26	12	8
Latvia*	39	151	94	58	5	20	12	8
Lithuania	58	223	138	86	5	20	12	8
Luxembourg*	15	49	24	14	7	23	11	7
Malta	14	47	20	13	8	27	12	8
Netherlands	277	1115	801	501	4	16	12	7
Poland	436	1820	1633	1018	3	13	12	7
Portugal	252	786	537	346	6	18	12	8
Romania	135	822	841	525	2	11	11	7
Slovakia	170	642	217	131	9	33	11	7
Slovenia*	56	202	93	56	7	23	11	6
Spain	753	2626	2113	1337	4	14	11	7
Sweden	616	1643	441	268	16	43	12	7
Switzerland	554	1397	375	215	16	41	11	6
UK	2280	7419	2858	1723	9	30	11	7
EU27+2	15566	48602	23852	14786	7	23	11	7

*Surrogate country used (see ,Methods)

The table also shows the number of men and women who lie above a fracture threshold for high risk (and very high risk) commonly used in assessment guidelines. Of the countries surveyed, 23.8 million men and women had a probability of major fracture above the thresholds for high risk and 14.8 million above the thresholds for very high risk. The country-specific data on high risk and very high risk are considered later in relationship to the uptake of treatments in the European Union.

Of the 27 countries of the EU surveyed in 2010, 12.9 million men and women had a 10-year probability of major fracture at or above 20%. In 2019, this had increased to 16.9 million, a rise of 31%. Those with probabilities that exceeded a 10% threshold rose by 18% and those above a high-risk threshold increased by 11% (Table 12).

**Table 12:** Number of men and women (thousands) age 50-89 years with a 10-year probability of a major fracture that exceeded 10%, 20% in 2010 and 2019

Probability	2010	2019	Difference	%
>20%	12,915	16,934	4,019	+31
>10%	41,238	48,899	7,661	+18
High risk threshold	21,339	23,721	2,382	+11

#### Score criteria

Countries were ranked by tertiles of prevalence of the population aged 50–89 years above a 10% probability threshold of a major osteoporotic fracture as given in Table 13.

**Table 13:**
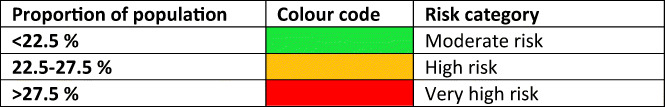
Criteria for allocating scores

#### Score allocation

**Fig. 15:**
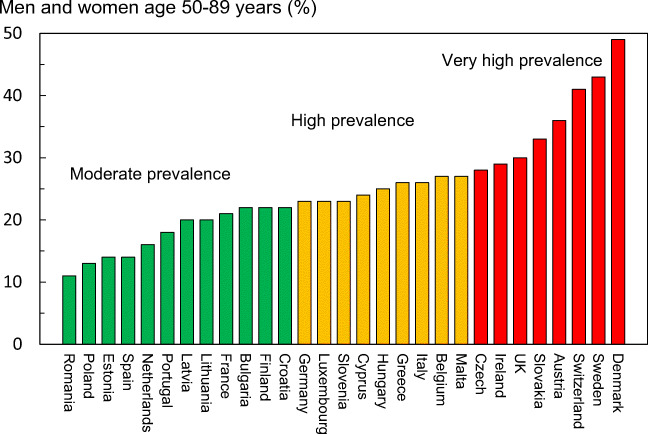
The proportion of the population (%) aged 50–89 years with a 10-year probability of a major fracture that is 10% or more by member state

The proportion of the population (%) age 50–89 years with a 10-year probability of a major fracture that is 10% or more by member state is shown by category and rank in Fig. 15.

#### References

118. Kanis JA, Harvey NC, Johansson H, Liu E, Vandenput L, Lorentzon M, Leslie WD, McCloskey EV (2020) A decade of FRAX: how has it changed the management of osteoporosis? Aging Clin Exp Res 32:187–196
119. Kanis JA, On behalf of the World Health Organization Scientific Group (2008) Assessment of Osteoporosis at the Primary Health Care Level.WHO Scientific Group technical report. University of Sheffield, UK. Accessed April 2020 http://www.shef.ac.uk/FRAX/pdfs/WHO_Technical_Report.pdf
120. Kanis JA, Cooper C, Rizzoli R, Reginster JY, On, behalf of the Scientific Advisory Board of the European Society for Clinical and Economic Aspects of Osteoporosis (ESCEO) and the Committees of Scientific Advisors and National Societies of the International Osteoporosis Foundation (IOF) (2019) European guidance for the diagnosis and management of osteoporosis in postmenopausal women. Osteoporos Int 30:3–44
121. Lekamwasam S, Adachi JD, Agnusdei D, Bilezikian J, Boonen S, Borgstrom F, Cooper C, Diez Perez A, Eastell R, Hofbauer LC, Kanis JA, Langdahl BL, Lesnyak O, Lorenc R, McCloskey E, Messina OD, Napoli N, Obermayer-Pietsch B, Ralston SH, Sambrook PN, Silverman S, Sosa M, Stepan J, Suppan G, Wahl DA, Compston JE (2012) A framework for the development of guidelines for the management of glucocorticoid-induced osteoporosis. Osteoporos Int 23:2257–2276
122. Clark P, Denova-Gutierrez E, Zerbini C, Sanchez A, Messina O, Jaller JJ, Campusano C, Orces CH, Riera G, Johansson H, Kanis JA (2018) FRAX-based intervention and assessment thresholds in seven Latin American countries. Osteoporos Int 29:707–715
123. Kanis JA, Harvey NC, Cooper C, Johansson H, Oden A, McCloskey EV (2016) A systematic review of intervention thresholds based on FRAX : A report prepared for the National Osteoporosis Guideline Group and the International Osteoporosis Foundation. Arch Osteoporos 11:25
124. Hernlund E, Svedbom A, Ivergard M, Compston J, Cooper C, Stenmark J, McCloskey EV, Jonsson B, Kanis JA (2013) Osteoporosis in the European Union: medical management, epidemiology and economic burden. A report prepared in collaboration with the International Osteoporosis Foundation (IOF) and the European Federation of Pharmaceutical Industry Associations (EFPIA). Arch Osteoporos 8:136
125. Kanis JA, Borgstrom F, Compston J, Dreinhofer K, Nolte E, Jonsson L, Lems WF, McCloskey EV, Rizzoli R, Stenmark J (2013) SCOPE: a scorecard for osteoporosis in Europe. Arch Osteoporos 8:144
126. Papaioannou A, Morin S, Cheung AM, Atkinson S, Brown JP, Feldman S, Hanley DA, Hodsman A, Jamal SA, Kaiser SM, Kvern B, Siminoski K, Leslie WD (2010) 2010 clinical practice guidelines for the diagnosis and management of osteoporosis in Canada: summary. Cmaj 182:1864–1873
127. Cosman F, de Beur SJ, LeBoff MS, Lewiecki EM, Tanner B, Randall S, Lindsay R (2014) Clinician's Guide to Prevention and Treatment of Osteoporosis. Osteoporos Int 25:2359–2381
128. Kanis JA, Johansson H, Oden A, McCloskey EV (2012) The distribution of FRAX((R))-based probabilities in women from Japan. J Bone Miner Metab 30:700–705
129. Kanis JAJH, Harvey NC, Gudnason V, Sigurdsson G, Siggeirsdottir K, Lorentzon M, Liu M, Vandenput L, McCloskey E (2020) Effects of the recency of sentinel fractures on conventional estimates of fracture probability using FRAX. Osteoporos Int
130. United Nations (UN) (2020) World Population Prospects 2019. UN. Accessed 2020-04-09 2020. https://population.un.org/wpp/
131. Kanis JA, Harvey NC, McCloskey E, Bruyere O, Veronese N, Lorentzon M, Cooper C, Rizzoli R, Adib G, Al-Daghri N, Campusano C, Chandran M, Dawson-Hughes B, Javaid K, Jiwa F, Johansson H, Lee JK, Liu E, Messina D, Mkinsi O, Pinto D, Prieto-Alhambra D, Saag K, Xia W, Zakraoui L, Reginster J (2020) Algorithm for the management of patients at low, high and very high risk of osteoporotic fractures. Osteoporos Int 31: 1–12

### 1h— Population projections

#### Domain

Burden of disease—background information

#### Background and aims

Secular changes in life expectancy and birth rate are likely to increase the number of elderly individuals in the EU27+2 countries and thereby increase the need for resource allocation for diseases associated with ageing. The incidence of fragility fractures increases markedly with age, particularly in women. The aim of this background element is to estimate the increase in number of men and women aged 50 years or more in the EU27+2 countries.

#### Methods

The age and sex distribution of the EU27+2 countries was obtained from the UN for 2019 and 2034 using the medium variant [132]. Comparable data for 2010 were also retrieved [133, 134].

#### Results

**Fig. 16:**
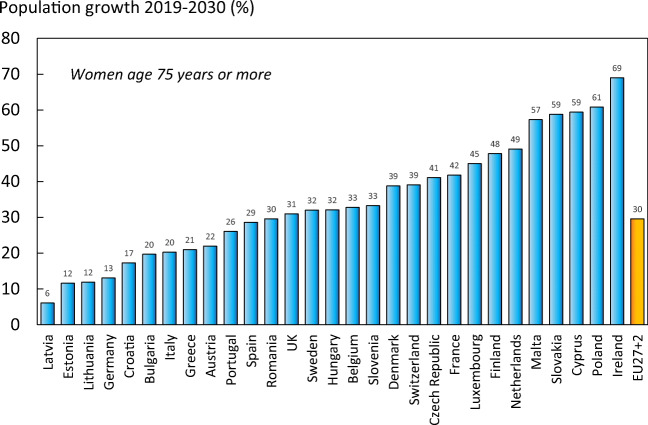
increase by country in the female population aged 75 years or more (%) between 2010 and 2034

Between 2010 and 2019, the population of women over 50 years in the EU27+2 had increased by 22%, and in men by 17%. The population is expected to further increase by 10% for women and 13% for men between 2019 and 2034. The number of men and women aged 50 years, or more will increase in all countries except in Latvia, Lithuania and Bulgaria (Table 14). In the remaining countries, the increment in the population varied widely. For all countries, the percentage increase in number of men and women aged 75 years or more was greater than that of the population aged 50–74 years in the EU27+2 countries. For women aged 75 years or more, the change in the population ranged from less than 12% in Latvia (6%) and Estonia (12%) to more than 50% in Ireland (69%) and Poland (61%) (Fig. 16).

**Table 14:** Projected percentage change in the male and female populations between 2019 and 2034 according to age category

Country	Women (%)		Men (%)		Both (%)
	Age 50–74	Age 75+	Age 50–74	Age 75+	Age 50+
Austria	6.0	22.0	7.5	38.0	11.8
Belgium	3.0	32.8	5.0	55.5	12.6
Bulgaria	−8.4	19.7	−1.8	20.1	−0.1
Croatia	-5.2	17.3	−2.2	41.0	2.8
Cyprus	28.2	59.4	25.3	71.0	33.8
Czech Republic	7.1	41.1	14.4	60.9	18.5
Denmark	−2.6	38.8	−3.4	48.2	7.0
Estonia	−3.3	11.6	10.8	41.0	7.3
Finland	−8.9	47.8	−6.3	66.7	6.9
France	0.3	41.8	0.9	57.0	11.8
Germany	−2.1	13.1	−0.8	25.0	3.5
Greece	6.1	21.0	10.5	23.7	11.9
Hungary	−1.6	32.1	7.0	49.3	9.8
Ireland	31.1	69.0	28.8	78.9	38.0
Italy	3.6	20.3	6.2	31.8	10.1
Latvia	−9.9	6.1	−4.1	18.9	−3.1
Lithuania	−11.6	11.9	−7.7	17.2	−4.2
Luxembourg	24.9	45.0	22.4	81.2	30.3
Malta	−1.7	57.3	3.5	76.2	14.7
Netherlands	−2.7	49.1	−3.3	67.0	9.5
Poland	0.0	60.8	7.2	92.4	16.6
Portugal	4.7	26.1	5.9	33.9	11.3
Romania	−2.8	29.6	2.8	38.5	6.4
Slovakia	6.8	58.8	14.1	88.8	20.2
Slovenia	1.9	33.3	6.1	64.9	13.1
Spain	17.9	28.6	20.3	37.7	22.3
Sweden	3.0	32.0	4.8	43.0	12.0
Switzerland	10.5	39.1	10.8	57.0	18.7
UK	6.3	31.0	6.7	42.2	13.2
EU27+2	3.0	29.6	5.4	42.6	11.4

The increase in the male population age over 75 years was more marked than in women in all countries. In men, the EU27+2 population aged 75 years, or more is expected to increase by 43%. The percentage change for people age 50 years or more was greater in men than in women for only three countries (Cyprus, Denmark and Ireland).

**Fig. 17:**
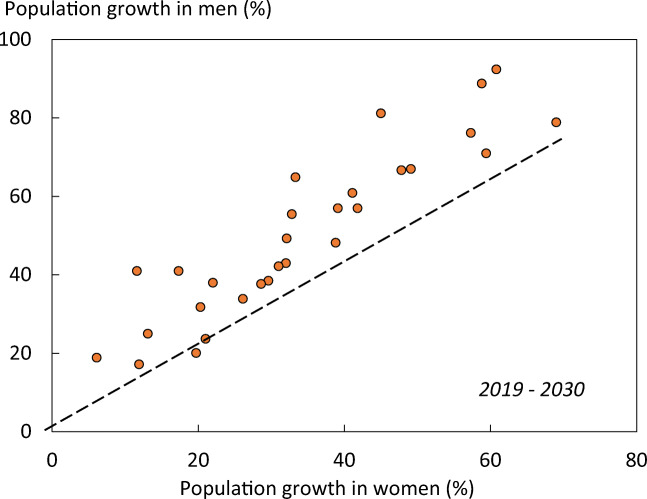
The relation between the percentage increase in the male and female population aged 75 years or more in the EU27+2. The diagonal shows the line of identity

Figure 17 shows the relationship between growth rates of the male and female populations above the age of 74 years. Life expectancy is currently lower in men than women. However, life expectancy is also improving more rapidly in men than women with time, which can be seen in the form of higher growth rates in men for almost all countries in Fig. 17. This trend has further strengthened since the 2010.

#### Score criteria

None—not a score card element

#### Comment

UN population projections over 15 years are relatively robust in that all men and women in 2034 aged 50 years or more had already attained adulthood in 2019. The projections expressed in relative change for countries with very small populations are uncertain (e.g. Malta, Cyprus) since population numbers are given by the UN are rounded to the nearest 1000.

#### References

132. United Nations (UN) (2020) World Population Prospects 2019. UN. Accessed 2020-04-09 2020 https://population.un.org/wpp
133. Stenmark J, McCloskey EV, Jonsson B, Kanis JA (2013) Osteoporosis in the European Union: medical management, epidemiology and economic burden. A report prepared in collaboration with the International Osteoporosis Foundation (IOF) and the European Federation of Pharmaceutical Industry Associations (EFPIA). Arch Osteoporos 8:136
134. Kanis JA, Borgstrom F, Compston J, Dreinhofer K, Nolte E, Jonsson L, Lems WF, McCloskey EV, Rizzoli R, Stenmark J (2013) SCOPE: a scorecard for osteoporosis in Europe. Arch Osteoporos 8:144

### 1i—Fracture projections

#### Domain

Burden of disease—scorecard element

#### Background and aims

As noted previously, the number of men and women age 50 years or more is set to increase with time in the EU27+2. The increase will be particularly marked in the elderly population. Since age is an important risk factor for fractures and the elderly population is projected to increase in the majority of member countries, the burden of fractures is also likely to increase.

The aim of this scorecard element was to estimate the increase in the annual number of fragility fractures from 2019 to 2034 in the EU27+2.

#### Methods

The incidence of hip fracture was determined by updating a systematic literature review [135–137]. For this report, new hip fracture data were available for Bulgaria [138], Croatia [139], Estonia [140], and Switzerland [141]. For other fractures, it was assumed that the age- and sex-specific incidence in relation to hip fractures followed the relationship documented for Sweden [142] and other non-EU countries [143]. Outcomes included the three most common sites of fracture (hip, spine and forearm) as well as other fractures considered to be associated with osteoporosis (i.e. pelvis, rib, humerus, tibia, fibula, clavicle, scapula, sternum, and femoral shaft) [142]. For vertebral fractures, only those coming to clinical attention were included.

Fracture numbers were calculated from age- and sex-specific incidence and population sizes in 5-year age intervals for 2019 and 2034 [144]. It was assumed that the age- and sex-specific incidence of fragility fractures did not change over time.

#### Results

**Fig. 18:**
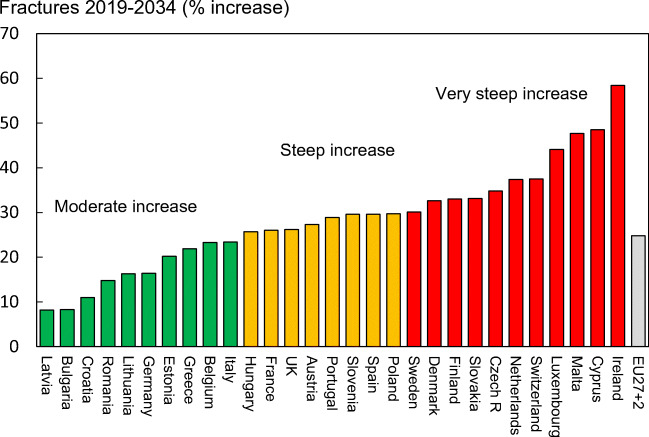
The percentage increase in the number of fragility fractures between 2019 and 2034 in the EU27+2

The annual number of osteoporotic fractures in the EU27+2 will increase by 1.06 million from 4.28 million in 2019 to 5.05 million in 2034 (Table 15). The percentage increase over the 15-year interval varied considerably by country (Fig. 18), ranging from a 58% increase in Ireland to a modest 8% increase in Latvia. In 2034, Germany is projected to have the largest number of annual fractures, with about 931,000 fractures, followed by Italy with about 666,000 fractures.

**Table 15:** Number of fractures in men and women in 2019, the number expected in 2034, and the percentage increase

Country	Number fractures 2019	Number fractures 2034	Difference 2019-2034	Increase in fractures 2019-2034 (%)	Share of EU27+2 increase (%)
Austria	110,196	140,275	30,079	27.3	2.8
Belgium	100,188	123,560	23,372	23.3	2.2
Bulgaria	56,187	60,855	4,668	8.3	0.4
Croatia	34,864	38,716	3,852	11.0	0.4
Cyprus	6,602	9,801	3,199	48.5	0.3
Czech Republic	91,349	123,122	31,773	34.8	3.0
Denmark	86,153	114,232	28,079	32.6	2.6
Estonia	7,870	9,459	1,589	20.2	0.1
Finland	45,254	60,196	14,942	33.0	1.4
France	483,654	609,568	125,914	26.0	11.9
Germany	830,848	966,792	135,945	16.4	12.8
Greece	99,242	120,954	21,712	21.9	2.0
Hungary	86,281	108,489	22,208	25.7	2.1
Ireland	32,367	51,278	18,911	58.4	1.8
Italy	568,424	701,629	133,205	23.4	12.5
Latvia	15,752	17,049	1,297	8.2	0.1
Lithuania	23,148	26,925	3,777	16.3	0.4
Luxembourg	4,053	5,840	1,787	44.1	0.2
Malta	3,232	4,775	1,542	47.7	0.1
Netherlands	99,610	136,821	37,211	37.4	3.5
Poland	205,668	266,653	60,985	29.7	5.7
Portugal	70,730	91,203	20,473	28.9	1.9
Romania	103,035	118,297	15,261	14.8	1.4
Slovakia	75,722	100,785	25,063	33.1	2.4
Slovenia	16,637	21,564	4,926	29.6	0.5
Spain	285,494	369,896	84,402	29.6	7.9
Sweden	123,523	160,647	37,124	30.1	3.5
Switzerland	82,488	113,458	30,970	37.5	2.9
UK	526,974	664,850	137,876	26.2	13.0
EU27+2	4,275,547	5,337,690	1,062,143	24.8	100.0

#### Score criteria

Countries were ranked by the percentage increase in the annual number of fractures in men and women between 2019 and 2034 and colour graded according to the criteria shown in Table 16.

**Table 16:**
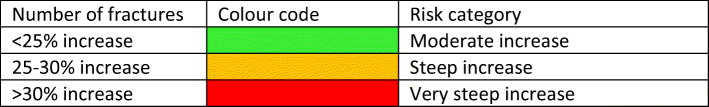
Criteria for allocating scores

#### Score allocation

The percentage increase in the annual number of fractures in men and women between 2019 and 2034 is shown by category and rank in Fig. 18.

#### Comment

The analysis assumes that the age- and sex-specific incidence of fractures did not change over the 15-year time interval. Secular trends in fracture risk are ill-documented with the exception of hip fractures, [145] where limited information is available. In general, age- and sex-adjusted hip fracture incidence increased until the mid or end of the 20th century, with a subsequent plateau or even a small decrease [145]. In Europe, this tendency is best documented for Sweden, Finland, Spain, Germany, Netherlands and Hungary. Countries with substantial increases in the number of fractures need to take this into account for future healthcare planning.

**Fig. 19:**
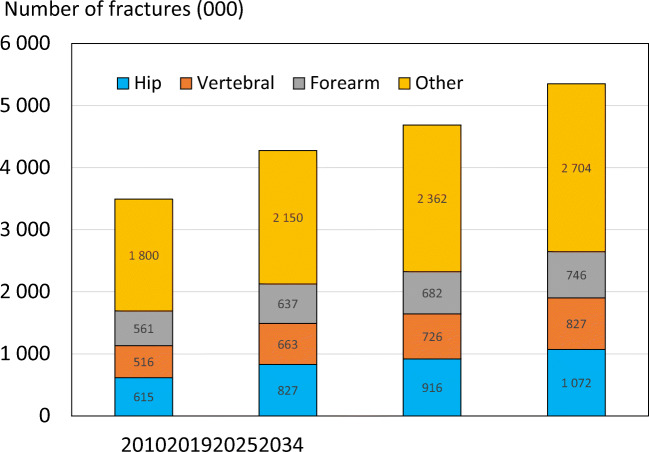
Estimated (2010 and 2019) and predicted number of fractures (2025 and 2034, respectively) in men and women from the EU

It is possible to examine the accuracy of predictions. The number of fractures was estimated for 2010 and again in 2019. At each time, a 15-year prediction was made, providing estimates at year 0 (2010), 9, 15 and 24 years later. As seen in Fig. 19, the 2019 estimate lay between the 2010 estimate and the 2025 prediction at a level consistent with the time intervals at either side (2010-2019 and 2019-2025). This suggests that that the assumptions used to predict fracture numbers are relatively robust.

#### References

135. Hernlund E, Svedbom A, Ivergard M, Compston J, Cooper C, Stenmark J, McCloskey EV, Jonsson B, Kanis JA (2013) Osteoporosis in the European Union: medical management, epidemiology and economic burden. A report prepared in collaboration with the International Osteoporosis Foundation (IOF) and the European Federation of Pharmaceutical Industry Associations (EFPIA). Arch Osteoporos 8:136
136. Kanis JA, Borgstrom F, Compston J, Dreinhofer K, Nolte E, Jonsson L, Lems WF, McCloskey EV, Rizzoli R, Stenmark J (2013) SCOPE: a scorecard for osteoporosis in Europe. Arch Osteoporos 8:144
137. Kanis JA, Oden A, McCloskey EV, Johansson H, Wahl DA, Cooper C (2012) A systematic review of hip fracture incidence and probability of fracture worldwide. Osteoporos Int 23:2239– 2256
138. Kirilova E, Johansson H, Kirilov N, Vladeva S, Petranova T, Kolarov Z, Liu E, Lorentzon M, Vandenput L, Harvey NC, McCloskey E, Kanis JA (2020) Epidemiology of hip fractures in Bulgaria: development of a country-specific FRAX model. Arch Osteoporos 15:28
139. Poljicanin T (2012) Hospital morbidity, Croatian National Institute of Public Health - personal communication. Received by Kanis JA. Hip fracture data from 2012. https://www.sheffield.ac.uk/FRAX/
140. Jurisson M, Vorobjov S, Kallikorm R, Lember M, Uuskula A (2015) The incidence of hip fractures in Estonia, 2005-2012. Osteoporos Int 26:77–84
141. Lippuner K, Johansson H, Kanis JA, Rizzoli R (2009) Remaining lifetime and absolute 10-year probabilities of osteoporotic fracture in Swiss men and women. Osteoporos Int 20:1131–1140
142. Kanis JA, Oden A, Johnell O, Jonsson B, de Laet C, Dawson A (2001) The burden of osteoporotic fractures: a method for setting intervention thresholds. Osteoporos Int 12:417–427
143. Kanis JA, Hans D, Cooper C, Baim S, Bilezikian JP, Binkley N, Cauley JA, Compston JE, Dawson-Hughes B, El-Hajj Fuleihan G, Johansson H, Leslie WD, Lewiecki EM, Luckey M, Oden A, Papapoulos SE, Poiana C, Rizzoli R, Wahl DA, EV MC, Task Force of the FI (2011) Interpretation and use of FRAX in clinical practice. Osteoporos Int 22:2395–2411
144. United Nations (UN) (2020) World Population Prospects 2019. UN. Accessed 2020-04-09 2020. https://population.un.org/wpp/
145. Cooper C, Cole ZA, Holroyd CR, Earl SC, Harvey NC, Dennison EM,Melton LJ, Cummings SR, Kanis JA (2011) Secular trends in the incidence of hip and other osteoporotic fractures. Osteoporos Int 22:1277–1288

## Chapter 2 Policy framework

### 2a—Quality of existing information

#### Domain

Policy framework—scorecard element

#### Background and aims

Fracture incidence is poorly documented in the EU [146, 147]. The fracture type that has been best evaluated is hip fracture. Hip fractures account for the majority of health care expenditure, mortality and morbidity and can be used as a proxy for osteoporosis. The EU comprises countries with some of the highest hip fracture rates worldwide [148], but documentation of the size of the problem and the quality of data varies between countries.

Documentation of the burden of disease is an essential prerequisite to determine the resources that should be allocated to the diagnosis and treatment of the disorder. It also provides information concerning the priority a disease should be awarded by health care policy makers. A fracture registry is a centralised database collecting the number of individual fractures per person, per year, within a population and is used for research and resource allocation. The data collected can also be used to identify high fracture risk patients who might benefit from further prevention programmes.

The main objective of this scorecard element is to provide an integrated estimate of the quality of current documentation on the burden of osteoporosis fractures in the EU 27+2.

#### Methods

Published information on hip fracture incidence was obtained by systematic review, in some cases through contact with Ministries of Health [148] and updated for countries where new publications were available (see *Chapter 1d* for additional information). Available studies in each country were reviewed for quality and representativeness of the country. Epidemiology of other fractures was obtained by systematic review [146].

Data on national or regional fracture registries [149] were collected through an IOF questionnaire sent to the members of the IOF Committee of National Societies in January 2020. The quality of the available information was scored, with the presence of an established national fracture registry as the highest grade. In the absence of a fracture registry, an intermediate score was dependent on the presence of good quality national hip fracture rates.

Where possible, results were compared with the 2010 audit [146, 147].

#### Results

High-quality national data on hip fracture rates were identified in 18 member states (Table 17). Fair- to poor-quality national estimates were found for Slovenia. No fracture data were available for Cyprus and Latvia, while no questionnaire data were received from Luxembourg. In the remaining 7 countries, regional estimates of variable quality were identified. Most index years included data from 2000 onwards.

**Table 17:** Characteristics of information available on fracture rates in the EU27+2

Country	Incidence of hip fracture		Established National Fracture Registries		2010 Score	2019 Score
	Quality^a^	Sample	Present	Data^b^		
Austria	G	National	Yes	Hip+	3	3
Belgium	G	National	No		3	2
Bulgaria	G	Regional	Yes	Hip+	0	3
Croatia	G	National	No		-	2
Cyprus	-	-	No		0	1
Czech Republic	G	National	No		2	2
Denmark	G	National	No		3	2
Estonia	G	National	No		1	2
Finland	G	National	Yes	Hip+	3	3
France	G	National	Yes	Hip	2	3
Germany	G	National	No		2	2
Greece	P/F/G	Regional	No		1	1
Hungary	G	National	Yes	Hip+	3	3
Ireland	G	National	Yes	Hip	3	3
Italy	G	National	Yes	Hip+	2	3
Latvia	-	-	Yes	Hip+	3	3
Lithuania	F	Regional	Yes	Hip+	1	3
Luxembourg	-	-	-		0	-
Malta	G	National	No		2	2
Netherlands	G	National	Yes	Hip	3	3
Poland	F	Regional	No		1	1
Portugal	G	National	No		3	2
Romania	G	National	No		2	2
Slovakia	G	National	Yes	Hip	3	3
Slovenia	F	National	No		2	1
Spain	F/G	Regional	Yes	Hip+	1	3
Sweden	G	National	Yes	Hip+	3	3
Switzerland	G	National	No		-	2
UK	G	Regional	Yes	Hip+	3	3

^a^Quality: *G* good; *F* fair; *P* poor [147];^b^Hip registration of hip fracture only. Hip+ registration of hip and other fracture outcomes

Data on the incidence of clinical vertebral fractures are lacking in most of the countries in the EU, the exceptions being regional data for Sweden and the UK. In the UK, the incidence of clinically identified fractures has been studied within the Clinical Practice Research Datalink (CPRD). The incidence is, however, very low and it is likely that the majority of fractures were not coded [150, 151].

Information on forearm fracture is also scarce. There are reports from EU27+2 countries on the incidence of forearm fractures that lead to hospitalisation, e.g. from France and Italy, but these are of limited value since forearm fractures are treated in hospital outpatient departments. There are also studies published from Slovenia and Italy which present incidence of forearm fractures treated both in inpatient and outpatient care. However, the Slovenian study only reported fractures in women, and the Italian study lacked age stratification of data within the elderly population. Credible data are only available for Austria, Hungary, the UK and Sweden [146, 147, 152, 153].

National fracture registries were in place in 14 of the EU27+2 countries (Table 17). The majority of these collect information on all or several fracture outcomes (Austria, Bulgaria, Finland, Hungary, Italy, Latvia, Lithuania, Spain, Sweden and the UK) and the remainder registered hip fracture alone (France, Ireland, the Netherlands and Slovakia). In several additional countries, local registries are available.

Compared with 2010, there has been some improvement in the capture of information on hip fracture. In the 27 countries where comparative information was available, the number of countries with a score of 2 or 3 rose from 17 to 21.

#### Score criteria

The presence of an established national fracture registry was allocated the highest grade. In the absence of a fracture registry, an intermediate score was given with the availability of good quality national hip fracture rates. Criteria for allocating scores are given in Table 18.

**Table 18:**
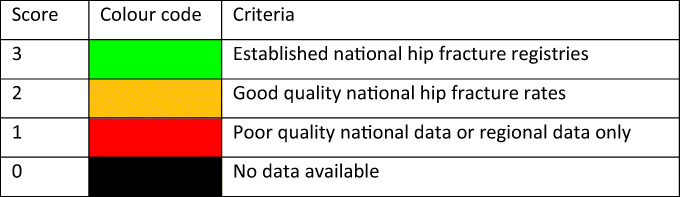
Criteria for allocating scores

#### Score allocation

**Fig. 20:**
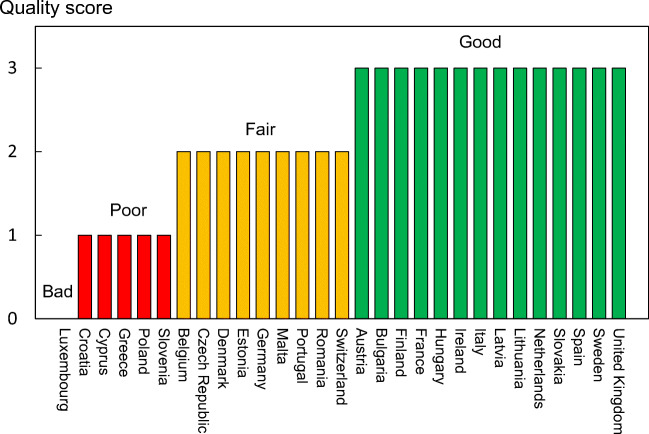
Quality of information available on the epidemiology of hip fractures in the EU27+2 [IOF audit]

Countries, ranked and categorised by score, are shown in Fig. 20.

#### Comment

The quality of this information is limited. Firstly, it is based on responses to a questionnaire to national societies and not to government agencies. Secondly, centralised data are not necessarily equivalent to a national registry.

#### References

146. Hernlund E, Svedbom A, Ivergard M, Compston J, Cooper C, Stenmark J, McCloskey EV, Jonsson B, Kanis JA (2013) Osteoporosis in the European Union: medical management, epidemiology and economic burden. A report prepared in collaboration with the International Osteoporosis Foundation (IOF) and the European Federation of Pharmaceutical Industry Associations (EFPIA). Arch Osteoporos 8:136
147. Kanis JA, Borgstrom F, Compston J, Dreinhofer K, Nolte E, Jonsson L, Lems WF, McCloskey EV, Rizzoli R, Stenmark J (2013) SCOPE: a scorecard for osteoporosis in Europe. Arch Osteoporos 8:144
148. Kanis JA, Oden A, McCloskey EV, Johansson H, Wahl DA, Cooper C (2012) A systematic review of hip fracture incidence and probability of fracture worldwide. Osteoporos Int 23:2239–2256
149. International Osteoporosis Foundation (2010) Osteoporosis in the European Union in 2008. Ten years of progress and ongoing challenges. International Osteoporosis Foundation (IOF). Accessed 2020-07-24 https://www.iofbonehealth.org
150. de Lusignan S, Valentin T, Chan T, Hague N, Wood O, van Vlymen J, Dhoul N (2004) Problems with primary care data quality: osteoporosis as an exemplar. Inform Prim Care 12:147–156
151. Kanis JA, Compston J, Cooper C, Harvey NC, Johansson H, Odén A, McCloskey EV (2016) SIGN Guidelines for Scotland: BMD Versus FRAX Versus QFracture. Calcif Tissue Int 98:417–425
152. Dimai HP, Svedbom A, Fahrleitner-Pammer A, Pieber T, Resch H, Zwettler E, Thaler H, Szivak M, Amrein K, Borgström F (2013) Epidemiology of proximal humeral fractures in Austria between 1989 and 2008. Osteoporos Int 24:2413–2421
153. Dimai HP, Svedbom A, Fahrleitner-Pammer A, Resch H, Muschitz C, Thaler H, Szivak M, Amrein K, Borgström F (2014) Epidemiology of distal forearm fractures in Austria between 1989 and 2010. Osteoporos Int 25:2297–2306

### 2b—National health priority

#### Domain

Policy framework—scorecard element

#### Background and aims

Data from the Global Burden of Disease Study, conducted by the Institute for Health Metrics and Evaluation (IHME), indicates that musculoskeletal disorders in 2017 were the greatest cause of disability as measured by years lived with disability (YLD), worldwide and across most regions of the world [154]. In terms of disability-adjusted life-years (DALY, a measure of health impact that combines both death and disability), musculoskeletal diseases are the non-communicable diseases that have the fifth greatest impact on the health of the world population (5.5% of the world’s DALY loss). They closely follow global DALY loss due to cardiovascular diseases (14.7%), tumours (9.4%), maternal/neonatal disorders (7.9%) and respiratory infections (6.4%) [154]. Disability due to musculoskeletal disorders has increased by 45% from 1990 to 2010 compared to a 33% average across all other disease areas. These data suggest that musculoskeletal disease merits a high priority in healthcare policy.

Osteoporotic fractures in Europe have been estimated to account for more disability-adjusted life years lost (2,006,000 DALYs) than rheumatoid arthritis (1,048,000) but less than that for osteoarthritis (3,088,000) representing 33% of the total DALY loss from these three disorders [155]. A more recent estimate placed the total DALY loss in 2016 related to fragility fractures at more than 2.6 million, which was higher than the estimated DALY loss related to chronic obstructive pulmonary disease and ischaemic stroke, but lower than that for lung cancer or dementia [156].

When a disease becomes a National Health Priority (NHP), it is usually mandated by a government body/ministry of health or another official institution. Osteoporosis may be a designated NHP on its own, or it may be included as part of a musculoskeletal diseases NHP. The development of a national action plan, clear objectives and support for education and awareness programmes also often result from an NHP mandate. The aim of this scorecard element was to determine the extent to which member states have recognised this need.

#### Methods

Information on NHP [157] was updated by an IOF questionnaire to the members of the IOF Committee of National Societies, undertaken in January 2020. Respondents were asked whether osteoporosis or musculoskeletal diseases were officially documented as an NHP in each member state and to provide the documentary evidence. Further questions related to action plans linked to the NHP and their implementation.

Where possible, results were compared with the 2010 audit [158, 159].

#### Results

The majority of countries reporting information on NHPs (19/28) does not recognise osteoporosis or musculoskeletal diseases as an NHP (Table 19). Of those member states that have developed an NHP, the focus has been on nutrition (five countries), exercise (four countries) and falls prevention (four countries). Action plans have been implemented in six countries (Finland, France Italy, Poland, Romania and Sweden). In Sweden, recommendations to health care providers have been issued, but no financial support is given.

**Table 19:** Countries in which osteoporosis or musculoskeletal diseases were officially documented as an NHP, its scope and action plans

Country	NHP and date	Government support	Scope^a^	Action plan	Score 2010	Score 2019
Austria	No				1	1
Belgium	No				1	1
Bulgaria	Yes 2006	Yes		No	3	2
Croatia	No			No		1
Cyprus	No				1	1
Czech Republic	No				1	1
Denmark	No				1	1
Estonia	No				1	1
Finland	Yes 2017	Yes	N, E, F	Yes	2	3
France	Yes 2018	Yes	F, P	Yes	2	3
Germany	Yes 2020	Yes	N, E, F	No	1	2
Greece	No				1	1
Hungary	No		N, E, F, P	No	1	1
Ireland	No				1	1
Italy	Yes 2010	Yes		Yes	2	3
Latvia	No		N, E		1	1
Lithuania	No				1	1
Luxembourg	-				3	0
Malta	No				1	1
Netherlands	No				1	1
Poland	Yes	Yes	N	Yes	1	3
Portugal	No				2	1
Romania	Yes 2005	Yes		Yes	3	3
Slovakia	No				1	1
Slovenia	No				1	1
Spain	Yes	Yes		No	1	2
Sweden	Yes 2012	Yes	F, P	Yes	2	3
Switzerland	No					1
UK	No				3	1

^a^*N*, nutrition; *E*, exercise; *F*, falls prevention; *P*, professional education

#### Score criteria

The presence of government-backed NHP with an implemented action plan was allocated the highest grade. In the absence of an action plan, an intermediate score was given. Criteria for allocating scores are given in Table 20.

**Table 20:**
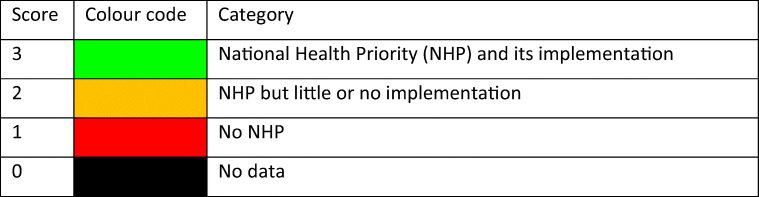
Criteria for allocating scores

#### Score allocation

**Fig. 21:**
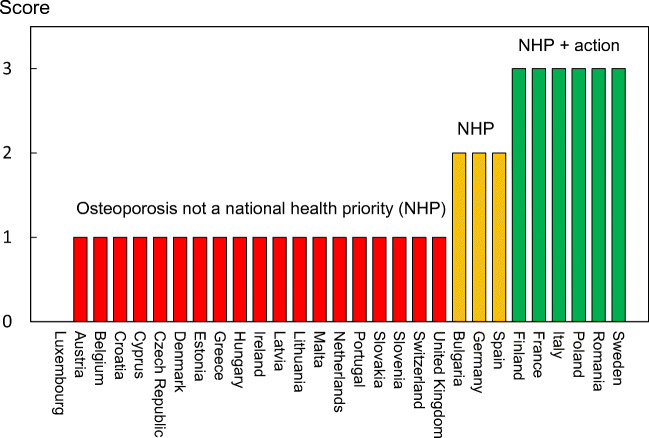
Categorisation of EU27+2 countries according to the existence of government-backed NHP for osteoporosis or musculoskeletal diseases [IOF audit]

Countries, ranked and categorised by score, are shown in Fig. 21. The scores from the original SCOPE study are provided for comparison.

#### Comment

Unless osteoporosis prevention and treatment become a priority for governments and health care providers, the growing number of osteoporotic fractures will have a serious impact on society—not just in terms of people’s health-related quality of life, but also because of increased costs incurred for acute health care, rehabilitation and nursing care.

#### References

154. Institute for Health Metrics and Evaluation (2017) Global Burden of Disease 2017 (GBD Compare - Viz Hub). Accessed 2020-06-18 https://vizhub.healthdata.org/gbd-compare/
155. Johnell O, Kanis JA (2006) An estimate of the worldwide prevalence and disability associated with osteoporotic fractures. Osteoporos Int 17:1726–1733
156. Borgstrom F, Karlsson L, Ortsater G, Norton N, Halbout P, Cooper C, Lorentzon M, McCloskey EV, Harvey NC, Javaid MK, Kanis JA (2020) Fragility fractures in Europe: burden, management and opportunities. Arch Osteoporos 15:59
157. International Osteoporosis Foundation (2010) Osteoporosis in the European Union in 2008. Ten years of progress and ongoing challenges. International Osteoporosis Foundation (IOF). Accessed 2020-07-24 www.iofbonehealth.org
158. Hernlund E, Svedbom A, Ivergard M, Compston J, Cooper C, Stenmark J, McCloskey EV, Jonsson B, Kanis JA (2013) Osteoporosis in the European Union: medical management, epidemiology and economic burden. A report prepared in collaboration with the International Osteoporosis Foundation (IOF) and the European Federation of Pharmaceutical Industry Associations (EFPIA). Arch Osteoporos 8:136
159. Kanis JA, Borgstrom F, Compston J, Dreinhofer K, Nolte E, Jonsson L, Lems WF, McCloskey EV, Rizzoli R, Stenmark J (2013) SCOPE: a scorecard for osteoporosis in Europe. Arch Osteoporos 8:144

### 2c—Who manages osteoporosis?

#### Domain

Policy framework—scorecard element

#### Background and aims

In 2019, 25.5 million women and 6.5 million men in the EU27+2 were estimated to have osteoporosis using the diagnostic criterion of the WHO (*Chapter 1c* ) and the number of new osteoporosis-related fractures was 4.3 million (*Chapter 1e)*. Given that osteoporosis and fragility fractures are common and that effective treatments are widely available, the vast majority of patients with osteoporosis are preferably managed at the primary health care level by general practitioners (GPs), with specialist referral reserved for complex cases. Examples of this would be men and individuals in whom a secondary cause of osteoporosis is suspected.

The aim of this element was to determine whether the care of osteoporosis was primarily devolved to primary care physicians (GPs, family doctors). If not, then the lead specialty was requested. The training of specialists is considered in *Chapter 2d*.

#### Methods

Data were acquired by an IOF questionnaire to the members of the IOF Committee of National Societies, undertaken in January 2020. Respondents were asked whether osteoporosis was primarily devolved to primary care physicians (GPs, family doctors). If not, the single specialty that looked after most cases of osteoporosis was ascertained. In the case where there was near equality between two or more specialties, they were each recorded. Where possible, results were compared with the 2010 audit [160, 161].

#### Results

Primary care was the principal provider of the medical care for osteoporosis in 16 of the 28 countries with questionnaire responses (Table 21). In the remainder, the care was provided principally by hospital specialists. In 8 of the remaining countries, a single hospital specialty was the dominant provider (mainly rheumatology or endocrinology). In the remaining countries, the care of osteoporosis was split between disciplines. The number of disciplines was two (for Czech Republic and Hungary), or more in the case of Poland and Switzerland. The specialties involved comprised endocrinology (noted 8 times), rheumatology (7), geriatrics (2), internal medicine (2), osteology (1), gynaecology (1) and orthopaedics (1). The panel were concerned by the multiplicity of specialists that had a primary role in the care pathway of patients in some countries and viewed this as an impediment to consistent care. No response to the questionnaire was received for Luxembourg.

**Table 21:** Care pathway for patients with osteoporosis, by country

Country	Primarily devolved to primary care	Lead specialty	Score 2010	Score 2019
Austria	Yes		3	3
Belgium	Yes		3	3
Bulgaria	No	Rheumatology	1	2
Croatia	No	Endocrinology		2
Cyprus	No	Endocrinology	3	2
Czech Republic	No	Osteology Rheumatology Endocrinology, Internal medicine	1	1
Denmark	Yes		1	3
Estonia	Yes		3	3
Finland	Yes		3	3
France	Yes		3	3
Germany	Yes			3
Greece	No	Orthopaedic surgery	2	2
Hungary	No	Rheumatology Endocrinology	2	1
Ireland	Yes		1	3
Italy	Yes		1	3
Latvia	Yes		3	3
Lithuania	Yes		3	3
Luxembourg	-		3	0
Malta	No	Rheumatology	1	2
Netherlands	No	Endocrinology	3	2
Poland	No	Rheumatology Endocrinology Geriatrics	1	1
Portugal	Yes		3	3
Romania	No	Endocrinology	1	2
Slovakia	No	Rheumatology	1	2
Slovenia	Yes		3	3
Spain	Yes		3	3
Sweden	Yes		3	3
Switzerland	No	Rheumatology Gynaecology Endocrinology Geriatrics Internal medicine		1
UK	Yes		3	3

Since the original SCOPE study, six countries have worsened their scores while seven countries have improved their scores.

#### Score criteria

Where the care of osteoporosis was primarily devolved to primary care physicians (GPs, family doctors), this was allocated the highest grade. If not, then an intermediate score was given where osteoporosis is mainly managed by a single specialty, as given in Table 22.

**Table 22:**
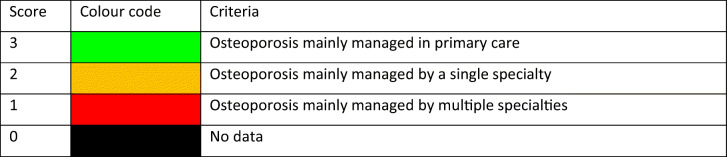
Criteria for allocating scores

#### Score allocation

**Fig. 22:**
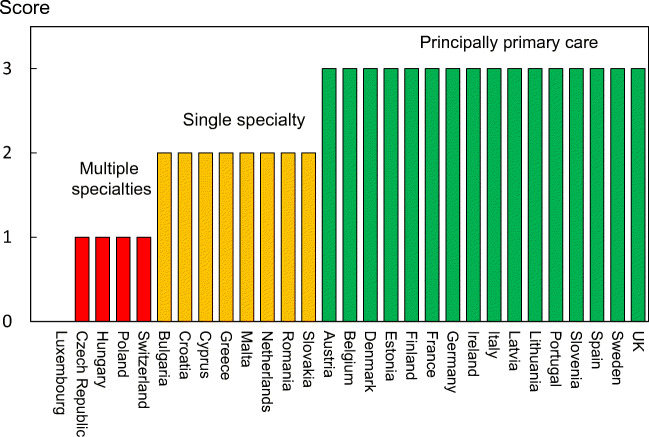
Patterns of principal care of patients with osteoporosis [IOF audit]

Countries, ranked and categorised by score, are shown in Fig. 22.

#### Comment

Care management pathways are not necessarily divided by primary care and specialty care. The panel supports the view that long-term management should preferably be undertaken by GPs, contingent on adequate training, but there is a specialist role in initial evaluation, particularly in the context of fracture liaison services (see *Chapter 3 g*). In Germany, at the time of the original SCOPE study, there was the opportunity for specialists in many disciplines to be specially trained and accredited in the primary care of patients with osteoporosis. These considerations should temper the interpretation of the scores allocated. Additionally, no consideration is given to delivery of osteoporosis care by non-physician healthcare professionals such as nurse practitioners.

#### References

160. Hernlund E, Svedbom A, Ivergard M, Compston J, Cooper C, Stenmark J, McCloskey EV, Jonsson B, Kanis JA (2013) Osteoporosis in the European Union: medical management, epidemiology and economic burden. A report prepared in collaboration with the International Osteoporosis Foundation (IOF) and the European Federation of Pharmaceutical Industry Associations (EFPIA). Arch Osteoporos 8:136
161. Kanis JA, Borgstrom F, Compston J, Dreinhofer K, Nolte E, Jonsson L, Lems WF, McCloskey EV, Rizzoli R, Stenmark J (2013) SCOPE: a scorecard for osteoporosis in Europe. Arch Osteoporos 8:144

### 2d—Is osteoporosis a component of specialty training?

#### Domain

Policy framework—scorecard element

#### Background and aims

The large number of men and women who suffer the consequences of osteoporosis raises the question of whether there is adequate training of medical practitioners in this specialty and, indeed, which specialty takes a leadership role.

The aim of this background element was to determine whether osteoporosis and metabolic bone disease are a recognised specialty or recognised component of specialty training.

#### Methods

Data were acquired by an IOF questionnaire to the members of the IOF Committee of National Societies, undertaken in January 2020. The requested information included whether osteoporosis or metabolic bone disease is a recognised medical specialty in each country. In addition, participating organizations were asked whether osteoporosis or metabolic bone disease is a recognised component of specialty medical training and, finally, which specialists took lead roles in the care of osteoporosis.

The available information was scored, with the presence of an established specialty as the highest grade. In the absence of osteoporosis or metabolic bone disease being a recognised medical specialty, an intermediate score was dependent on the disorder being a recognised component of specialty medical training.

#### Results

Osteoporosis and metabolic bone disease are recognised specialties in only four of the EU27+2 countries (Czech Republic, Ireland, Lithuania and Slovakia). In some countries, there are specialists that deal exclusively with metabolic bone diseases (e.g. the UK) most usually in an academic setting. The more usual finding is that the specialty care of osteoporosis is via another specialty (Table 23). The specialties involved include endocrinology, geriatrics, gynaecology, internal medicine, orthopaedic surgery, rehabilitation medicine and rheumatology, osteology, primary care, traumatology and neurology. In the majority of countries, osteoporosis or metabolic bone disease is a recognised component of specialty medical training but there is no information on the extent to which this is taken advantage of.

**Table 23:** Specialists caring for osteoporosis (OP)

Country	OP recognized as a specialty	Principal specialties	OP recognized as a component of specialty training	Score
Austria	No	Endo, Rh, Gyn, Orth	Yes	2
Belgium	No	Rehab	Yes	2
Bulgaria	No	Int, Orth, Endo, Rh	Yes	2
Croatia	No	Endo, Rehab, Orth, Gyn	Yes	2
Cyprus	No	Endo,	No	1
Czech Republic	Yes	Int, Orth, Gyn, Endo, Rh, Ost	Yes	3
Denmark	No	Endo	Yes	2
Estonia	No	Orth, Gyn, Endo, Rh	Yes	2
Finland	No	Endo, Int, Ger, Orth, Gyn	Yes	2
France	No	Rh, Ger, Endo, Prim, Gyn, Orth, Int	Yes	2
Germany	No	Ost, Orth, Gyn, Rh, Endo	Yes	2
Greece	No	Orth, Endo, Rh	Yes	2
Hungary	No	Endo, Rh	Yes	2
Ireland	Yes	Ger, Rh, Orth	Yes	3
Italy	No	Rh, Endo, Int, Rehab, Orth	Yes	2
Latvia	No		No	1
Lithuania	Yes	Int, Ger, Endo, Rh, Orth, Prim	Yes	3
Luxembourg	-			0
Malta	No	Orth, Gyn, Rehab, Rh, Endo, Ger	Yes	2
Netherlands	No	Int, Orth, Gyn, Endo, Rh, Trau	Yes	2
Poland	No	Int, Orth, Rh, Endo, Ger, Gyn	Yes	2
Portugal	No	Int, Orth, Gyn, Endo, Rh, Prim, Rehab, Neuro	Yes	2
Romania	No	Endo, Rh, Rehab	Yes	2
Slovakia	Yes	Orth, Endo, Rh	Yes^b^	3
Slovenia	No	Prim, Int, Orth, Gyn, Endo	Yes	2
Spain	No	Int, Orth, Gyn, Endo, Rh	Yes	2
Sweden	No	Orth, Endo, Ger, Prim	Yes	2
Switzerland	No	Endo, Rh, gyn, Ger, Int	Yes	2
UK	No	Rh, Endo	Yes	2

^a^*Endo* endocrinology; *Ger* geriatrics; *Gyn* gynaecology; *Int* internal medicine; *Orth* orthopaedic surgery; *Prim* primary care; *Rehab* rehabilitation medicine; *Rh* rheumatology, *Traum* traumatology

^b^Certification possible after specialisation

In two countries (Cyprus and Latvia), osteoporosis was neither an accepted medical specialty nor a component of specialty medical training. In the UK, experience in metabolic bone disease may form a component of specialist training but is not mandatory.

**Fig. 23:**
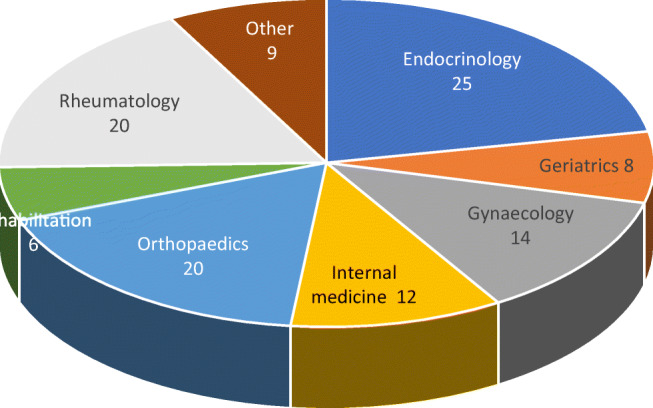
The specialty representation in the EU27+2 countries. Note that more than one specialty per country can be represented (see Table 23) [IOF audit]. Other comprised osteology, primary care, traumatology, and neurosurgery

With the exceptions of Belgium and Denmark, the lead specialties are multiple. In twelve countries, five or more specialties took what were considered lead roles in the management of osteoporosis. This clearly indicates that there is no dominant specialty that looks after osteoporosis in any one country and a great diversity between countries. The specialty representation is illustrated in Fig. 23.

#### Score criteria

The highest score was allocated to a country if osteoporosis or metabolic bone disease was an established specialty. In the absence of osteoporosis or metabolic bone disease being a recognised medical specialty, an intermediate score was dependent on the disorder being a recognised component of specialty medical training (Table 24).

**Table 24:**
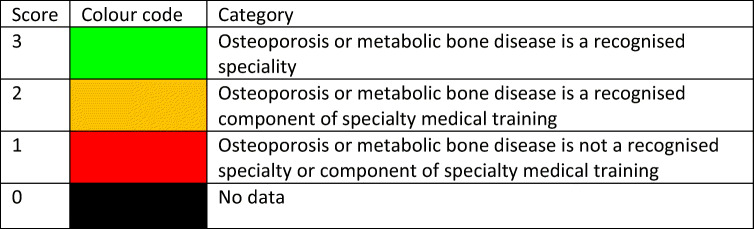
Criteria for allocating scores

#### Score allocation

**Fig. 24:**
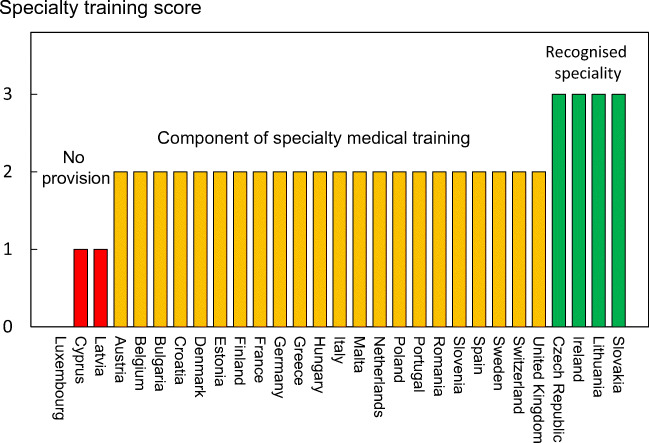
The score allocation and grade for specialist training in each country [IOF audit]

The score allocation and grade for each country is shown in Fig. 24.

#### Comment

There is a wide variation in the specialties which cater to osteoporosis. Although it is possible that these specialties educate their trainees adequately, the wide variation may reflect inconsistencies in patient care, training of primary care physicians and a suboptimal voice to ‘defend’ the interests of those who work within the field of osteoporosis.

### 2e—Patient organisations

#### Domain

Policy framework—scorecard element

#### Background and aims

The role of national patient organisations is to improve the care of patients and increase awareness and prevention of osteoporosis and related fractures among the general public. In addition to their role in patient and public outreach, the organisations provide practical information for osteoporosis patients and their families through telephone help lines, local self-help groups and information events, media outreach, general educational activities and by distributing information via brochures and their websites. The patient organisations often work closely with clinical and research associations to disseminate information about new treatments and patient guidelines.

Finally, with their often large and active membership base, organisations play an important role in advocacy by calling for access to timely and affordable diagnosis and treatment. This is particularly necessary for osteoporosis which, as a chronic ‘silent’ disease, is too often neglected by health authorities.

#### Methods

Data on the patient organisations operating in the EU27+2 countries were acquired by an IOF questionnaire to the members of the IOF Committee of National Societies, undertaken in January 2020. According to a report by the European Patients Forum about the added value of patient organisations, advocacy by patient organisations can fall into four categories [162]:

- Policy: Work on promoting the interest of patient during all stages of policy development
- Capacity building and education: Invest in capacity building and educational initiatives for policy makers, industry, academia and media
- Peer support: Provide peer mentoring and counselling services and legal and financial support to patients
- Research and Development: Active research collaborators

Priority was placed on those countries with patient organisations that covered all four advocacy areas, and in its absence, countries that had any form of patient organisation for osteoporosis.

#### Results

The individual organisations are listed in the acknowledgements. The existence of patient organisations, and their respective advocacy areas, is shown by country in Table 25. Twenty six out of 28 responding countries knew of at least one patient organisation. Five of the responders (Austria, Croatia, Germany, Portugal and Spain) listed more than one organisation. Of the countries with organisations, 10 countries’ organisations covered all four of the advocacy areas. Only Switzerland responded with support in one advocacy area.

**Table 25:** The existence of patient organisations in each of the EU27+2, and their areas of patient advocacy [IOF audit]

Country	Presence of patient organisations	Patient advocacy areas				Score 2019
		Policy	Capacity	Peer support	Research and development	
Austria	Yes	x	x	x	x	3
Belgium	Yes					2
Bulgaria	Yes					2
Croatia	Yes	x	x	x		2
Cyprus	Yes	x	x			2
Czech Republic	Yes			x		2
Denmark	Yes	x	x		x	2
Estonia	No					1
Finland	Yes	x	x	x	x	3
France	Yes	x	x	x	x	3
Germany	Yes	x	x	x		2
Greece	Yes	x	x		x	2
Hungary	Yes					2
Ireland	Yes	x	x	x	x	3
Italy	Yes	x	x	x	x	3
Latvia	Yes	x	x	x	x	3
Lithuania	Yes	x	x	x	x	3
Luxembourg						0
Malta	Yes	x	x	x		2
Netherlands	Yes	x	x	x		2
Poland	No					1
Portugal	Yes	x	x	x	x	3
Romania	Yes	x	x	x	x	3
Slovakia	Yes	x	x	x		2
Slovenia	Yes	x	x	x		2
Spain	Yes	x	x		x	2
Sweden	Yes	x	x	x		2
Switzerland	Yes			x		2
UK	Yes	x	x	x	x	3

#### Score criteria

Patient support from these organisations was categorised by advocacy areas (Table 26). A high score was allocated to those countries with patient organisations that covered all four advocacy areas. In its absence, an intermediate grade was allocated to countries with any patient organisations and the lowest score to countries with no patient outreach.

**Table 26:**
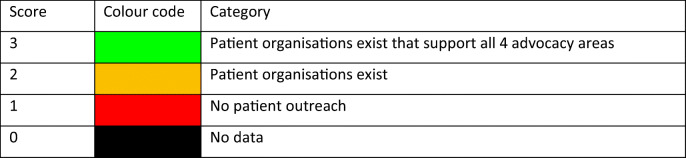
Criteria for allocating scores

#### Score allocation

**Fig. 25:**
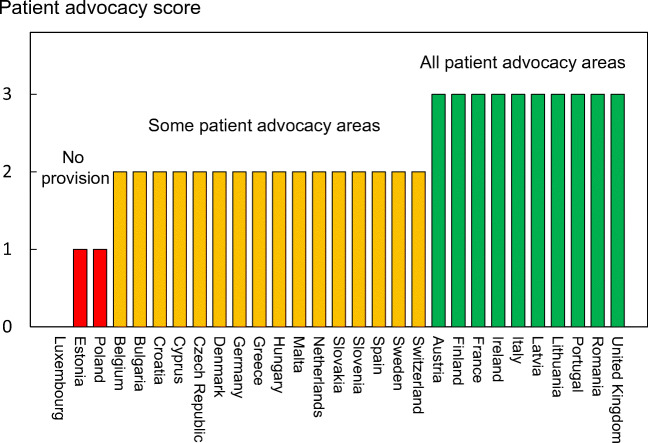
Scores for patient-organisation support, by country [IOF audit]

The score for each country by score and rank is shown in Fig. 25.

#### Comment

The score is based on the audit by the IOF of its affiliated organisations. As such, it necessarily did not consider organisations that are not members of the IOF Committee of National Societies. This consideration should temper the interpretation of this element.

#### Reference

162. Sienkiewicz D, van Lingen C (2017) The Added Value of Patient Organisations: European Patients Forum. Belgium. https://www.eu-patient.eu/globalassets/library/publications/epf_added_value_report_final.pdf

## Chapter 3 Service provision

### 3a—Treatments for osteoporosis

#### Domain

Service provision—scorecard element

#### Background and aims

A wide variety of approved drug treatments is available for the management of osteoporosis. Potential limitations of their use in member states relate to reimbursement policies which may impair the delivery of health care. The aim of this scorecard element was to review the provision of medical intervention in each of the 29 states (27 EU countries, as well as Switzerland and the UK) and, in particular, to determine whether restricted reimbursement was considered an obstacle to the accessibility and long-term uptake of interventions. An ancillary aim was to assess any changes in reimbursement of treatments for osteoporosis since 2010.

#### Methods

Information on access to treatment [163, 164] was updated by an IOF questionnaire to the members of the IOF Committee of National Societies undertaken in January 2020. Information requested included the treatments that are currently reimbursed, the level of reimbursement, the conditions on which reimbursement are offered and whether reimbursement policy interferes with what patients could accept or physicians in each country would wish to recommend to patients. We additionally asked whether there are designated first-line treatments in each country. First-line treatment refers to the first method that a doctor or a reimbursement agency chooses to treat a particular illness, in this case osteoporosis. Alternative terms are induction therapy, primary therapy, and primary treatment. Second-line treatment is treatment after the initial treatment (first-line treatment) has failed, stopped working, or has side effects that aren't tolerated.

The following interventions were included: bisphosphonates (alendronate, clodronate, ibandronate, pamidronate, risedronate and zoledronate), raloxifene, denosumab, strontium ranelate, teriparatide and vitamin D analogues (alphacalcidol, calcidiol and calcitriol). Gonadal steroids (prescribed for hypogonadal states rather than for osteoporosis) were excluded as were calcium/vitamin D products (most usually available without prescription). Costs for treatment per year were based on market data. Costs for first-line treatment and average total osteoporosis treatment costs per year (weighted on price and market share in each country) were estimated based on sales data from IQVIA.

The available information was scored based on full or partial reimbursement. In those countries with restricted reimbursement, countries were identified where reimbursement policy interfered with what patients could accept or physicians would wish to recommend to patients. Countries where there had been significant changes in reimbursement levels or drugs qualifying for reimbursement were highlighted.

#### Results

Most interventions were reimbursed in most countries. Full reimbursement was provided in 11 of 25 member states (Table 27). In the remaining countries, the level of reimbursement ranged from 30% (Poland) to up to 100% for selected treatments (Czech Republic, Estonia, Romania). Restricted reimbursement was reported as a significant obstacle to accessibility and long-term uptake in several countries. Examples include age restrictions for some agents (Czech Republic, Poland), no reimbursement for some agents in the absence of a second prior fracture (Croatia, France, Germany), and reimbursement for some or all agents, conditional on a specialist referral (Belgium, Croatia, Czech Republic, Denmark, Greece, Hungary, Italy, Lithuania, Sweden and UK). It was also reported in the UK that treatments based on clinical efficacy are not always possible due to restrictions imposed by decisions on cost effectiveness thresholds [IOF audit 2020].

**Table 27:** Levels of reimbursement, reported barriers to care from reimbursement policies and costs of treatment [IOF audit]. Reimbursement levels denote the ranges reported for treatments that are reimbursed in each country (i.e. treatments not reported in Table 28). Reimbursement ranges may vary dependent on drugs, medical indication or specialist prescription

Country	Reimbursed 2019 (%)	Score 2012	Score 2019	Patient or professional impediment	First-line drugs identified	Average cost (€/year)	Generic alendronate (€/year)
Austria	100	3	3	No	Yes	230	124
Belgium	61-98	1	1	Yes	Yes	193	65
Bulgaria	50	1	2	No	Yes	235	163
Croatia	100	nr	3	Yes	Yes	187	99
Cyprus	100	3	3	Yes	Yes	-	-
Czech Republic	90-100	2	1	Yes	Yes	161	66
Denmark	100	2	3	No	Yes	173	49
Estonia	50-100	1	2	No	No	133	84
Finland	40	2	2	No	No	226	75
France	65	1	2	Yes	Yes	225	100
Germany	100	3	3	Yes	Yes	278	91
Greece	75	1	1	Yes	Yes	239	101
Hungary	70-90	1	2	No	Yes	125	16
Ireland	*	3	2	No	No	285	79
Italy	100	3	3	Yes	Yes	231	118
Latvia	50	0	2	No	Yes	103	125
Lithuania	100	1	3	No	Yes	142	70
Luxembourg	nr	2	0			249	67
Malta	nr	2	0	No	No	-	-
Netherlands	100	3	3	No	No	84	19
Poland	30	1	2	No	Yes	47	23
Portugal	69	2	2	No	Yes	133	101
Romania	50-100	1	2	No	Yes	102	58
Slovakia	90	2	2	No	Yes	194	
Slovenia	100	3	3	Yes	Yes	233	130
Spain	90	1	2	No	No	329	101
Sweden	100	3	3	No	Yes	120	16
Switzerland	100	nr	3	No	Yes	374	217
UK	100	3	3	Yes	Yes	60	15

*nr*, no return

*Level of reimbursement is means tested

In several countries, reimbursement was conditional on clinical criteria, which prevented health care professionals from prescribing some or all agents to individuals at high risk. Examples include reimbursement criteria based on BMD alone (i.e. irrespective of prior fractures in cases of osteopenia; Sweden) or on age alone (Ireland). In several countries (e.g. Denmark, Sweden) full reimbursement is given above a certain out-of-pocket cost per year. In France, the intricacies of reimbursement for BMD and second line treatment are considered too complicated by GPs.

First-line drugs were mandated in 22 of 28 countries. The majority comprised the oral bisphosphonates and in particular generic alendronate.

The average cost of intervention (weighted on price and market share in each country) varied markedly and ranged from € 47 (Poland) to € 374 (Switzerland). There was similar price inequality for generic alendronate (Table 27). In several countries, some registered treatments were not reimbursed, and these are listed in Table 28.

**Table 28:** Registered treatments that are not reimbursed

Treatment	Countries where reimbursement is not offered for osteoporosis*
Risedronate	Malta
Alendronate	Malta, Slovakia
Ibandronate	Cyprus, Malta
Zoledronate	Bulgaria, Ireland, Malta, Poland
Raloxifene	Czech Republic, Estonia, Finland, Hungary, Latvia, Lithuania, Malta, Poland
Denosumab	Cyprus, Ireland, Malta
Strontium Ranelate	Only markets with reimbursement: Cyprus, Italy, Lithuania, the Netherlands, Romania, Spain, UK [removed from several markets]
Teriparatide and PTH	Estonia, Ireland, Latvia, Malta, Poland, Romania
Alfacalcidol/calcitriol/calcidiol	Finland, Ireland, Lithuania, Malta, Poland, Romania

*Data for Luxembourg not reported

The proportion of member states offering full reimbursement went up from 27 to 44% compared to the last IOF audit. Note however that full reimbursement does not necessarily denote full access to treatment. For example, in Germany and the UK, the availability of drugs other than generic alendronate is restricted by regional or local budgetary policies.

#### Score criteria

The highest score was allocated for full reimbursement. In those countries with restricted reimbursement, countries were identified where reimbursement policy interfered with what patients could accept or physicians would wish to recommend to patients. Categories are shown in Table 29.

**Table 29:**
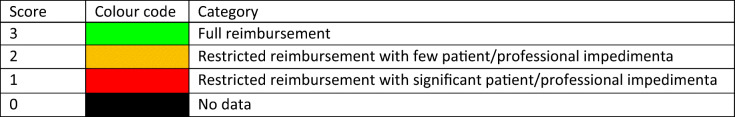
Criteria for allocating scores

#### Score allocation

**Fig. 26:**
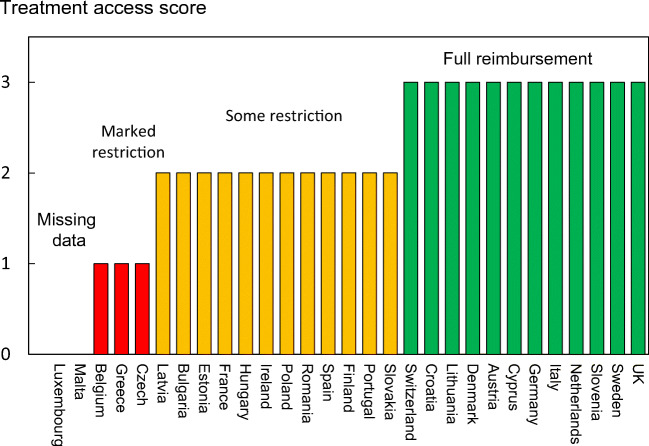
Ranking and score for access to medical intervention [IOF audit]

Countries, ranked and categorised by score, are shown in Fig. 26.

#### Comment

The large price range of generic alendronate between the member states (€15 to €217 per year) is remarkable as an index of inequality of provision amongst the member states. The differences between member states have decreased somewhat since last IOF audit in 2012 [IQVIA sales data] but are still significant.

#### References

163. International Osteoporosis Foundation (2010) Osteoporosis in the European Union in 2008. Ten years of progress and ongoing challenges. International Osteoporosis Foundation (IOF). Accessed 2020-07-24 www.iofbonehealth.org
164. Kanis JA, Borgstrom F, Compston J, Dreinhofer K, Nolte E, Jonsson L, Lems WF, McCloskey EV, Rizzoli R, Stenmark J (2013) SCOPE: a scorecard for osteoporosis in Europe. Arch Osteoporos 8:144

### 3b—Availability of DXA

#### Domain

Service provision—scorecard element

#### Background and aims

The assessment of bone mineral density forms a cornerstone for the general management of osteoporosis, being used for diagnosis, risk prediction, selection of patients for treatment and monitoring of patients on treatment. The appropriate sites and technology are measurement at the lumbar spine and hip with dual-energy x-ray absorptiometry (DXA). DXA can also be used for vertebral fracture assessment (VFA) from lateral scans [165]. The capacity to service these needs depends therefore on the availability of equipment. The assessment of trabecular bone score (TBS), which measures an aspect of bone quality, is a more recent methodology available on many bone densitometers [166].

The aim of this score card element was to compare the availability of DXA in the 29 member states. Ancillary aims were to compare the availability of TBS and the change in availability of DXA since 2010.

#### Methods

An estimate of the number of operational DXA machines was determined from the combined sales information of the three major providers (GE Lunar, Hologic and Norland) provided in confidence for the IOF [167]. The metric for each country was the number of DXA units/million of the general population. Data for TBS were obtained from the same source. Comparable data for VFA are not currently available.

Where paired data were available, comparison was made with data for 2010 [168].

#### Results

**Fig. 27:**
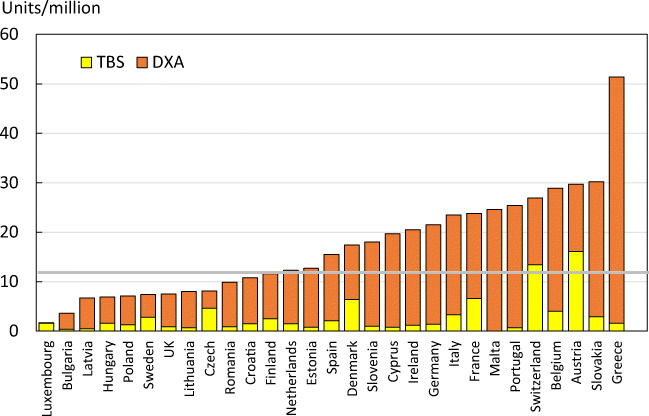
DXA units/million of the general population in 2019 based on sales of DXA supplied by manufacturers. The number of DXA units providing TBS is shown in the lower of the stacked bars. The horizontal line denotes a minimum service requirement for DXA [165]

The number of DXA units expressed per million of the general population varies markedly (Table 30). Greece, France and Austria are the most well provided for and Luxembourg, Serbia and Bulgaria, the least. Previous surveys have indicated a marked heterogeneity in the availability of DXA in the member states [168–171] and the present survey, based on manufacturer sales, confirms this finding (Fig. 27).

**Table 30:** The number of central DXA units available per million of the general population in 2019 [167]

Country	DXA units/million	Country	DXA units/million	Country	DXA units/million
Austria	29.7	Germany	21.5	Netherlands	12.3
Belgium	28.9	Greece	51.4	Poland	7.1
Bulgaria	3.6	Hungary	6.9	Portugal	25.4
Croatia	10.8	Ireland	20.5	Romania	9.9
Cyprus	19.7	Italy	23.5	Slovakia	30.2
Czech Republic	8.1	Latvia	6.7	Slovenia	18.0
Denmark	17.4	Lithuania	8.0	Spain	15.5
Estonia	12.7	Luxemburg	1.7	Sweden	7.4
Finland	11.6	Malta	24.6	Switzerland	26.9
France	23.8			UK	7.5

The highest availability of TBS was in Austria, Switzerland. Denmark and France (16.1, 13.4, 6.8, 6.6/million, respectively). TBS was not available in Iceland or Malta. The uptake of TBS is dependent on the availability of DXA. The proportion of DXA units providing TBS was 30% or more in Austria, Czech, Denmark, Luxembourg, Serbia, Sweden and Switzerland (Fig. 27).

#### Score criteria

The score was based on the number of DXA units/million of the general population categorised as given in Table 31.

**Table 31:**
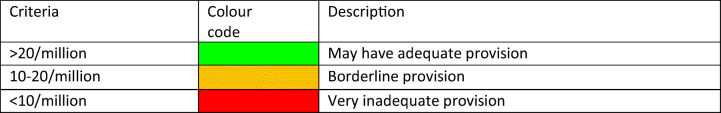
Criteria for allocating scores

#### Score allocation

**Fig. 28:**
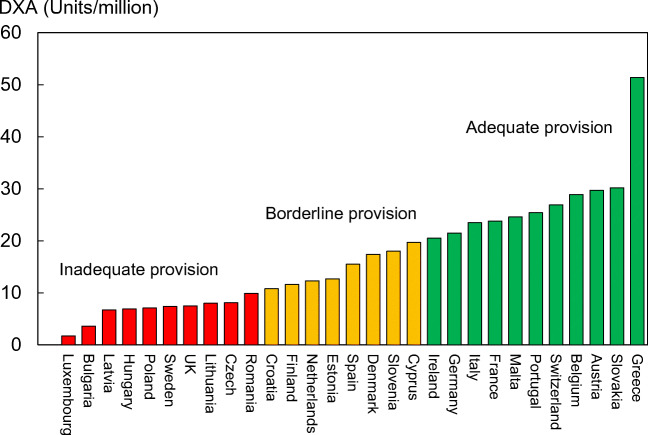
DXA units/million of the general population in 2019 based on sales of DXA supplied by manufacturers

Countries, ranked and categorised by score, are shown in Fig. 28.

Improvement in category of DXA availability since 2010 was seen for Ireland, Slovakia, Malta, Sweden and Spain. For Estonia and Slovenia there was a downward change in category. There were no comparative data available for Croatia, Iceland, Serbia or Switzerland. Where comparative data were available, DXA provision increased on average from 15.5/million of the general population in 2010 to 16.3/million in 2019.

#### Comment

The requirement for assessing and monitoring the treatment of osteoporosis to implement practice guidelines has been estimated at approximately 11 DXA units per million of the general population [168]. The survey indicated that about 60% of member states had the minimum recommended number of DXA machines for their population. It is important to note that the figures provided do not distinguish between machines dedicated in part or in full to clinical research, and those that lie idle or are underutilised because of lack of funding. The granularity of the data means that it is also not possible to determine the extent of inequity in the geographic distribution of DXA machines within countries. It is likely, therefore, that a majority of countries are under-resourced in the context of their practice guidelines. The increase in DXA equipment between 2010 and 2019 is trivial (5%) when placed against the rise in the number of fragility fractures over the same interval (+17% for the EU27, i.e. not including Croatia or Switzerland).

#### References

165. Yang J, Mao Y, Nieves JW (2020) Identification of prevalent vertebral fractures using Vertebral Fracture Assessment (VFA) in asymptomatic postmenopausal women: A systematic review and meta-analysis. Bone 136:115358
166. McCloskey EV, Odén A, Harvey NC, Leslie WD, Hans D, Johansson H, Barkmann R, Boutroy S, Brown J, Chapurlat R, Elders PJM, Fujita Y, Glüer CC, Goltzman D, Iki M, Karlsson M, Kindmark A, Kotowicz M, Kurumatani N, Kwok T, Lamy O, Leung J, Lippuner K, Ljunggren Ö, Lorentzon M, Mellström D, Merlijn T, Oei L, Ohlsson C, Pasco JA, Rivadeneira F, Rosengren B, Sornay-Rendu E, Szulc P, Tamaki J, Kanis JA (2016) A Meta-Analysis of Trabecular Bone Score in Fracture Risk Prediction and Its Relationship to FRAX. J Bone Miner Res 31:940–948
167. Hans D (2020) Personal communication. Received by Kanis JA.
168. Kanis JA, Borgstrom F, Compston J, Dreinhofer K, Nolte E, Jonsson L, Lems WF, McCloskey EV, Rizzoli R, Stenmark J (2013) SCOPE: a scorecard for osteoporosis in Europe. Arch Osteoporos 8:144
169. Foundation IO (2010) The Eastern European and central Asian regional audit. Epidemiology, cost and burden of osteoporosis in 2010. Accessed 23rd Sept 2012 www.iofbonehealth.org
170. International Osteoporosis Foundation (2010) Osteoporosis in the European Union in 2008. Ten years of progress and ongoing challenges, International Osteoporosis Foundation (IOF) Accessed 2020-07-24 www.iofbonehealth.org
171. Kanis JA, Johnell O (2005) Requirements for DXA for the management of osteoporosis in Europe. Osteoporos Int 16:229–238

### 3c—Access to DXA

#### Domain

Service provision—Scorecard element

#### Background and aims

The assessment of osteoporosis does not solely depend on the availability of bone mineral density measurements at the lumbar spine and hip with dual-energy X-ray absorptiometry (DXA) (see *Chapter 3b*). Access also depends upon the efficiency with which the technology is used, the ease of patient access (e.g. travelling time), regulatory constraints and barriers to reimbursement.

The aim of this background element was to compare the access to DXA in the EU27+2.

#### Methods

Data were acquired by means of an IOF questionnaire sent to the members of the IOF Committee of National Societies in January 2020. Respondents were asked to update previous estimates for the patient cost, waiting time and reimbursement for DXA. Where an interval was given regarding estimated waiting time or cost, the upper limit was used for the comparison. Respondents were specifically invited to comment on whether the reimbursement policy (or lack of reimbursement) provided barriers to the physician’s assessment of patients.

#### Results

**Fig. 29:**
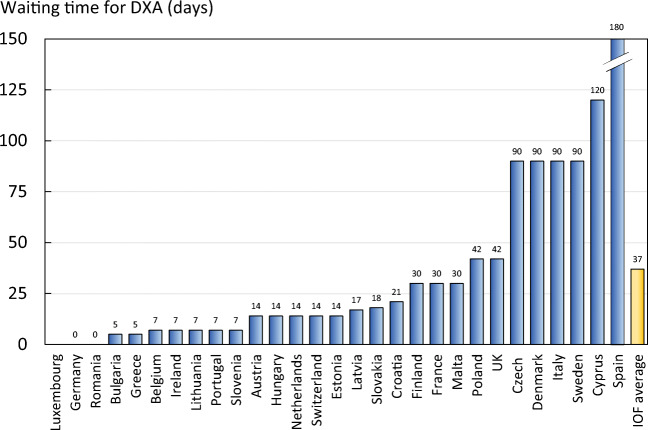
Reported average waiting time for a DXA assessment by country [IOF audit]

The average waiting time for DXA ranged from 0 (Germany and Romania) to 180 days (Spain) (Fig. 29 and Table 32). Ireland and Malta had managed to cut their waiting times significantly since the last IOF audit (Fig. 29) There was no clear relation between waiting times and the availability of DXA (see *Chapter 3b*). For example, the average waiting time in Italy was reported to be 90 days, though the number of DXA machines is high, with 25 machines/million of the general population. This disparity arises because many DXA units are sited in research centres or the private sector and are unavailable to the majority of the population. Conversely, there is only a 5-day waiting time in Bulgaria where the provision of DXA is low (4 DXA units/million). The latter observation reflects the fact that the few machines available are only used to service specialised departments and that BMD assessments are unavailable to the vast majority of the population at risk. A disparity between the availability of equipment and waiting time identifies a heterogeneity in the use of BMD to assess osteoporosis. A further consideration is the uneven geographical location of equipment, which is known to have been problematic in Italy, Spain and the UK.

**Table 32:** Cost and reimbursement of DXA IOF audit, [172]

Country	Waiting time 2019 (d)	Waiting time 2012(d)	Cost (€)	Reimbursement	Barriers to clinical practice
Austria	14	14	50	Yes (conditional)	Reimbursement depending on public/private delivery
Belgium	7	14	93	Yes (conditional)	Reimbursement depending on condition
Bulgaria	5	0	50	No	
Croatia	21		25	Yes	
Cyprus	120	20	70	Yes (conditional)	Reimbursement depending on patient income
Czech Republic	30	40	30	Yes (conditional)	Reimbursement depending on condition
Denmark	90	30	100	Yes	
Estonia	14	14	25	Yes	
Finland	30	1	200	Yes	
France	30	14	40	Yes (conditional)	Reimbursement depending on condition
Germany	0	0	45	Yes	
Greece	5	11	55	Yes (conditional)	Reimbursement depending on condition
Hungary	14	15	20	Yes	
Italy	90	140	90	Yes (conditional)	Reimbursement depending on condition
Ireland	7	83	120	Yes (conditional)	Reimbursement depending on listing with caregiver
Latvia	17	10	3	Yes	
Lithuania	7	6	30	Yes (conditional)	Reimbursement depending on condition
Luxembourg		30			
Malta	30	105	0	Yes	Full reimbursement in public practice
Netherlands	14	14	100	Yes	
Poland	42	1	22	Yes (conditional)	
Portugal	7	8	35	Yes	
Romania	0	7		Yes	
Spain	180	18	90	Yes	
Slovakia	18	11	32	Yes	
Slovenia	7	105	50	Yes (conditional)	
Sweden	90	60	85	Yes	
Switzerland	14		70	Yes (conditional)	
UK	42	11	45	Yes	

Reimbursement for DXA scans varied between member states both in terms of the criteria required and level of reimbursement awarded, and a majority of countries provided full reimbursement (Table 32). In others, reimbursement or partial reimbursement was limited and usually dependent on physician referral for approved indications. In Cyprus, reimbursement was dependent on patient income. Other examples of restricted access included reimbursement only for limited indications (France, Italy, Lithuania, Poland), and only for secondary osteoporosis (Slovenia). The cost of DXA also varied widely (Table 32) and bore little relation to the wealth of the nation or to the availability of DXA machines.

A few countries had made significant cuts in waiting time compared to last audit (Ireland and Malta) whilst some reported higher waiting times in 2019 compared to 2012 (e.g. Czech Republic, Denmark, Cyprus, Spain).

#### Score criteria

The highest score was allocated for unconditional reimbursement. In those countries with restricted reimbursement, countries were identified where reimbursement interfered with what patients could accept or physicians would wish to recommend to patients. Categories are shown in Table 33.

**Table 33:**
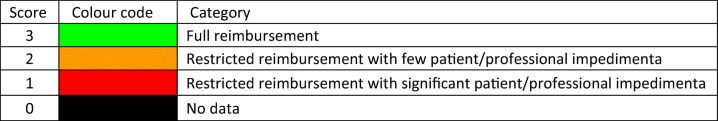
Criteria for allocating scores

#### Score allocation

**Fig. 30:**
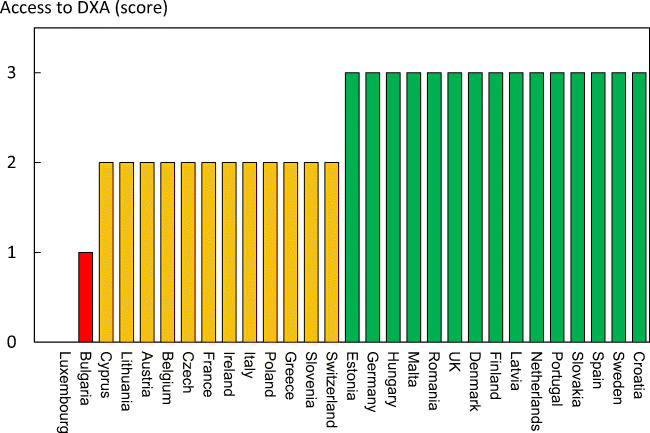
Categorisation of access to DXA by score amongst the member states [IOF audit]

Countries, ranked and categorised by score, are shown in Fig. 30.

#### Comment

There is still a remarkable disparity between the availability of equipment and waiting time, which reflects a heterogeneity in the use of BMD to assess osteoporosis; this disparity was observed previously in the last IOF audit [172] and does not seem to have converged.

#### Reference

172. Kanis JA, Borgstrom F, Compston J, Dreinhofer K, Nolte E, Jonsson L, Lems WF, McCloskey EV, Rizzoli R, Stenmark J (2013) SCOPE: a scorecard for osteoporosis in Europe. Arch Osteoporos 8:144

### 3d—Access to risk assessment algorithms

#### Domain

Service provision—scorecard element

#### Background and aims

The effective targeting of treatment to those at highest risk of fracture requires an assessment of fracture risk. Historically, the targeting of treatment became feasible with the advent of bone mineral density measurements. The causation of fragility fractures is, however, heterogeneous and many additional factors have been identified that contribute to fracture risk. In turn, this has led to the development of risk algorithms that can enhance the assessment of fracture risk to better target interventions, particularly in primary care.

There are several assessment tools available in Europe [173–177]. The most widely used is FRAX®. FRAX is a computer-based algorithm (http://www.shef.ac.uk/FRAX) that calculates the 10-year probability of hip fracture and the 10-year probability of a major fracture (including hip, clinical spine fracture, humerus or wrist fracture). Fracture risk is calculated from age, body mass index and well validated dichotomized risk factors. Femoral neck bone mineral density (BMD) can be optionally input to enhance fracture risk prediction. Fracture probability differs markedly in different regions of the world so that FRAX is calibrated to those countries where the epidemiology of fracture and death is known (currently 65 countries [178]). In addition to the web site, FRAX has been incorporated into the software of densitometers and is available as a smartphone application.

The aim of this scorecard element was to document the availability of country-specific risk assessment models (their uptake is considered separately in *Chapter 4b*). The score was based on the availability of risk assessment models and specific guidance for their use.

#### Methods

The availability of country-specific FRAX models was provided by the FRAX web site. The availability of other risk engines was determined from an IOF questionnaire to the members of the IOF Committee of National Societies undertaken in January 2020, together with a review of country-specific assessment guidelines. The metrics sought were the availability of country-specific risk models and whether national guidance was provided on how results from these assessments should be used in clinical practice.

#### Results

Country-specific fracture risk assessment models are available in 24 of the included countries (Table 34). In Germany, probability-based fracture risk assessment comprises a component of national guidelines but is not FRAX- based [175]. Alternative assessment algorithms are also used in Austria [175] and the Netherlands [173]. In the UK both FRAX and QFracture have been approved [179]. No models are available for Cyprus, Latvia, Luxembourg and Slovenia due to the lack of appropriate epidemiology of fracture on which these could be based. At the time of writing, a FRAX model is developed for Bulgaria but not yet launched. In the countries where a model is available, the majority (21/24) provide guidance on its application to clinical practice. European guidance that can be applied to member states has been published for postmenopausal and glucocorticoid-induced osteoporosis [180, 181].

**Table 34:** Provision of risk assessment models in the member states and guidance on their application to clinical practice

Country	FRAX model available	Other models	Guidance ^b^
Austria	✓	DVO [175]	✓
Belgium	✓		✓
Bulgaria	- ^a^		
Croatia	✓		
Cyprus			
Czech Republic	✓		
Denmark	✓		✓
Estonia	✓		
Finland	✓		✓
France	✓		✓
Germany	✓	DVO [175]	✓
Greece	✓		✓
Hungary	✓		✓
Italy	✓	DeFRA [182]	✓
Ireland	✓		✓
Latvia			
Lithuania	✓		✓
Luxembourg			
Malta	✓		
Netherlands	✓	CBO [173]	✓
Poland	✓		✓
Portugal	✓		✓
Romania	✓		✓
Spain	✓		✓
Slovakia	✓		✓
Slovenia			✓
Sweden	✓		✓
Switzerland	✓	TOP [183]	✓
UK	✓	QFracture [174]	✓

^a^Available 2020

^b^Society, government or academic papers. Sources [184, 185]

#### Score criteria

Criteria for score allocation is presented in Table 35.

**Table 35:**
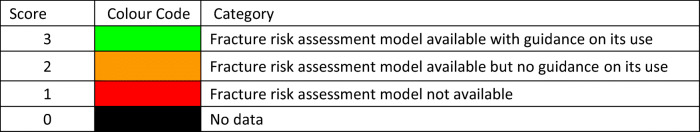
Criteria for allocating scores

#### Score allocation

**Fig. 31:**
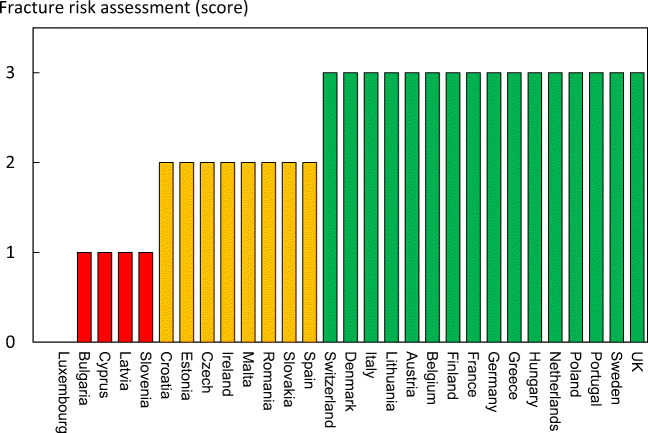
The score assigned to each country on the basis of its provision of fracture risk assessment algorithms

The score was based on availability of country-specific risk models and whether national guidance was provided on how results from these assessments should be used in clinical practice (as given in Table 35). The score assigned to each country is shown in Fig. 31.

#### Comment

Risk assessment models for fractures based on FRAX were available in 24 out of 29 countries. In some countries (Austria, Germany, Netherlands and UK), other models are also available. However, guidance on the use of risk assessment within national guidelines was available in only 15 of the member states. This lack of guidance compared to availability was also observed at the time of the last IOF audit in 2012.

#### References

173. Dutch Institute for Healthcare Improvement (CBO) (2011) Richtlijn Osteoporose en fractuurpreventie, derde herziening. Germany. Accessed April 2020 https://osteoporosevereniging.nl/behandeling/behandelrichtlijn/
174. Hippisley-Cox J, Coupland C (2012) Derivation and validation of updated QFracture algorithm to predict risk of osteoporotic fracture in primary care in the United Kingdom: prospective open cohort study. Bmj 344:e3427
175. Pfeilschifter J, Kurth AA (2011) DVO Guideline 2009 for Prevention, Diagnosis and Therapy of Osteoporosis in Adults Full-Text Version. Osteologie/Osteology 20:55
176. Kanis JA, Johnell O, Oden A, Johansson H, McCloskey E (2008) FRAX and the assessment of fracture probability in men and women from the UK. Osteoporos Int 19:385–397
177. Kanis JA, On behalf of the World Health Organization Scientific Group (2008) Assessment of Osteoporosis at the Primary Health Care Level. WHO Scientific Group technical report. University of Sheffield, UK. Accessed April 2020 http://www.shef.ac.uk/FRAX/pdfs/WHO_Technical_Report.pdf
178. Lekamwasam S (2019) The diversity of Fracture Risk Assessment Tool (FRAX)-based intervention thresholds in Asia. Osteoporos Sarcopenia 5:104–108
179. National Institute for Health and Care Excellence: Clinical Guidelines (2017) Osteoporosis: assessing the risk of fragility fracture. National Institute for Health and Care Excellence (UK). Copyright © NICE 2019., London
180. Kanis JA, McCloskey EV, Johansson H, Cooper C, Rizzoli R, Reginster JYO, , behalf of the Scientific Advisory Board of the European Society for Clinical and Economic Aspects of Osteoporosis (ESCEO) and the Committees of Scientific Advisors and National Societies of the International Osteoporosis Foundation (IOF) (2013) European guidance for the diagnosis and management of osteoporosis in postmenopausal women. Osteoporos Int 24:23-57
181. Lekamwasam SAJ, Agnusdei D, Bilezikian J, Boonen S, Borgström F, Cooper C, Diez Perez A, Eastell R, Hofbauer LC, Kanis JA, Langdahl BL, Lesnyak O, Lorenc R, McCloskey E, Messina OD, Napoli N, Obermayer-Pietsch B, Ralston SH, Sambrook PN, Silverman S, Sosa M, Stepan J, Suppan G, Wahl DA, Compston JE (2012) A framework for the development of guidelines for the management of glucocorticoid-induced osteoporosis. Osteoporosis International 23:2257–2276
182. Nuti R, Brandi ML, Checchia G, Di Munno O, Dominguez L, Falaschi P, Fiore CE, Iolascon G, Maggi S, Michieli R, Migliaccio S, Minisola S, Rossini M, Sessa G, Tarantino U, Toselli A, Isaia GC (2019) Guidelines for the management of osteoporosis and fragility fractures. Intern Emerg Med 14:85–102
183. Osteoporose Plattform SGR (2019) Tool Osteoporose-Plattform (TOP). Accessed April 2020 http://www.osteorheuma.ch/top/
184. Kanis JA, Harvey NC, Cooper C, Johansson H, Oden A, McCloskey EV (2016) A systematic review of intervention thresholds based on FRAX : A report prepared for the National Osteoporosis Guideline Group and the International Osteoporosis Foundation. Arch Osteoporos 11:25
185. International Osteoporosis Foundation (2020) IOF Main Page. IOF. Accessed 2020-07-24 https://www.iofbonehealth.org/

### 3e—Quality of guidelines for assessment and treatment

#### Domain

Service provision—scorecard element

#### Background and aims

The aim of guidelines is to provide an information platform for the assessment and treatment of osteoporosis so that appropriate treatment is directed to individuals at high risk of fracture. Their scope most commonly includes postmenopausal osteoporosis, glucocorticoid-induced osteoporosis and osteoporosis in men. Less commonly, guidelines are available for the assessment of fall risk and its treatment. Ideally, guidelines should be based on systematic literature reviews and any recommendations supported by an adequate level of evidence.

The aim of this scorecard element was to determine the scope and quality of guidelines available in the member states.

#### Methods

Data were acquired by an IOF questionnaire to the members of the IOF Committee of National Societies undertaken in January 2020. Respondents were asked whether national guidelines were available for the assessment and/or treatment of osteoporosis. Responses were used to update earlier audits of the IOF [186–188]. Where guidelines were available, additional information was requested on their scope and quality.

Additional information regarding scope of guideline included whether it relates to postmenopausal women (PMW), men or glucocorticoid-induced osteoporosis (GIOP). Information on quality of guideline included grading according to the criteria of the Appraisal of Guidelines for Research & Evaluation (AGREE) consortium [189] under seven general domains^1^.

A positive response in each AGREE category contributed a point to the score (for a maximum increase of 7 points). Up to 3 additional points were given for the scope of the guideline (PMW, men, GIOP) to give a maximum total score of 10. Where more than one guideline was available, a composite mean was used.

#### Results

Guidelines for the management of osteoporosis were available in the majority of member states (unavailable in Cyprus and Malta). All of the remaining countries had guidelines available for postmenopausal women (Table 36). 25 of 27 countries had guidelines specifically for osteoporosis in men and 23 had guidelines for secondary osteoporosis including glucocorticoid-induced osteoporosis. These numbers are significantly higher than the last IOF audit in 2012. It is however important to note that the availability of guidelines does not necessarily improve disease management, and in some countries (e.g. Croatia, Portugal, Slovenia and UK) multiple guidelines were available from different sources which likely confuses rather than clarifies clinical practice.

**Table 36:** Availability and scope of guidelines for the assessment and treatment of osteoporosis amongst the member states [IOF audit]

Country	Developed or updated (year)	Scope^a^	AGREE criteria	Score
Austria	2010-2017	PMW, men, GIOP	7	10
Belgium	2020	PMW, men, GIOP	4	7
Bulgaria	2019	PMW, men, GIOP	5	8
Croatia	2013	PMW, men, GIOP	3	6
Cyprus			0	0
Czech Republic	2015-2018	PMW, men, GIOP	3	6
Denmark	2019	PMW, men, GIOP	2	5
Estonia	2007	PMW, men	4	6
Finland^b^	2013	PMW, men, GIOP	6	9
France	2018-2020	PMW, men, GIOP	6	9
Germany	2017	PMW, men, GIOP	7	10
Greece	2019	PMW, men	6	8
Hungary	2010	PMW, men, GIOP	7	10
Italy	2016	PMW, men, GIOP	7	10
Ireland	2011	PMW, men, GIOP	2	5
Latvia	2012	PMW, men, GIOP	7	10
Lithuania	2011	PMW	6	7
Luxembourg^c^	2010	PMW	0	0
Malta			0	0
Netherlands	2012	PMW, men, GIOP	7	10
Poland	2018	PMW, men, GIOP	5	8
Portugal	2018	PMW, men, GIOP	7	10
Romania	2010	PMW, men, GIOP	6	9
Spain	2015	PMW, men, GIOP	6	9
Slovakia^b^	2010	PMW, men, GIOP	7	10
Slovenia	2018	PMW, men, GIOP	3	6
Sweden	2020	PMW, men, GIOP	6	9
Switzerland	2019	PMW, men, GIOP	4	7
UK	2019 (Scotland)	PMW, men, GIOP	7	10

^a^PMW stands for postmenopausal women, GIOP stands for glucocorticoid-induced osteoporosis

^b^Information on year for last update of guidelines based on previous IOF audit [188]

^c^Based on previous IOF audit [188]

#### Score criteria

Scores for each country were categorised as shown in Table 37.

**Table 37:**
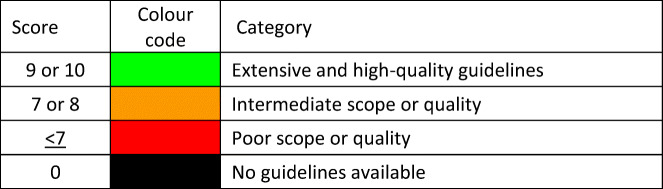
Criteria for allocating scores

#### Score allocation

**Fig. 32:**
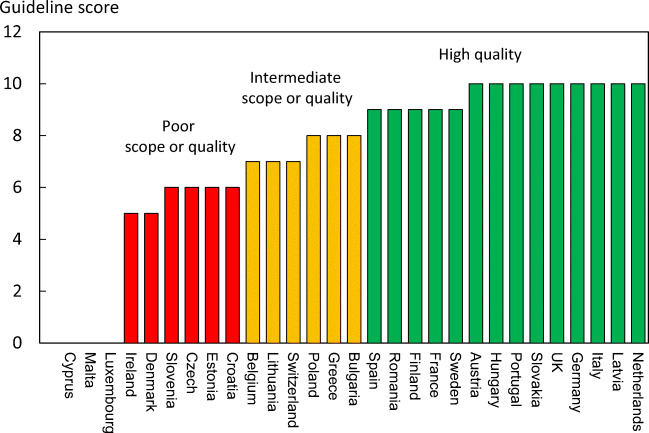
Score allocation based on the scope and quality of guidelines available for the assessment and treatment of osteoporosis. For the UK, the score for guidance provided by NICE is 8 and that provided by the National Osteoporosis Guidelines Group has a score of 10 [IOF audit]

The score allocation and grade for each country is shown in Fig. 32.

Half of the member states (13/26) reported having high-quality guidelines. Three member states did not report (Cyprus, Malta, Luxembourg). Compared to last audit [188], the proportion of countries reporting high-quality guidelines was higher in 2020 than 2012 whilst the proportion of reporting countries is the same.

#### Comment

There was an extensive variation in the contents and quality of national guidelines according to the AGREE criteria. It should be noted that a high score reflects high quality of the process, but not necessarily quality of the content. Several countries improved their score compared to last audit (2012), e.g. France, Poland and Spain.

#### References

186. International Osteoporosis Foundation (2010) Osteoporosis in the European Union in 2008. Ten years of progress and ongoing challenges. International Osteoporosis Foundation (IOF). Accessed 2020-07-24 www.iofbonehealth.org
187. International Osteoporosis Foundation (IOF), on behalf of the Scientific Advisory Board of the European Society for Clinical and Economic Aspects of Osteoporosis (ESCEO) and the Committees of Scientific Advisors and National Societies of the (2010) The Eastern European & Central Asian Regional Audit Epidemiology, costs & burden of osteoporosis in 2010. IOF. Accessed 2020-07-24 https://www.iofbonehealth.org/sites/default/files/PDFs/Audit%20Eastern%20Europe_Central%20Asia/Eastern_European_Central_Asian_Audit_2010.pdf
188. Kanis JA, Borgstrom F, Compston J, Dreinhofer K, Nolte E, Jonsson L, Lems WF, McCloskey EV, Rizzoli R, Stenmark J (2013) SCOPE: a scorecard for osteoporosis in Europe. Arch Osteoporos 8:144
189. The AGREE Next Steps Consortium (2009) Appraisal of Guidelines for Research and Evaluation II. Accessed 2020-07-24 https://www.agreetrust.org/wp-content/uploads/2013/10/AGREE-II-Users-Manual-and-23-item-Instrument_2009_UPDATE_2013.pdf

### 3f—Guideline criteria for assessment and treatment

#### Domain

Service provision—supplementary information

#### Background and aims

The aim of this section was to summarise the differences in the application of guidelines to clinical practice and, in particular, to identify where guideline recommendations conflicted with reimbursement policy.

#### Methods

Data were acquired from a structured IOF questionnaire administered to the members of the IOF Committee of National Societies undertaken in January 2020. Information requested included whether guidelines addressed population-based screening, the tools used for assessment, and the tools to decide eligibility for treatment. An enquiry was also made whether risk assessment or treatment recommendations were compatible with reimbursement policy.

#### Results

Guidelines were generally less than 10 years old, because of updating, with one exception (Estonia, 2002). Guidelines were not available in Cyprus or Malta, although Cyprus to some degree follows the Greek national guidelines. Population screening was addressed in guidelines from 14 of 24 countries. Although reviewed, population-based screening was not recommended. However, several countries reported indications for BMD screening dependent on certain risk factors such as age (e.g. Greece) and prior fracture (e.g. France). In Portugal, all individuals above 50 years were reported to be recommended to have a FRAX evaluation [190].

Guidelines in more than 80% of the cases covered assessment of fracture risk. The most common factors considered in fracture risk assessment were bone mineral density (21 countries), age (20 countries) and prior fracture (20 countries). The use of fracture risk assessment algorithms was less consistent and noted in 17 countries. FRAX was the most widely used instrument, though in Germany the DVO tool was recommended [191]. DVO is also used in Austria. In the UK, both FRAX and QFracture have been approved [192]. Guidelines in all countries covered eligibility for treatment with a general commonality of approach. Eligibility for treatment depended on, e.g. prior fracture (all countries), bone mineral density (all countries) and glucocorticoid-induced osteoporosis (except Germany and Greece). Risk assessment tools provided criteria for intervention consistent with reimbursement in 16 of 27 countries.

Several countries reported incompatibilities between recommendations for risk assessment or treatment with reimbursement policy (Table 38). For example, bone densitometry is not reimbursed (Bulgaria), or FRAX is not included in the reimbursement policy (Czech Republic, Switzerland). One problem, inconsistently related to reimbursement, was that multiple sets of guidelines are available, potentially giving conflicting recommendations (Croatia, Poland, Sweden, UK). Another barrier to treatment is that reimbursement may only be granted where the prescription was issued by a specialist (see *Chapter 3a*).

**Table 38:** Scope of guidelines for patient assessment, treatment and consistency with reimbursement policy [IOF audit]

Country	Developed or updated (year)	Assessment	Compatible/consistent	Treatment	Compatible/consistent
Austria	2010	Yes	Yes	Yes	Yes
Belgium	2020	Yes	No	Yes	No
Bulgaria	2019	Yes	No	Yes	Yes
Croatia	2013	No	Yes	Yes	Yes
Cyprus	na	-	-	-	-
Czech Republic	2015-2018	Yes	No	Yes	Yes
Denmark	2019	No	Yes	Yes	Yes
Estonia	2007		Yes	Yes	Yes
Finland	2013	Yes	Yes/No	Yes	Yes
France	2018	Yes	Yes	Yes	Yes
Germany	2017	Yes	Yes	Yes	Yes
Greece	2019	Yes	Yes	Yes	Yes
Hungary	2010	Yes	Yes	Yes	Yes
Italy	2016	Yes	Yes	Yes	Yes
Ireland	2011	Yes	Yes	Yes	Yes
Latvia	2012	Yes	Yes	Yes	No
Lithuania	2011	Yes	Yes	Yes	Yes
Luxembourg	2010	nr	nr	nr	nr
Malta	na	-	-	-	-
Netherlands	2012	No	Yes	Yes	Yes
Poland	2018	Yes			
Portugal	2018	Yes	Yes	Yes	Yes
Romania	2010				
Spain	2015	No	Yes	Yes	Yes
Slovakia	2010	Yes	Yes	Yes	Yes
Slovenia	2018		Yes	Yes	Yes
Sweden	2020	Yes	Yes	Yes	
Switzerland	2019	Yes	Yes	Yes	Yes
UK	2019 (Scotland)	Yes	Yes	Yes	Yes

#### Score criteria and score allocation

Supplementary information, no score criteria and allocation

#### Comment

Risk assessment and treatment recommendations were compatible with reimbursement policy in a majority of the member states. This is a slightly higher proportion (59%) than at the last IOF audit performed in 2012 (52%) [193]. However, one third of the member states reported inconsistency or non-compatibility between guidelines, and reimbursement policy.

#### References

190. Rodrigues AM, Canhão H, Marques A, Ambrósio C, Borges J, Coelho P, Costa L, Fernandes S, Gonçalves I, Gonçalves M, Guerra M, Marques ML, Pimenta S, Pinto P, Sequeira G, Simões E, Teixeira L, Vaz C, Vieira-Sousa E, Vieira R, Alvarenga F, Araújo F, Barcelos A, Barcelos F, Barros R, Bernardes M, Canas da Silva J, Cordeiro A, Costa M, Cunha-Miranda L, Cruz M, Duarte AC, Duarte C, Faustino A, Figueiredo G, Fonseca JE, Furtado C, Gomes J, Lopes C, Mourão AF, Oliveira M, Pimentel-Santos FM, Ribeiro A, Sampaio da Nóvoa T, Santiago M, Silva C, Silva-Dinis A, Sousa S, Tavares-Costa J, Terroso G, Vilar A, Branco JC, Tavares V, Romeu JC, da Silva J (2018) Portuguese recommendations for the prevention, diagnosis and management of primary osteoporosis - 2018 update. Acta Reumatol Port 43:10–31
191. Pfeilschifter J, Kurth AA (2011) DVO Guideline 2009 for Prevention, Diagnosis and Therapy of Osteoporosis in Adults Full-Text Version. Osteologie/Osteology 20:55
192. National Institute for Health and Care Excellence: Clinical Guidelines (2017) Osteoporosis: assessing the risk of fragility fracture. National Institute for Health and Care Excellence (UK). Copyright © NICE 2019., London
193. Kanis JA, Borgstrom F, Compston J, Dreinhofer K, Nolte E, Jonsson L, Lems WF, McCloskey EV, Rizzoli R, Stenmark J (2013) SCOPE: a scorecard for osteoporosis in Europe. Arch Osteoporos 8:144

### 3g – Fracture liaison services

#### Domain

Service provision—scorecard element

#### Background and aims

Fracture liaison services (FLS), also known as osteoporosis coordinator programmes and care manager programmes, provide a system for the routine assessment and management of postmenopausal women and older men who have sustained a low trauma fracture [194, 195]. Assessment includes DXA measurements, fall risk evaluation, and assessment of underlying secondary causes of osteoporosis. Although the importance of an incident fracture as a risk factor for further fracture is well recognised, the majority of patients presenting with a low trauma fracture do not receive appropriate assessment and treatment in the setting of standard hospital care. FLS address this need through a systematic approach to identifying such individuals and assessing their risk of further fractures and the need for treatment. Most FLS are based in secondary care although models in primary care have also been described. The clinical and cost- effectiveness of FLS has been demonstrated in several centres [196, 197].

The aim of this scorecard element was to document the proportion of hospitals that have a fracture liaison service in the countries surveyed.

#### Methods

Information was acquired from a structured IOF questionnaire administered to the members of the IOF Committee of National Societies undertaken in January 2020. Correspondents were asked to estimate the proportion of hospitals in each country that have a scheme in place that refers fracture patients over 50 years old to a fracture liaison service. Scoring was based on the distribution of the estimates.

#### Results

No fracture liaison services were reported from Bulgaria, Croatia, Cyprus, Estonia, Latvia, Lithuania, Romania and Slovenia. The presence of FLS was acknowledged in the remaining member states, but for approximately half of these countries, the proportion of hospitals that have a scheme in place was less than 10%. Finland, Ireland and Sweden reported rates above 25% whilst Malta, Netherlands and UK reported that more than 50% of hospitals have a procedure in place for referral of fracture patients to a fracture liaison service. As thresholds for scoring have changed since last audit, scores from the previous audit were not considered.

#### Score criteria

The proportion of hospitals in each member state that had fracture liaison services (FLS) in place were categorised as shown in Table 39.

**Table 39:**
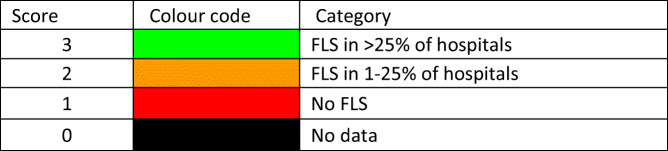
Criteria for allocating scores

#### Score allocation

**Fig. 33:**
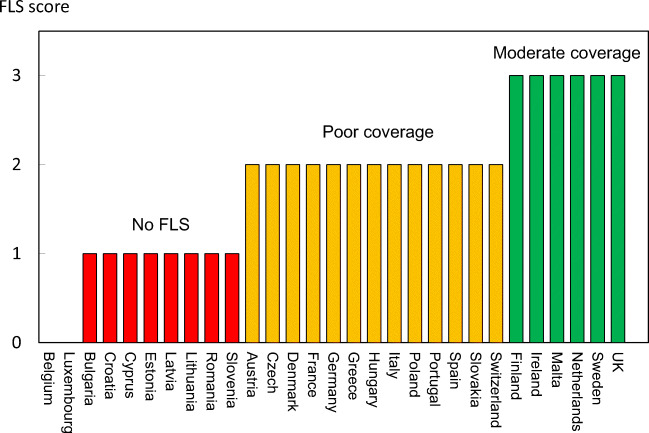
Scores allocated by country on the availability of fracture liaison services in hospitals by country [IOF audit]

Figure 33 shows the scores allocated by country.

#### Comment

The information provided on FLS needs to be interpreted cautiously. It provides a perception of how many hospitals of a country has a fracture nurse working in a fracture liaison service, but it is an expert opinion and not based on numerical evidence. Moreover, no account was taken of FLS in primary care. In addition, no information was available on the performance of the FLS. A general improvement of FLS rates is observed since the last IOF audit was performed (2012), with significantly higher rates reported for several member states. It is however notable that a colour code of green should not be interpreted as an endorsement since provision should, in the view of the committee, be expected in the majority of hospitals or care centres.

One effort to encourage cooperation between FLS providers is Capture the Fracture® (CtF), a global initiative of IOF to ‘facilitate the implementation of coordinated, multidisciplinary models of care for secondary fracture prevention’ [198, 199]. CtF has created a set of internationally endorsed standards and guides for best practice and has assembled the largest network of individual FLS providers in the world. CtF provides resources, tools and educational programmes to bridge the gap between FLS providers and helps in the creation of new FLS. This growing network of FLS providers is mapped on their website (https://www.capturethefracture.org/map-of-best-practice).

#### References

194. Mitchell PJ (2011) Fracture Liaison Services: the UK experience. Osteoporos Int 22(Suppl 3):487–494
195. Nakayama A, Major G, Holliday E, Attia J, Bogduk N (2016) Evidence of effectiveness of a fracture liaison service to reduce the re-fracture rate. Osteoporos Int 27:873–879
196. Axelsson KF, Johansson H, Lundh D, Möller M, Lorentzon M (2020) Association Between Recurrent Fracture Risk and Implementation of Fracture Liaison Services in Four Swedish Hospitals: A Cohort Study. J Bone Miner Res 35:1216–1223
197. Wu CH, Tu ST, Chang YF, Chan DC, Chien JT, Lin CH, Singh S, Dasari M, Chen JF, Tsai KS (2018) Fracture liaison services improve outcomes of patients with osteoporosis-related fractures: A systematic literature review and meta-analysis. Bone 111:92–100
198. International Osteoporosis Foundation (2012) Capture the Fracture Report:2012
199. Javaid MK, Kyer C, Mitchell PJ, Chana J, Moss C, Edwards MH, McLellan AR, Stenmark J, Pierroz DD, Schneider MC, Kanis JA, Akesson K, Cooper C (2015) Effective secondary fracture prevention: implementation of a global benchmarking of clinical quality using the IOF Capture the Fracture® Best Practice Framework tool. Osteoporos Int 26:2573–2578

### 3h—Use of quality indicators

#### Domain

Service provision—scorecard element

#### Background and aims

The use of indicators to systematically measure the quality of care provided to people with osteoporosis or associated fractures has expanded as a discipline within the past decade, as shown for use of healthcare quality indicators more broadly [200, 201]. In the UK, the Department of Health Best Practice Tariff for hip fracture care has used financial incentives since April 2010 to drive adherence with the six core benchmarks, which include an assessment of bone health and risk of falling. In the two years following introduction of the tariff, the proportion of patients with fragility hip fracture for whom all six standards were met rose from 24 to 55% [202]. Patient-level key performance indicators have been set by the International Osteoporosis Foundation to measure the effectiveness of fracture liaison services and guide quality improvement [203].

The aim of this scorecard element was to document the systematic approaches to enhance the quality of osteoporosis care or secondary prevention of fragility fractures amongst the member states.

#### Methods

Data were acquired via an IOF questionnaire to the members of the IOF Committee of National Societies, undertaken in January 2020. Respondents were asked whether national systems were in place that systematically collect data on the quality of care provided to people with osteoporosis or the secondary prevention of fragility fractures. Further questions covered whether the systems use measures (quality indicators or standards) that are documented on a regular basis and, if so, with what frequency.

#### Results

Ten out of 29 member states have systems that include quality measures plus a regular audit for national healthcare agencies (Denmark, Finland, Germany, Italy, Ireland, Netherlands, Slovakia, Slovenia, Sweden and UK) as presented in Table 40.

**Table 40:** National systems that provide quality indicators in the context of osteoporosis and fractures in the member states [IOF audit]

Country	Quality indicator score (2019)	Quality indicator score (2012)	Systems in place	Targets
Austria	1	1	No	
Belgium	1	1	No	
Bulgaria	1	2	No	
Croatia	1	0	No	
Cyprus	1	1	No	
Czech Republic	1	1	No	
Denmark	3	3	Yes	Hip fractures
Estonia	1	1	No	
Finland	3	2	Yes	Hip fractures, other fractures
France	1	1	No	
Germany	3	3	Yes	Hip fractures, osteoporosis
Greece	1	2	No	
Hungary	1	2	No	
Italy	3	1	Yes	Pharmacological treatment
Ireland	3	1	Yes	Hip fractures
Latvia	1	1	No	
Lithuania	1	1	No	
Luxembourg	0	1	-	
Malta	1	1	No	
Netherlands	3	3	Yes	All fractures
Poland	1	1	No	
Portugal	1	1	No	
Romania	1	1	No	
Spain	1	1	No	
Slovakia	3	2	Yes	Osteoporosis, fragility fractures, falls
Slovenia	3	1	Yes	Hip fractures
Sweden	3	2	Yes	Hip fracture, pharmacological treatment
Switzerland	1	0	No	
UK	3	3	Yes	Hip fractures, osteoporosis, fragility fractures

In the UK, osteoporosis/secondary prevention of fragility fractures has been included in the Quality and Outcomes Framework as part of the general practitioner contract since April 2012 [204]. The Quality and Outcomes Framework is a pay for performance scheme for general practice in the UK, which awards ‘achievement points’ for adhering to procedural and treatment guidelines and meeting intermediate outcome targets for over 130 quality indicators. In the UK, there is also a system of clinical audits in place, seeking to improve patient care and outcomes through systematic review of care according to explicit criteria and the implementation of change. These include the National Audit of Falls [205] and Bone Health in Older People [206] and the continuous Falls and Fragility Fracture Audit [207]. In Germany, selected providers and health insurance funds have, in the framework of ‘integrated care contracts’ entered into agreements on coordinated osteoporosis care which may include the documentation of care standards to enable tracking of the quality of care provided [208]. The nature and contents of these contracts vary across regions [209]. There is a systematic and nationwide collection of quality indicators for the inpatient care following hip fracture [210], however a systematic collection of indicators that would permit assessment of care quality of those with osteoporosis and in the secondary prevention of fragility fractures is not in place. Germany, Ireland, Sweden and UK also reported having systems on regional level for reporting/monitoring of quality measures.

#### Score criteria

The score was based on the presence of systems and their use as quality indicators as given in Table 41.

**Table 41:**
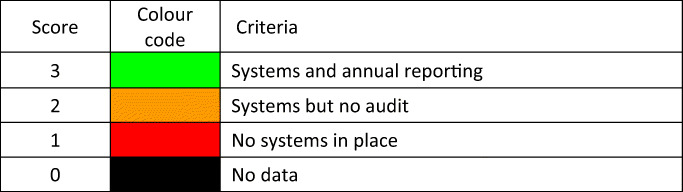
Criteria for allocating scores

#### Score allocation

**Fig. 34:**
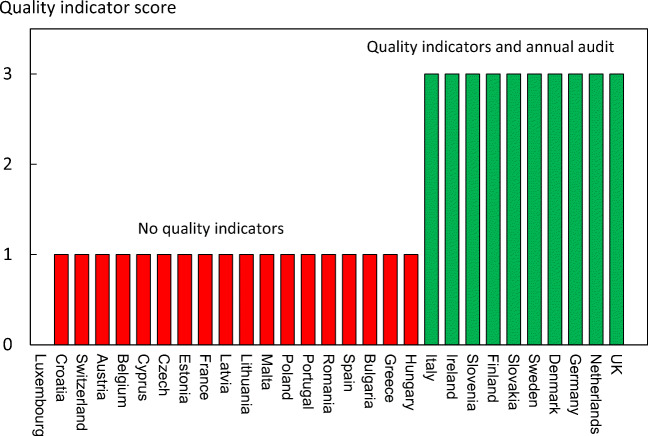
Score allocation for quality indicators by country [IOF audit]

Score allocation for quality indicators by country are shown in Fig. 34.

#### Comment

There are relatively few studies that have tested quality improvement strategies in osteoporosis and most (>80%) are from North America. A recent comprehensive systematic review identified fracture liaison services and patient self-scheduling of DXA as quality improvement strategies [211]. Given the relative novelty of using quality indicators for the tracking of quality of care provided to patients with osteoporosis or associated fractures in the European region, it should be recognised that the score is a ‘proxy’ measure. Audited quality measures have been introduced in some countries. In France, it is reported that development of a system for systematic evaluation of osteoporosis management is in progress. Ten member states report that there are annual follow-ups on quality indicators, compared to four at the time of the previous audit (2012). Thus, several countries have progressed towards systematic monitoring of clinical management of the osteoporosis patient.

#### References

200. Allen P, Pilar M, Walsh-Bailey C, Hooley C, Mazzucca S, Lewis CC, Mettert KD, Dorsey CN, Purtle J, Kepper MM, Baumann AA, Brownson RC (2020) Quantitative measures of health policy implementation determinants and outcomes: a systematic review. Implement Sci 15:47
201. Nolte E, Roland M, Damberg CL, Mattke S, Cacace M, Goshev S, Brereton L, Conklin A, Hiatt L, D. Q, L. L (2011) Informing the development of a resource allocation framework in the German healthcare system. Santa Monica CA, USA. https://www.rand.org/pubs/technical_reports/TR946.html
202. The National Hip Fracture Database (2020) Part of the Falls and Fragility Fracture Audit Programme. Royal College of Physicians. Accessed 2020-07-24 https://www.nhfd.co.uk/
203. Javaid MK, Sami A, Lems W, Mitchell P, Thomas T, Singer A, Speerin R, Fujita M, Pierroz DD, Akesson K, Halbout P, Ferrari S, Cooper C (2020) A patient-level key performance indicator set to measure the effectiveness of fracture liaison services and guide quality improvement: a position paper of the IOF Capture the Fracture Working Group, National Osteoporosis Foundation and Fragility Fracture Network. Osteoporos Int 31:1193–1204
204. NHS Employers and British Medical Association (2012) Quality and Outcomes Framework for 2012/13. Guidance for PCOs and practices. UK. http://www.wales.nhs.uk/sites3/Documents/480/20120301%20-%20Guidance%20-%20NHS%20Employers%20-%20Quality%20%26%20Outcomes%20Framework%202012-13.pdf
205. National Falls Prevention Coordination Group (NFPCG) (2017) Falls and fracture consensus statement - Supporting commissioning for prevention. Accessed 2020-10-09 https://www.england.nhs.uk/south/wp-content/uploads/sites/6/2017/03/falls-fracture.pdf
206. Treml J, Husk J, Lowe D, Vasilakis N (2011) Falling standards, broken promises: report of the national audit of falls and bone health. https://www.rcplondon.ac.uk/projects/outputs/falling-standards-broken-promises-report-national-audit-falls-and-bone-health
207. Healthcare Quality Improvement Partnership (HQIP) (2020) Falls and Fragility Fracture Audit (includes the Hip Fracture Database) (FFFAP). Accessed 2020-10-09 https://www.hqip.org.uk/a-z-of-nca/falls-and-fragility-fractures-includes-the-hip-fracture-database/#.X4Akz1wzaUl
208. Geraedts M, Mehl C, Schmitz J, Siegel A, Graf E, Stelzer D, Farin-Glattacker E, Ihle P, Köster I, Dröge P, Günster C, Haas N, Gröne O, Schubert I (2020) Development of an indicator set for the evaluation of the population-based integrated healthcare model 'Gesundes Kinzigtal' (Healthy Kinzigtal). Z Evid Fortbild Qual Gesundhwes 150-152:54–64
209. Grothaus F-J (2009) Entwicklung der integrierten Versorgung in der Bundesrepublik Deutschland 2004 – 2008. Gemeinsame Registrierungsstelle zur Unterstützung der Umsetzung des §140d SGB V. https://docplayer.org/7872789-Entwicklung-der-integrierten-versorgung-in-der-bundesrepublik-deutschland-2004-2008.htm
210. AQUA (2011) Beschreibung der Qualitätsindikatoren für das Erfassungsjahr 2011 - Hüftgelenknahe Femurfraktur. Qualitätsindikatoren 2011. Germany. https://www.sqg.de/downloads/QIDB/2011/AQUA_17n1_Indikatoren_2011.pdf
211. Nayak S, Greenspan SL (2018) How Can We Improve Osteoporosis Care? A Systematic Review and Meta-Analysis of the Efficacy of Quality Improvement Strategies for Osteoporosis. J Bone Miner Res 33:1585–1594

## Chapter 4: Service uptake

### 4a—Uptake of DXA

#### Domain

Service uptake—Background information

#### Background and aims

The assessment of osteoporosis depends in part on the measurement of bone mineral density at the lumbar spine and hip with dual energy X-ray absorptiometry (DXA). The requirement for the technology will depend on the assessment guidelines in each member state and the policy with respect to the use of DXA to diagnose osteoporosis, often for reimbursement purposes, and to monitor treatment. The uptake of this technology depends upon the efficiency with which the technology is used, the ease of patient access (e.g. travelling time), regulatory constraints and barriers to reimbursement.

The aim of this element was to compare the access to DXA measured as a function of the requirements recommended in relevant assessment guidelines.

#### Methods

Ideally, uptake should be measured as the number of scans undertaken in relation to treatment guidelines in each member state. Such data are not available in the EU as a whole. Data were available by age and sex from the National Health Service of Denmark in 2005 and more recently in 2020 (Sundhedsdatastyrelsen) [212].

#### Results

**Fig. 35:**
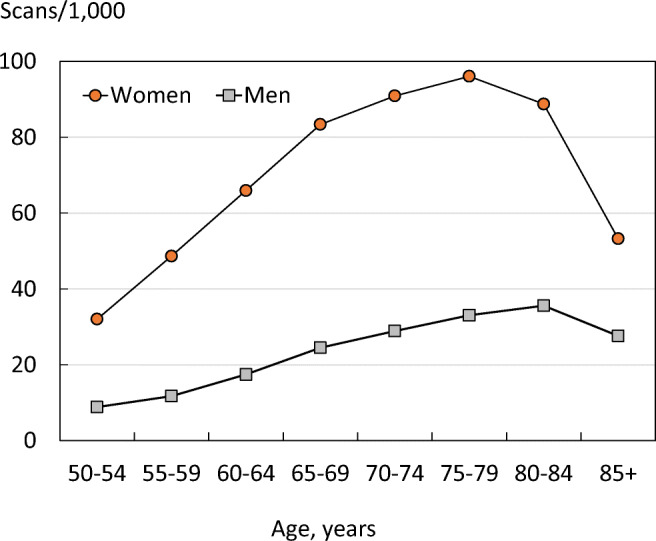
The uptake of BMD testing in men and women by age in Denmark in 2020 [Data kindly provided by Bo Abrahamsen, Department of Clinical Research, University of Southern Denmark and Holbæk Hospital, Denmark]

The Danish National Health Service uptake of BMD testing by age and sex for 2020 is shown in Fig. 35. Although the accuracy of the claims to tests is uncertain and tests in the private sector are not captured, the uptake is low even accounting for these errors. Thus, guidelines based on BMD testing as extant in Denmark [213] indicate that 185 women/1000 of the women aged 50 years or more qualify for BMD testing or to monitor treatment [214, 215] whereas the corresponding figure for Denmark is 67/1000 or 36% of the desired uptake. The use of probability-based guidelines reduces the number of scans needed to 81/1000 women [215] but is still higher than that attained in Denmark. The uptake in men over the age of 50 years is 4 times lower in men.

**Fig. 36:**
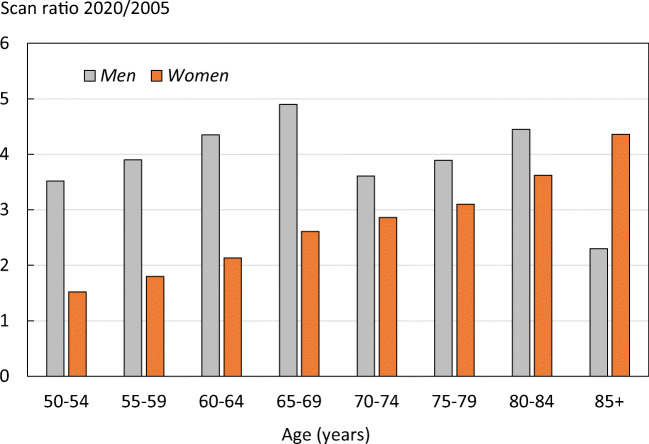
The uptake of BMD testing in women by age in Denmark in 2005 and 2020 [Data kindly provided by Bo Abrahamsen, Department of Clinical Research, University of Southern Denmark and Holbæk Hospital, Denmark]

Nonetheless, there has been a marked increase in the uptake of BMD testing since 2005. In women age 50 years or more, the rate of BMD testing was 28.6/1000 and had increased by more than 2-fold by 2020. In men, the increase was approximately four-fold (Fig. 36).

#### Score allocation

No score allocation

#### Comment

More information is required from all European countries on the utilization of DXA with regard to guidelines on the assessment and monitoring of treatment. The available evidence from Denmark, a country moderately provided with DXA machines, is that service uptake is less than optimal.

#### References

212. Abrahamsen B (2020) Uptake of DXA - Personal communication. Received by Kanis JA. Data from Sundhedsdatastyrelsen in 2020, for the uptake of DXA https://sundhedsdatastyrelsen.dk/da
213. Hitz M, Harsløf T, Ejersted C, Bech-Jensen J-E, Brockstedt H, Vestergaard P, Frost M, Langdahl B (2019) Treatment guidance in postmenopausal osteoporosis in women. Danish Endocrinological Society. http://www.endocrinology.dk/index.php/3-calcium-og-knoglemetaboliske-sygdomme/3-osteoporose
214. Kanis JA, Johnell O (2005) Requirements for DXA for the management of osteoporosis in Europe. Osteoporos Int 16:229–238
215. Johansson H, Kanis JA, Oden A, Compston J, McCloskey E (2012) A comparison of case-finding strategies in the UK for the management of hip fractures. Osteoporos Int 23:907–915

### 4b—Uptake of risk assessment algorithms

#### Domain

Service uptake—scorecard element

#### Background and aims

FRAX® is an algorithm that determines fracture probability in individuals by integrating the weight of important clinical risk factors for fracture and mortality risk, with or without information on BMD (see *Chapter 1 g*). Each tool is country-specific and calibrated to the national epidemiology of fracture. They were developed by the then WHO Collaborating Centre for Metabolic Bone Diseases at Sheffield, UK, and launched in 2008 [216, 217]. The FRAX tools (www.shef.ac.uk/FRAX) compute the 10-year probability of hip fracture or a major osteoporotic fracture. A major osteoporotic fracture is a clinical spine, hip, forearm and humerus fracture. The use of the tool improves risk assessment compared to the use of BMD alone.

FRAX is now a component of many national guidelines for the assessment of osteoporosis (see *Chapter 3f*) and European guidelines for postmenopausal osteoporosis and glucocorticoid-induced osteoporosis [218, 219]. The aim of this element was to determine the use of FRAX in the EU27+2 countries.

#### Methods

Each FRAX model on the web counts the number of calculations performed for that particular country. A problem with these data is that some countries, particularly those without a country-specific FRAX model, may use a surrogate. For example, the UK model was adopted as a surrogate in Poland before the advent of a Polish model and the Greek model is presently used in Cyprus. For this reason, we assessed the number of sessions by the source of the calculation [Google Analytics]. Currently, a session is defined as a group of interactions with the website that any one user can make within a 30-min time frame; it underestimates fracture risk calculations (by approximately 30%) as more than one calculation can occur within the session [220]. FRAX usage was computed as the number of sessions originating from each country and expressed as sessions/million people in the general population over a period of one year (1^st^ January 2019 to the 31st December 2019).

#### Results

The web-based usage of the models is provided in Table 42, which shows considerable heterogeneity in uptake. Slovenia, the UK, Sweden and Greece had the highest use of FRAX. Latvia, Lithuania, Germany and Bulgaria had the lowest. The average uptake for the EU27+2 was 1,555 sessions/million people in the general population. Country-specific models are available in 24 of the EU27+2 (see *Chapter 3d*). FRAX models were not available for Cyprus, Latvia, Luxembourg and Slovenia. A Bulgarian model is available but was not live at the time of data acquisition. There was, however, no clear relationship between the availability of a country-specific model and the use of FRAX.

**Table 42:** Uptake of FRAX in EU27+2 counties expressed as the number of sessions per million of the general population

Country	Sessions/million		Country	Sessions/million	
	2011	2019		2011	2019
Austria	1,534	2,439	Italy	518	414
Belgium	5,003	2,144	Latvia	57.7	218
Bulgaria	112	49	Lithuania	28.5	131
Croatia	-	629	Luxembourg	2,293	507
Cyprus	272	1,058	Malta	16,988	1,541
Czech Rep.	175	344	Netherlands	526	609
Denmark	942	319	Poland	338	513
Estonia	207	916	Portugal	1,039	2,662
Finland	444	4,343	Romania	230	463
France	314	676	Slovakia	372	504
Germany	83.5	93	Slovenia	1,322	41,874
Greece	502	4,566	Spain	1,115	1,527
Hungary	1,205	2,832	Sweden	1,911	5,306
Ireland	1,643	2,623	Switzerland	-	3,702
			UK	2,293	5,443

Comparative data in 2011 were available for 26 countries. In most countries, the number of FRAX sessions had increased. On average, the increase was more than twofold (2.45). Since 2011, 42% (*n* = 11) of the 26 countries had improved in grade (i.e. from low to intermediate use, or intermediate to high use), while 12% (*n* = 3) had decreased in grade. The number of sessions per million increased in 21 countries (81%). The greatest increases in uptake of FRAX usage (both in absolute terms and by percentage change) were noted in Finland and Greece. The UK and Sweden also had high absolute increases in the number of sessions /million people in the general population.

#### Score criteria

FRAX sessions/million people in the general population for one year was approximately categorised into tertiles and provided in Table 43.

**Table 43:**
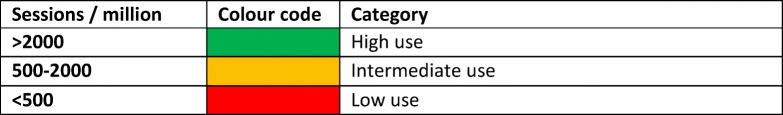
Criteria for allocating scores

#### Score allocation

**Fig. 37:**
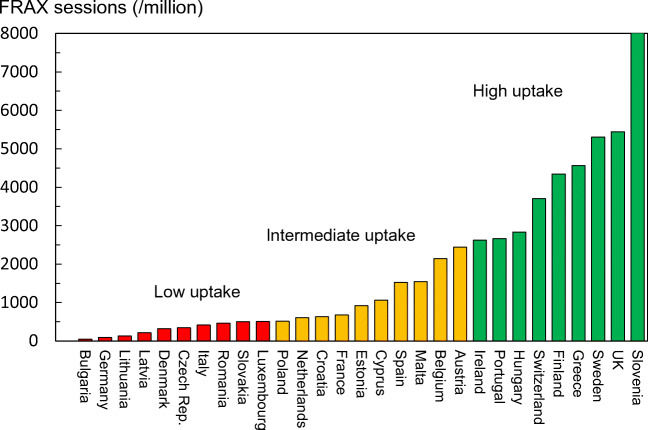
The uptake of fracture risk assessment tools as judged by the use of FRAX from each EU27+2 country by score category

Countries, ranked and categorised by score, are shown in Fig. 37.

#### Comment

These data underestimate the use of FRAX for a number of reasons. As stated above, a session can include more than one calculation and appears to underestimate the number of calculations by approximately 30%. Furthermore, these data only relate to the use of the online FRAX tool. FRAX calculations on bone densitometers are not performed through the web site; in some countries these may account for 25% of calculations or more. In addition, hand-held calculators are used in several countries, including Poland, Belgium, France, Germany, Italy, and Switzerland Poland. FRAX is also available as an application on the iPhone.

These data also underestimate the use of risk assessment in Germany. Fracture risk assessment comprises a component of the German national guidelines but is not FRAX based. Alternative assessment algorithms are also available in the UK, Italy and the Netherlands.

The caveats above indicate that the figures are conservative. Even so, there are reasons to believe that FRAX is underutilised. For example, the use of FRAX in Denmark (319 calculations /million people per year) is very much lower than the number of BMD tests/year (about 67,000 tests /million per year; see *Chapter 4a*).

#### References

216. Kanis JA, Johnell O, Oden A, Johansson H, McCloskey E (2008) FRAX and the assessment of fracture probability in men and women from the UK. Osteoporos Int 19:385–397
217. Kanis JA, On behalf of the World Health Organization Scientific Group (2008) Assessment of Osteoporosis at the Primary Health Care Level. WHO Scientific Group technical report. University of Sheffield, UK. Accessed April 2020 http://www.shef.ac.uk/FRAX/pdfs/WHO_Technical_Report.pdf
218. Kanis JA, Cooper C, Rizzoli R, Reginster JY, On, behalf of the Scientific Advisory Board of the European Society for Clinical and Economic Aspects of Osteoporosis (ESCEO) and the Committees of Scientific Advisors and National Societies of the International Osteoporosis Foundation (IOF) (2019) European guidance for the diagnosis and management of osteoporosis in postmenopausal women. Osteoporos Int 30:3–44
219. Lekamwasam S, Adachi JD, Agnusdei D, Bilezikian J, Boonen S, Borgstrom F, Cooper C, Diez Perez A, Eastell R, Hofbauer LC, Kanis JA, Langdahl BL, Lesnyak O, Lorenc R, McCloskey E, Messina OD, Napoli N, Obermayer-Pietsch B, Ralston SH, Sambrook PN, Silverman S, Sosa M, Stepan J, Suppan G, Wahl DA, Compston JE (2012) A framework for the development of guidelines for the management of glucocorticoid-induced osteoporosis. Osteoporos Int 23:2257–2276
220. McCloskey EV, Johansson H, Harvey NC, Compston J, Kanis JA (2017) Access to fracture risk assessment by FRAX and linked National Osteoporosis Guideline Group (NOGG) guidance in the UK-an analysis of anonymous website activity. Osteoporos Int 28:71–76

### 4c—Treatment uptake and treatment gap

#### Domain

Service uptake-scorecard elements

#### Background and aims

Many studies have demonstrated that a significant proportion of men and women are at high fracture risk do not receive therapy for osteoporosis [221, 222]. The objective in this section is to estimate the proportion of women at high fracture risk that do not receive therapy for osteoporosis in European countries.

#### Methods

The proportion of patients eligible for treatment depends on defining an intervention threshold, i.e. the risk of fracture above which treatment can be recommended. Although national treatment guidelines are available in nearly all EU member states (*Chapters 3e and 3f*), there is no uniform European approach to intervention thresholds across the EU countries. In most countries, however, treatment is recommended in women with a prior fragility fracture. For this reason, intervention thresholds are commonly defined as the 10-year probability of a major fracture that equals or exceeds that of a woman with a prior fragility fracture (see *Chapter 1 g*) [223–226]. For this analysis, the population at risk comprised the number of women age 50 years or older in whom FRAX 10-year probabilities of a major osteoporotic fracture exceeded this threshold.

The number of patients treated in each country was computed from sales data of osteoporosis treatments, provided by IQVIA (formerly Quintiles and IMS Health) for 2019 and expressed as standard units. A standard unit is defined by IQVIA as the number of standard ‘dose’ units sold. The dose units are determined by counting the number of units sold divided by a standard unit factor (the smallest common dose of a product form). Hormone replacement therapy, calcium and vitamin D were not included in the data used for this analysis. It was assumed that 85% of the total drug usage was given to women and 15% to men as estimated from data from the Swedish Prescribed Drug Register. Not all patients adhere to therapy for a full year. Thus, the number of treatment years underestimates the number of individuals actually starting treatment. Data were adjusted by a factor of 23% to account for incomplete adherence as estimated from data from the Swedish Prescribed Drug Register. It is unlikely that 100% of sales are captured in any country but it is difficult to define the magnitude of underestimation. However, IQVIA attempts to correct for under- and over-estimation and for parallel trade.

The number of women potentially treated was subtracted from the number of women exceeding the intervention threshold and the difference expressed as a percentage of the number of women exceeding the intervention threshold (i.e. the proportion of women at high fracture risk who did not receive treatment- the treatment gap). No sales data were available for Cyprus or Malta and these two countries were therefore excluded from analyses.

Estimates for 2019 were compared with data available in 2010 for 27 countries.

#### Results

Table 44 shows that there is a marked variation in the estimated treatment gap between countries being lowest in Ireland (32%) and highest in Bulgaria (86%). Large treatment gaps were identified in countries with populations at both high and low risk of fracture. On average, the treatment gap was 71%, and of the 21 million women that exceed the intervention threshold, 15 million were untreated. These figures are conservative since they do not account for the proportion of low-risk women that have received treatment (see comments, below).

**Table 44:** Number of women eligible for treatment, treated and treatment gap in 2019

Country	Number potentially treated (000)	Number exceeding fracture risk threshold (000)	Difference (000)	Treatment gap (%)
Austria	157	325	168	52
Belgium	150	441	291	66
Bulgaria	35	273	239	87
Croatia	31	169	138	82
Cyprus	-	-	-	-
Czech Republic	74	360	285	79
Denmark	125	218	93	43
Estonia	7	42	35	84
Finland	39	193	154	80
France	550	2 569	2 019	79
Germany	761	3 238	2 477	76
Greece	275	485	211	43
Hungary	125	361	236	65
Ireland	104	153	49	32
Italy	834	2 889	2 055	71
Latvia	16	74	57	78
Lithuania	19	107	88	82
Luxembourg	5	19	14	74
Malta	-	-	-	-
Netherlands	308	696	388	56
Poland	205	1 236	1 031	83
Portugal	118	474	356	75
Romania	130	599	469	78
Slovakia	75	165	90	54
Slovenia	32	74	42	57
Spain	656	1 827	1 171	64
Sweden	128	389	261	67
Switzerland	143	827	684	83
UK	918	2 679	1 761	66
EU27+2	6 017	20 882	14 862	71

**Fig. 38:**
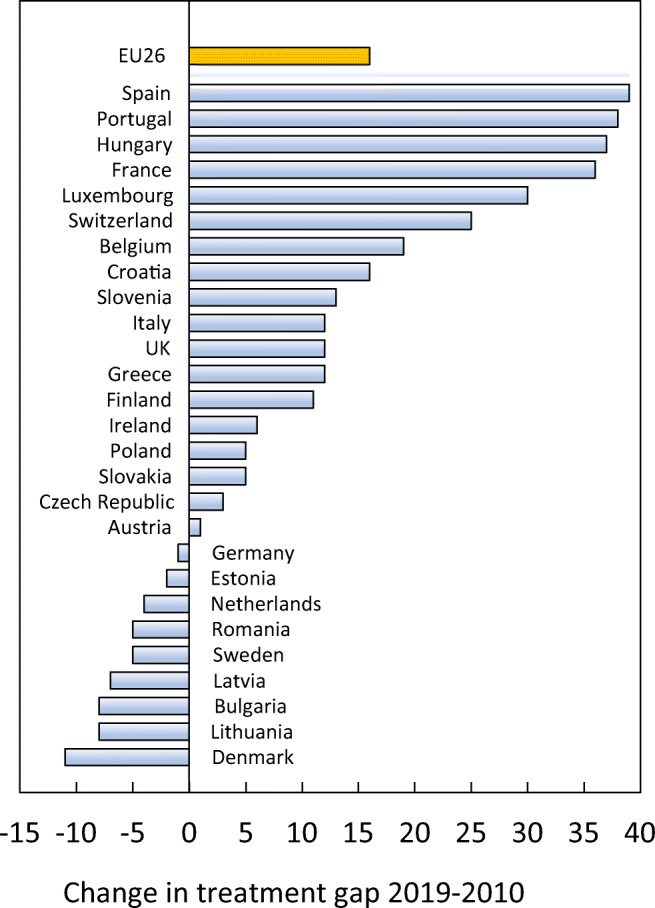
Change in treatment gap between 2010 and 2019

In the majority of counties, the treatment gap for 2019 was greater than that reported in 2010. The treatment gap increased in 18 countries and decreased in 9 countries (Fig. 38). When combining all countries included in the study, the treatment gap increased from 55% in 2010 to 71% in 2019. Overall, 10.6 million women who were eligible for treatment were untreated in 2010. In 2019, this number had risen to 14.0 million.

These data provide two scorecard elements, the first with regard to the current treatment gap and the second to document the change in the treatment gap

#### Score criteria (1)

The criteria for score allocation are divided into approximated tertiles and shown in Table 45.

**Table 45:**
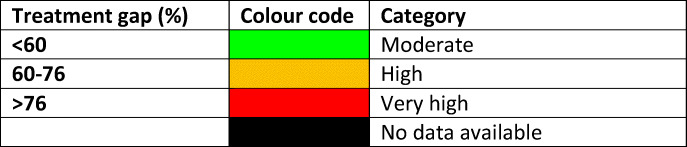
Criteria for allocating scores (1)

#### Score allocation (1)

**Fig. 39:**
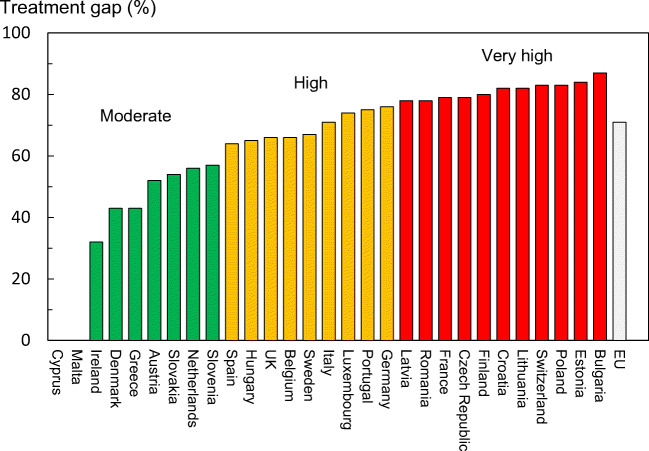
Treatment gap for women in 2019. EU includes all countries from the EU27+2 except Cyprus and Malta

The score allocation and the treatment gap for each country is shown in Fig. 39

#### Score criteria (2)

The criteria for score allocation determined from the change in the treatment gap since 2010 and shown in Table 46.

**Table 46:**
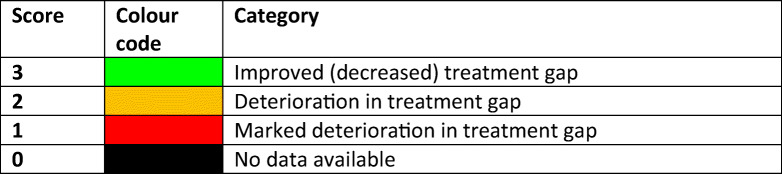
Criteria for allocating scores (2)

#### Score allocation (2)

**Fig. 40:**
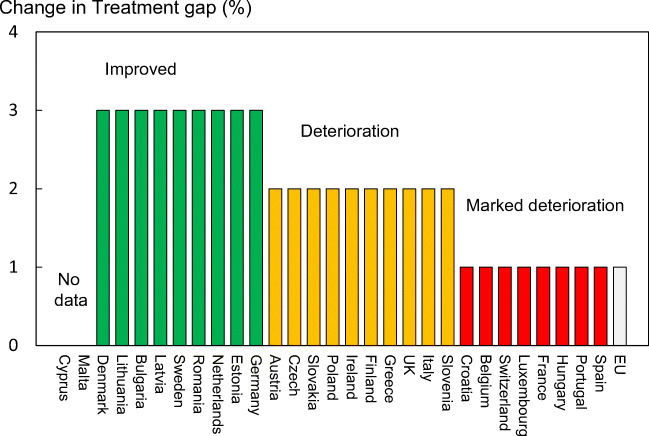
Percent change in treatment gap for women in 2019. EU includes all countries from the EU27+2 except Cyprus and Malta

The score allocation and the treatment gap for each country is shown in Fig. 40.

#### Comments

These data demonstrate that a large number of women at high risk of fractures are not receiving treatment. The estimate is conservative in that it assumes that treatments are targeted only to women at high risk. In 2010, a substantial minority of women at low risk were prescribed treatment in six European countries (13–22%) [227]. Thus, Treatment gaps are likely to be greater than the given estimates. The very large (and increasing) treatment gaps mean that a colour code of green should not be interpreted as an optimum.

A minority of men and women at high fracture risk actually receive treatment in the EU. Moreover, the treatment gap is increasing with time. The under-treatment of osteoporosis globally [228] has led societies such as the International Osteoporosis Foundation and the American Society for Bone and Mineral Research to come together to urgently address this global crisis in the management of osteoporosis [222, 229, 230]. This contrasts with the situation following myocardial infarction, for which condition a significant care gap has been overcome in the past 15 years: 75% of such individuals now receive beta blockers to help prevent recurrent myocardial infarction [231].

Studies to date provide little insight into the causes underlying the substantial and increasing treatment gap. Factors that may play a role include a decline in BMD testing owing to reimbursement issues and lack of intensive detailing by pharmaceutical companies. Others point the finger at the lay press for raising awareness over the last decade of the potential side effects of the bisphosphonates, such as osteonecrosis of the jaw, atypical femoral fractures, and atrial fibrillation [230, 232]. Indeed, many doctors, dentists, and patients are now more frightened of the rare but serious side effects than they are of the disease and the fractures that arise.

Few studies have documented the cost of medication non-adherence. A systematic review estimated the average cost of non-adherence to be € 28.311/patient (2020 prices), substantially more than for cardiovascular disease (€ 7,928), mental health (€ 9,520), diabetes mellitus (€ 5,435) or gastrointestinal disease (€ 20,085) [233]. Quantifying the cost of medication non-adherence may incentivise health policy discussion about the value of medication adherence and promote the adoption of medication adherence intervention programmes.

**Fig. 41:**
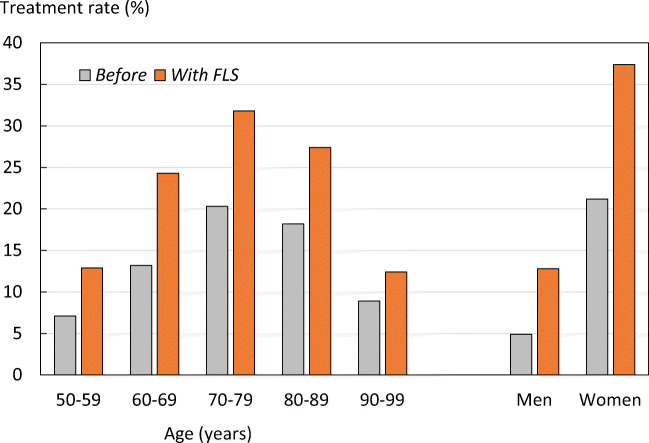
Treatment uptake in the year following a major osteoporotic fracture before and after the institution of FLSs by age and sex

There is now good evidence that treatment uptake is improved by the institution of fracture liaison services (FLS). In a large recent study that avoided referral biases [234], treatment uptake following the FLS increased by 76% within the first year following a major osteoporotic fracture in Swedish women. In men, the uptake was more than doubled [235] (Fig. 41). However, a large treatment gap was still evident in men (87%) and women (63%), particularly at the extremes of age. High treatment uptakes are also reported in a large trial of screening in the elderly female community [236].

#### References

221. Borgstrom F, Karlsson L, Ortsater G, Norton N, Halbout P, Cooper C, Lorentzon M, McCloskey EV, Harvey NC, Javaid MK, Kanis JA (2020) Fragility fractures in Europe: burden, management and opportunities. Arch Osteoporos 15:59.
222. Harvey NC, McCloskey EV, Mitchell PJ, Dawson-Hughes B, Pierroz DD, Reginster JY, Rizzoli R, Cooper C, Kanis JA (2017) Mind the (treatment) gap: a global perspective on current and future strategies for prevention of fragility fractures. Osteoporos Int 28:1507–1529
223. Compston JCA, Cooper C, Francis R, Kanis JA, Marsh D, McCloskey EV, Reid DM, Selby P, Wilkins M (2009) Guidelines for the diagnosis and management of osteoporosis in postmenopausal women and men from the age of 50 years in the UK. Mauritas 62:105–108
224. Lekamwasam SAJ, Agnusdei D, Bilezikian J, Boonen S, Borgström F, Cooper C, Diez Perez A, Eastell R, Hofbauer LC, Kanis JA, Langdahl BL, Lesnyak O, Lorenc R, McCloskey E, Messina OD, Napoli N, Obermayer-Pietsch B, Ralston SH, Sambrook PN, Silverman S, Sosa M, Stepan J, Suppan G, Wahl DA, Compston JE (2012) A framework for the development of guidelines for the management of glucocorticoid-induced osteoporosis. Osteoporosis International 23:2257–2276
225. Kanis JA, Harvey NC, Cooper C, Johansson H, Oden A, McCloskey EV (2016) A systematic review of intervention thresholds based on FRAX : A report prepared for the National Osteoporosis Guideline Group and the International Osteoporosis Foundation. Arch Osteoporos 11:25
226. Kanis JA, Cooper C, Rizzoli R, Reginster6 J-Y, On, behalf of the Scientific Advisory Board of the European Society for Clinical and Economic Aspects of Osteoporosis (ESCEO) and the Committees of Scientific Advisors and National Societies of the International Osteoporosis Foundation (IOF) (2020) European guidance for the diagnosis and management of osteoporosis in postmenopausal women. Osteoporosis International 30:3–44
227. Kanis JA, Borgstrom F, Compston J, Dreinhofer K, Nolte E, Jonsson L, Lems WF, McCloskey EV, Rizzoli R, Stenmark J (2013) SCOPE: a scorecard for osteoporosis in Europe. Arch Osteoporos 8:144
228. Kanis JA, Svedbom A, Harvey N, McCloskey EV (2014) The osteoporosis treatment gap. J Bone Miner Res 29:1926–1928
229. Conley RB, Adib G, Adler RA, Åkesson KE, Alexander IM, Amenta KC, Blank RD, Brox WT, Carmody EE, Chapman-Novakofski K, Clarke BL, Cody KM, Cooper C, Crandall CJ, Dirschl DR, Eagen TJ, Elderkin AL, Fujita M, Greenspan SL, Halbout P, Hochberg MC, Javaid M, Jeray KJ, Kearns AE, King T, Koinis TF, Koontz JS, Kužma M, Lindsey C, Lorentzon M, Lyritis GP, Michaud LB, Miciano A, Morin SN, Mujahid N, Napoli N, Olenginski TP, Puzas JE, Rizou S, Rosen CJ, Saag K, Thompson E, Tosi LL, Tracer H, Khosla S, Kiel DP (2020) Secondary Fracture Prevention: Consensus Clinical Recommendations from a Multistakeholder Coalition. J Bone Miner Res 35:36–52
230. Khosla S, Cauley JA, Compston J, Kiel DP, Rosen C, Saag KG, Shane E (2017) Addressing the Crisis in the Treatment of Osteoporosis: A Path Forward. J Bone Miner Res 32:424–430
231. Austin PC, Tu JV, Ko DT, Alter DA (2008) Factors associated with the use of evidence-based therapies after discharge among elderly patients with myocardial infarction. Cmaj 179:901–908
232. Solomon DH, Johnston SS, Boytsov NN, McMorrow D, Lane JM, Krohn KD (2014) Osteoporosis medication use after hip fracture in U.S. patients between 2002 and 2011. J Bone Miner Res 29:1929–1937
233. Cutler RL, Fernandez-Llimos F, Frommer M, Benrimoj C, Garcia-Cardenas V (2018) Economic impact of medication nonadherence by disease groups: a systematic review. BMJ Open 8: e016982
234. Axelsson KF, Johansson H, Lundh D, Möller M, Lorentzon M (2020) Association Between Recurrent Fracture Risk and Implementation of Fracture Liaison Services in Four Swedish Hospitals: A Cohort Study. J Bone Miner Res 35:1216–1223
235. Axelsson KFL, M. (2020) Personal communication. Received by Kanis JA.
236. Parsons CM, Harvey N, Shepstone L, Kanis JA, Lenaghan E, Clarke S, Fordham R, Gittoes N, Harvey I, Holland R, Redmond NM, Howe A, Marshall T, Peters TJ, Torgerson D, O'Neill TW, McCloskey E, Cooper C (2020) Systematic screening using FRAX(®) leads to increased use of, and adherence to, anti-osteoporosismedications: an analysis of theUKSCOOP trial. Osteoporos Int 31:67–75

### 4d—Treatment gap and treatment needed

#### Domain

Service uptake—Background information

#### Background and aims

Patients who sustain a prior fragility fracture are at high risk of a future fracture. The risk is increased approximately two-fold [237] and is largely independent of BMD [238]. The risk is sufficiently high that most treatment guidelines in the EU and elsewhere recommend that postmenopausal women with a prior fragility fracture should be offered treatment. However, the majority of such patients are untreated so that the prevalence of a prior fracture in the community provides an index of opportunity lost. This may be set against the treatment gap to provide an index of the relationship between service provision and service need. The aim of this element was to provide an index of the prevalence of a prior fracture in the EU member countries in relation to the treatment gap.

#### Methods

For the purposes of this report, a prior fracture was defined as a hip or clinical vertebral fracture in an individual who was alive in 2019, who had incurred that fracture after the age of 50 years and prior to 2019. The unit was the individual, so that multiple fractures at the same site in an individual were only counted once. A micro-simulation model, programmed in TreeAge, was used to simulate the prevalence of prior hip and vertebral fractures from incidence data [239].

Note that the prevalence of a hip or clinical vertebral fracture will underestimate the prevalence of previous fragility fracture at other sites. More complete information on prior fractures was available for six member states (France, Germany, Italy, Spain, Sweden and the UK) using a different modelling approach [240]. For the treatment gap, data from *Chapter 4c* were used.

#### Results

In 2019, approximately 6.8 million men and women in the EU27+2 countries had sustained a prior hip or clinical spine fracture before 2019 (Table 47). Overall, 1.5% of the population age 50 years or more had a prior hip fracture and 1.7% a prior clinical spine fracture.

**Table 47:** Estimated number and percentage of men and women aged above 50 years with a prior hip or vertebral fracture by country in 2019

Country	Hip fracture		Vertebral fracture		A + B (%)
	Number	% Population (A)	Number	% Population (B)	
Austria	85,557	2.3	96,403	2.6	4.9
Belgium	79,976	1.8	88,082	1.9	3.7
Bulgaria	33,211	1.1	41,111	1.4	2.6
Croatia	26,607	1.5	31,454	1.8	3.3
Cyprus	4,593	1.2	5,598	1.5	2.6
Czech Republic	57,421	1.4	70,624	1.7	3.1
Denmark	55,373	2.4	65,777	2.8	5.2
Estonia	5,668	1.1	6,800	1.3	2.4
Finland	34,368	1.5	39,894	1.7	3.2
France	332,839	1.3	340,740	1.3	2.6
Germany	606,853	1.6	670,385	1.8	3.4
Greece	79,807	1.8	88,952	2.0	3.7
Hungary	53,985	1.4	66,593	1.8	3.2
Ireland	21,825	1.4	27,003	1.7	3.1
Italy	483,093	1.7	516,946	1.9	3.6
Latvia	12,348	1.6	14,387	1.8	3.4
Lithuania	16,555	1.4	19,628	1.7	3.1
Luxembourg	2,896	1.3	3,346	1.5	2.9
Malta	2,200	1.2	2,714	1.5	2.8
Netherlands	72,719	1.0	85,471	1.2	2.2
Poland	142,035	1.0	175,697	1.2	2.2
Portugal	56,832	1.3	62,486	1.4	2.7
Romania	101,466	1.3	124,460	1.6	3.0
Slovakia	23,636	1.2	30,212	1.5	2.7
Slovenia	12,046	1.4	14,335	1.6	3.0
Spain	258,560	1.3	256,549	1.3	2.7
Sweden	94,818	2.4	108,202	2.8	5.2
Switzerland	62,528	1.8	70,914	2.0	3.8
UK	400,366	1.6	430,254	1.7	3.2
EU27+2	3,220,181	1.5	3,555,016	1.7	3.2

**Fig. 42:**
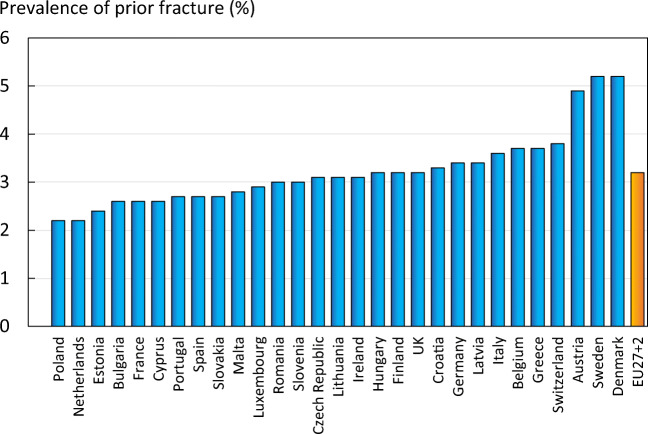
The proportion (%) of the population aged 50 years or more with a prior hip or vertebral fracture in 2019

The ranked prevalences by country are shown in Fig. 42. As would be expected, there was a close relationship between fracture risk (see *Chapter 1d*) and the proportion of the population with a prior hip or clinical vertebral fracture.

The prevalence of prior vertebral and hip fracture (Fig. 42) was compared to the prevalence estimates for all prior fractures in six of the EU member-states (Table 48). The prevalence of a prior fragility fracture ranged from 2.2% in Poland and the Netherlands to 5.2% in Sweden and Denmark. The estimation of prior vertebral + prior hip fracture, shown in, appears to capture approximately 26% of prior fractures. This suggests that the prevalence of a prior clinical spine or hip fracture is a reasonable surrogate for the service needs of each member state.

**Table 48:** The prevalence (%) of a prior fracture at sites associated with osteoporosis in men and women aged 50 years or more compared with the prevalence of a prior vertebral or hip fracture as given in Table 47. The last column shows the proportion (%) of prior fractures accounted for by hip or clinical vertebral fracture [239]

Country	Author	Prevalence (%)		EU/ Comparator (%)
		Comparator	EU27+2	
France	Cawston 2012 [241]	11.2	2.6	23
Germany	Gauthier 2012 [242]	14.1	3.4	24
Italy	Piscitelli 2012 [243]	16.2	3.6	22
Spain	Gauthier 2012 [244]	8.9	2.7	30
Sweden	Gauthier 2011 [240]	22.6	5.2	23
UK	Gauthier 2011[245]	10.3	3.2	31
Average (weighted)				26

**Fig. 43:**
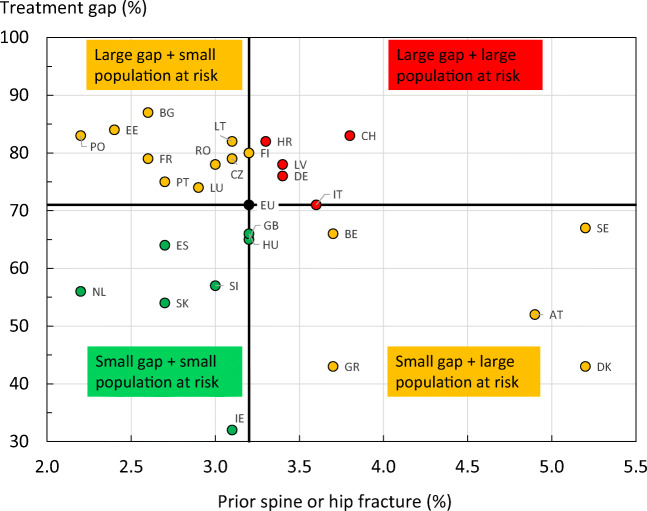
The relationship between the prevalence of a prior spine or hip fracture (service need) and the treatment gap (service provision) in the EU27 countries. The horizontal and vertical lines intersect at the EU average (weighted for population size. Country codes (ISO 3166–1 alpha-2); AT Austria; BE Belgium; BG Bulgaria; HR Croatia; CY Cyprus; CZ Czech Republic; DE Germany; DK Denmark; EE Estonia; ES Spain; FI Finland; FR France; GB UK; GR Greece; HU Hungary; IE Ireland; IT Italy; LT Lithuania; LU Luxembourg; LV Latvia; MT Malta; NL Netherlands; PL Poland; PT Portugal; RO Romania; SE Sweden; SI Slovenia; SK Slovakia; CH Switzerland

The relationship between this service need and the treatment gap is shown in Fig. 43 for each of the EU27+2, as well as all of them combined. The top right quadrant can be considered to represent countries of high need but poor provision. These included Germany, Finland, Croatia, Latvia and Switzerland. The other extreme (lower left quadrant) represents countries of lower need but better provision. These included Spain, Ireland, Slovakia, Slovenia and the Netherlands.

#### Score allocation

Supplementary information, no score allocation

#### Comment

There is a wide variation in both (hip and spine) fractures and treatment gap between countries but no significant correlation between the two variables. This is of particular concern in countries with a high fracture burden and a high treatment gap.

#### References

237. Klotzbuecher CM, Ross PD, Landsman PB, Abbott TA 3rd, Berger M (2000) Patients with prior fractures have an increased risk of future fractures: a summary of the literature and statistical synthesis. J Bone Miner Res 15:721–739
238. Kanis JA, Johnell O, De Laet C, Johansson H, Oden A, Delmas P, Eisman J, Fujiwara S, Garnero P, Kroger H, McCloskey EV, Mellstrom D, Melton LJ, Pols H, Reeve J, Silman A, Tenenhouse A (2004) A meta-analysis of previous fracture and subsequent fracture risk. Bone 35:375–382
239. Hernlund E, Svedbom A, Ivergard M, Compston J, Cooper C, Stenmark J, McCloskey EV, Jonsson B, Kanis JA (2013) Osteoporosis in the European Union: medical management, epidemiology and economic burden. A report prepared in collaboration with the International Osteoporosis Foundation (IOF) and the European Federation of Pharmaceutical Industry Associations (EFPIA). Arch Osteoporos 8:136
240. Gauthier A, Kanis JA, Martin M, Compston J, Borgstrom F, Cooper C, McCloskey E (2011) Development and validation of a disease model for postmenopausal osteoporosis. Osteoporos Int 22:771–780
241. Cawston H, Maravic M, Fardellone P, Gauthier A, Kanis JA, Compston J, Borgstrom F, Cooper C, McCloskey E (2012) Epidemiological burden of postmenopausal osteoporosis in France from 2010 to 2020: estimations from a disease model. Arch Osteoporos 7:237–246
242. Gauthier A, Kanis JA, Jiang Y, Dreinhofer K, Martin M, Compston J, Borgstrom F, Cooper C, McCloskey E (2012) Burden of postmenopausal osteoporosis in Germany: estimations from a disease model. Arch Osteoporos 7:209–218
243. Piscitelli P, Brandi M, Cawston H, Gauthier A, Kanis JA, Compston J, Borgstrom F, Cooper C, McCloskey E (2014) Epidemiological burden of postmenopausal osteoporosis in Italy from 2010 to 2020: estimations from a disease model. Calcif Tissue Int 95:419–427
244. Gauthier A, Kanis JA, Jiang Y, Cannata J, Compston J, Borgstrom F, Cooper C, McCloskey E (2012) Burden of post-menopausal osteoporosis in Spain: Estimations from a disease model. Unpublished
245. Gauthier A, Kanis JA, Jiang Y, Martin M, Compston JE, Borgstrom F, Cooper C, McCloskey EV (2011) Epidemiological burden of postmenopausal osteoporosis in the UK from 2010 to 2021: estimations from a disease model. Arch Osteoporos 6:179–188

### 4e – Waiting time for hip surgery

#### Domain

Service uptake—scorecard element

#### Background and aims

About 5% of people with a hip fracture die within 1 month and about one quarter within 12 months. Most deaths are due to associated conditions and not to the fracture itself [246], reflecting the high prevalence of comorbidity. In the EU27+2, there were estimated to be 248,487 deaths in 2019 that were causally related to the fracture event (*Chapter 1e*). Approximately 43% of fracture-related deaths in women were due to hip fractures, 54% to clinical vertebral and 3% to other fractures. Corresponding proportions for men were 34, 65 and 1%, respectively.

A determinant of peri-operative morbidity and mortality is the time a patient takes to get to surgery which, in turn, is an early marker of a patient’s progress following a hip fracture. Early surgery (<48 h) is associated with a statistically and clinically significant reduction in mortality at 1 year and an increase in the proportion of patients returning to their original residence [247].

The aim of this scorecard element was to determine average waiting times for hip surgery in the EU member states.

#### Methods

Data were acquired through an IOF questionnaire sent to the European National Societies undertaken between March and May 2020. Respondents were asked to provide information on the average waiting time for hip surgery after hip fracture. Countries were categorised according to average waiting times between hospital admission and surgical intervention. An additional indicator of management that was sought was the proportion of hip fracture cases that were managed surgically.

#### Results

Waiting times between admission to hospital and surgical intervention were on average 1 day or less in 11 countries (7 countries in 2010) , 1–2 days in 10 countries (13 countries in 2010) and greater than 2 days in 5 countries (6 countries in 2010). The five countries with the longest average waiting time for hip surgery were Portugal, Spain, Cyprus, Greece and Italy (Table 49). Information was not recorded for Malta.

**Table 49:** Average waiting times between hospital admission and surgical intervention and the proportion of hip fracture cases managed surgically

Country	Waiting time (days) 2010	Waiting time (days) 2019	Score 2019	Surgical management (%) 2019
Austria	1-2	<1	3	>90
Belgium	<1	1-2	2	>90
Bulgaria	1-2	<1	3	75-90
Croatia	nr	<1	3	>90
Cyprus	2-3	2-3	1	75-90
Czech Republic	1-2	1-2	2	67.2
Denmark	1-2	1-2	2	>90
Estonia	<1	nr	nr	nr
Finland	1-2	1-2	2	>90
France	1-2	1-2	2	>90
Germany	<1	<1	3	>90
Greece	2-3	2-3	1	>90
Hungary	<1	<1	3	75-90
Ireland	2-3	1-2	2	>90
Italy	2-3	2-3	1	>90
Latvia	<1	<1	3	>90
Lithuania	<1	<1	3	>90
Luxembourg	1-2	nr	nr	nr
Malta	nr	1-2	nr	nr
Netherlands	1-2	<1	3	>90
Poland	1-2	<1	3	>90
Portugal	2-3	>3	1	>90
Romania	1-2	<1	3	nr
Slovakia	1-2	1-2	2	>90
Slovenia	1-2	1-2	2	>90
Spain	2-3	>3	1	75-90
Sweden	<1	<1	3	>90
Switzerland	N/A	1-2	2	>90
UK	1-2	1-2	2	>90

*nr,* not recorded

For comparison, the 2010 values are provided in Table 49. Since 2010, six countries have increased their score (Ireland, Austria, Bulgaria, Netherlands, Poland and Romania), while one country has worsened (Belgium). These changes indicate an overall improvement in waiting times for the EU.

More than 90% of hip fracture cases received surgery in the majority of countries. Exceptions included Bulgaria, Cyprus, Czech Republic, Hungary and Spain, where 65–90% of cases received a surgical intervention.

#### Score criteria

Uptake was categorised by average waiting time for hip surgery (Table 50)

**Table 50:**
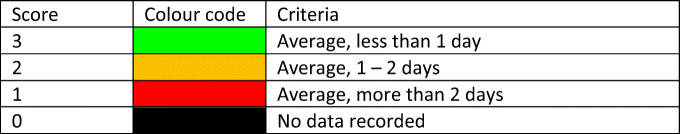
Criteria for allocating scores

#### Score allocation

**Fig. 44:**
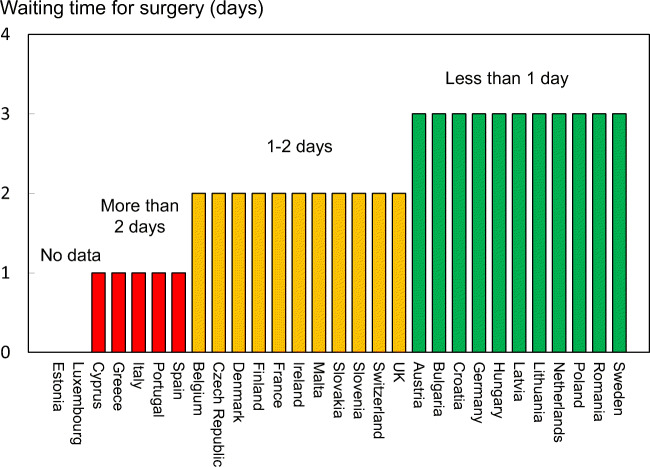
Countries categorised by the average waiting time for surgical intervention for hip fracture

Score results provided in Table 49 are colour coded and presented in Fig. 44.

#### Comment

Note that average waiting times give no index of the dispersion around the mean.

#### References

246. Kanis JA, Oden A, Johnell O, De Laet C, Jonsson B, Oglesby AK (2003) The components of excess mortality after hip fracture. Bone 32:468–473
247. National Clinical Guideline Centre (2011) The Management of Hip Fracture in Adults. London. Accessed Jan 2013 https://www.nice.org.uk/guidance/cg124/evidence/full-guideline-183081997

## Chapter 5: Scores and scorecard

### The scorecard

The second edition of the Scorecard for Osteoporosis in Europe (SCOPE 2021) allows health and policy professionals to assess key indicators on the healthcare provision for osteoporosis within countries and between counties within the EU 27+2.

### Domain Summaries

**Fig. 45:**
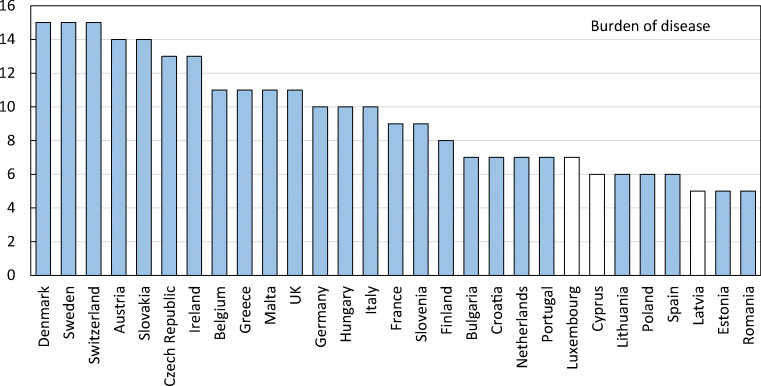
Total scores by country for metrics related to Burden of disease. The score for each of the 5 metrics is given in Chapter 1 and subtracted from 15 (the highest possible score). An unfilled bar denotes that there was one or more missing metric which might affect the burden score

In total, 16 scored metrics have been measured within four domains, Burden of disease, Policy framework, Service provision and Service uptake. The Burden of disease in the EU27+2 countries is summarised in Fig. 45, in rank order. The higher the score, the greater the burden. Denmark, Sweden and Switzerland had the highest score. The place of Luxembourg, Cyprus and Latvia are uncertain since there were gaps in the information base.

**Fig. 46:**
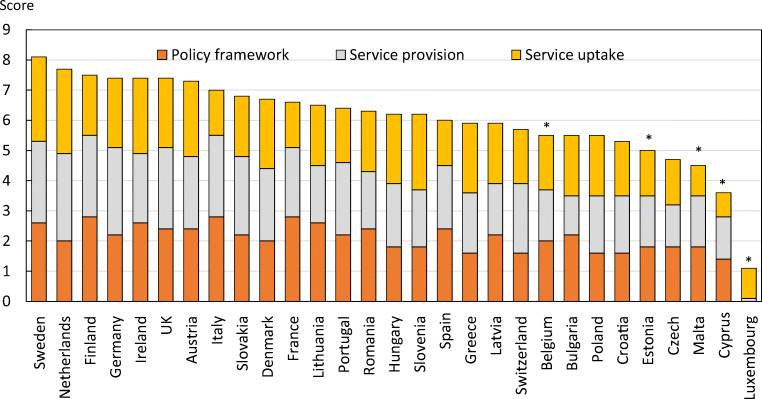
Scores by country for metrics related to policy framework, service provision and service uptake. The mean score for each of the 3 domains is given. An asterisk denotes that there was one or more missing metric which decreases the overall score

The domains of Policy framework, Service provision and Service uptake can be considered aspects of health care delivery. Their combined contribution is shown in Fig. 46. Sweden and Netherlands ranked first and second and, of those countries with complete information, the Czech Republic and Estonia ranked last.

**Fig. 47:**
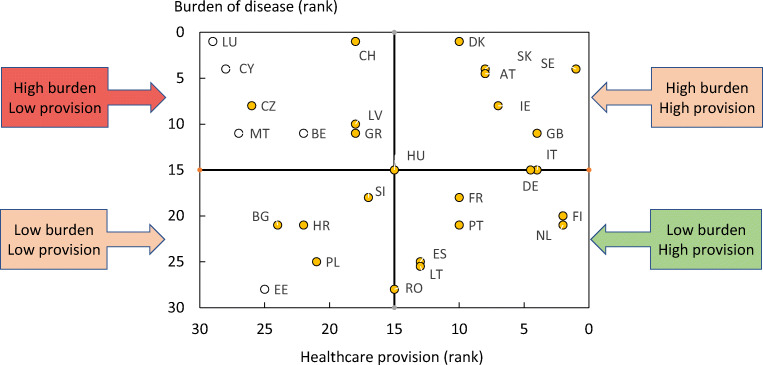
The relationship between the Burden of disease and the healthcare provision (Policy framework, Service provision and Service uptake) in the EU27+2 countries. The horizontal and vertical lines intersect at the median rankings. Open circles denote countries with missing information either in health care provision or burden of disease. Country codes (ISO 3166–1 alpha-2); AT Austria; BE Belgium; BG Bulgaria; HR Croatia; CY Cyprus; CZ Czech Republic; DE Germany; DK Denmark; EE Estonia; ES Spain; FI Finland; FR France; GB UK; GR Greece; HU Hungary; IE Ireland; IT Italy; LT Lithuania; LU Luxembourg; LV Latvia; MT Malta; NL Netherlands; PL Poland; PT Portugal; RO Romania; SE Sweden; SI Slovenia; SK Slovakia; CH Switzerland

The highest health care provision did not necessarily match the burden of disease. Figure 47 shows the rankings for healthcare provision (Policy framework, Service provision and Service uptake combined) as shown in Figure 2 and that for burden of disease. High health care provision was commensurate with high burden of disease (e.g. Austria, Denmark, Ireland, Slovakia and the UK). Conversely low burden of disease was associated with low health care provision (e.g. Bulgaria, Estonia, Hungary, Poland and Slovenia). The most advantageous scenarios (low burden of disease but high healthcare provision) were seen in Finland, France, Lithuania, Netherlands, Portugal and Spain. The least favourable scenarios (high burden, low provision) were noted for Belgium, Cyprus, Czech Republic, Greece, Latvia, Luxembourg and Malta.

### Time trends

**Fig. 48:**
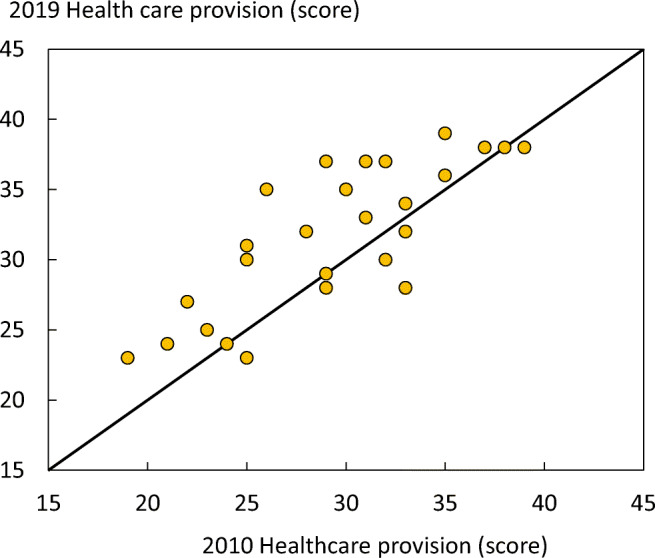
Correlation between the total score for health care provision in 2019 and 2010. The solid line indicates the line of identity. Luxembourg is not included because of the large amount of missing data

The first SCOPE was undertaken in 2010, almost 10 years previously. Fifteen of the 16 score card metrics were used in the two surveys. In so far as was possible, the same or similar questions were used. This provided the opportunity to compare performance over time in those 27 countries that were assessed on both occasions (this excluded Croatia and Switzerland). As might be expected, there was a close correlation between the cumulative score in 2019 with that observed in 2010 (*R* = 0.80) (Fig. 48). It is of note that an improved score in 2019 was seen in the majority of countries. Numerical values for the scores are given in Table 51. Slovakia, Ireland, Italy, Lithuania, France, Germany, Poland, Romania, Finland, Malta, Spain, Cyprus, Bulgaria, Austria and Denmark improved their score over 9 years. Belgium, Czech Republic and Slovenia had a worse score in 2019 than in 2010.

**Table 51:** Scores for health care provision in 2019, 2010 and the difference based on 15 common metrics. Ticks denote countries where there was one or more missing metric which might obfuscate the change in the overall score. Luxembourg is not included because of the large amount of missing data

Country	Score 2019	Score 2010	2019–2010	Incomplete data	Category
Slovakia	35	26	9		Marked improvement
Ireland	37	29	8		
Italy	37	31	6		
Lithuania	31	25	6		
France	35	30	5		Much improved
Germany	37	32	5		
Poland	27	22	5		
Romania	30	25	5		
Finland	39	35	4		
Malta	23	19	4	√	
Spain	32	28	4		
Cyprus	24	21	3	√	Improved
Bulgaria	25	23	2	√	
Denmark	33	31	2		
Austria	37	35	1		
Netherlands	38	37	1		Unchanged or marginal change
Portugal	34	33	1		
Estonia	24	24	0	√	
Greece	29	29	0		
UK	38	38	0		
Hungary	32	33	-1		
Latvia	28	29	-1	√	
Sweden	38	39	-1		
Czech Republic	23	25	-2		Worse score
Slovenia	30	32	-2		
Belgium	28	33	-5

**The Scorecard:**
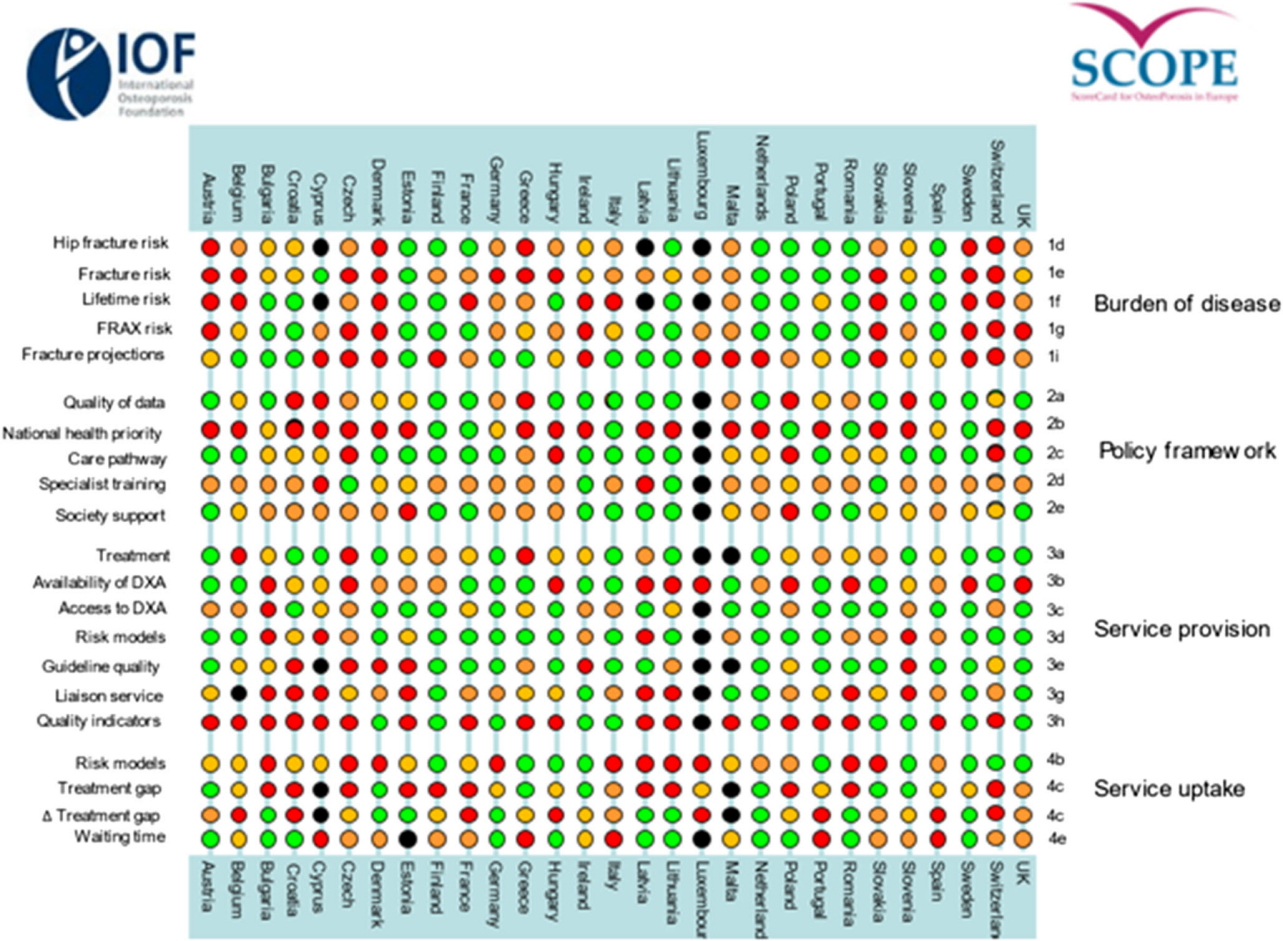
The scorecard and key are provided below

**The Scorecard:**
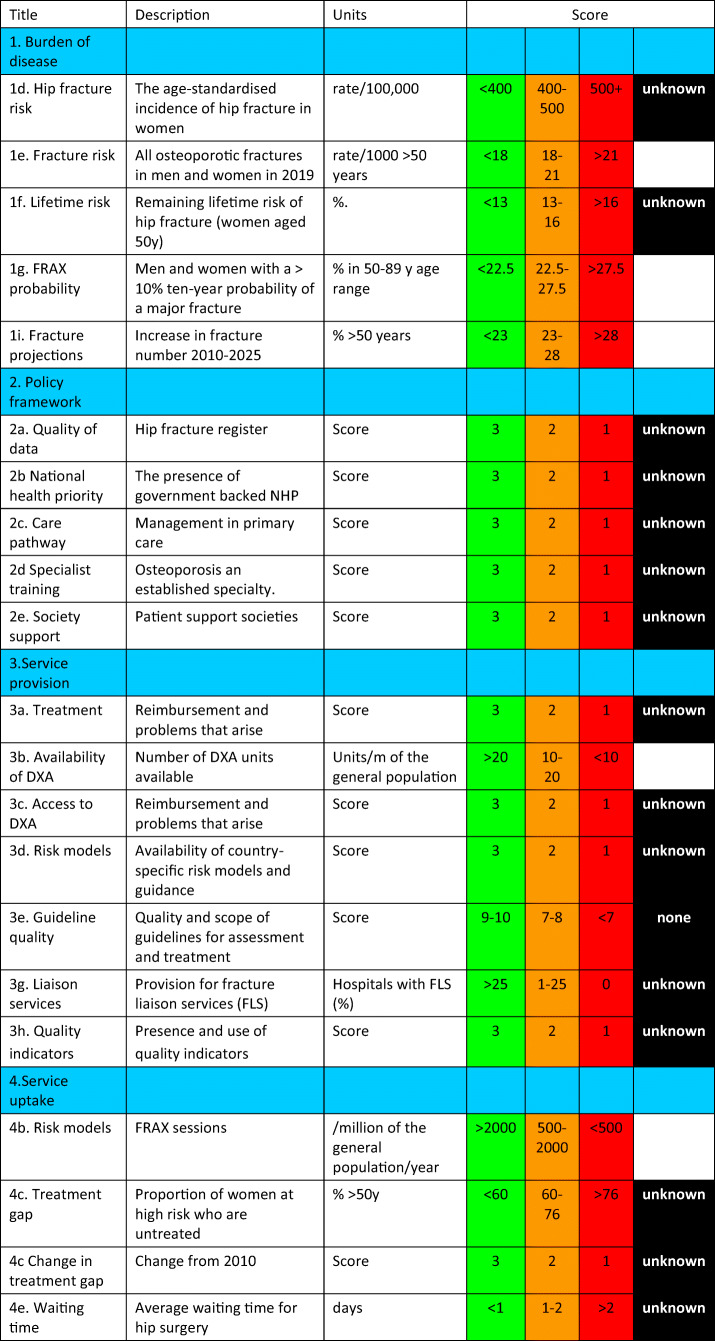
The scorecard and key are provided below

The scorecard is not intended as a prescriptive template. Thus, it does not set performance targets but may serve as a guide to the performance targets at which to aim in order to deliver the outcomes required

## Footnotes

1 AGREE criteria Systematic search. How thorough was the evidence base? Were the guidelines based on a systematic literature review conducted at the time of the guideline development (or on a previously conducted review that was updated). Recommendations: Were recommendations graded (e.g. A, B, C) according to the levels of evidence provided by the systematic review. Stakeholder involvement: Was there involvement from patient organisations, primary care physicians, national/EU societies in the consultation process for the guidelines? External review: Were the guidelines reviewed by independent experts? i.e. have they undergone a rigorous external review in addition to consultation. Procedure for update: Were the guidelines updated as and when necessary or was there explicit mention of a provision to update the guidelines in the future? Economic analysis: Were the recommendations underpinned by an economic analysis? Editorial independence: Did the guidelines explicitly state that there was editorial independence of the writing group from any funding body?

## Abbreviations and glossary

AGREE: Appraisal of Guidelines for Research & Evaluation. Quality score for guidelines.
BMD: Bone mineral density, usually measured as the amount of calcium in bone per unit area.
CtF: Capture the Fracture® (CtF), a global initiative of IOF to facilitate the implementation of coordinated, multidisciplinary models of care for secondary fracture prevention.
DALY: Disability-adjusted life year, a product of years of life lost and the remaining years of life disabled (i.e., disutility).
Direct costs: Used in health technology assessment to describe direct healthcare costs (e.g., hospital admissions, medical examinations, drug therapy, etc.). Indirect costs include losses in productivity resulting from absence to work and intangible costs include pain and suffering, poor quality of life.
DXA: Dual-energy x-ray absorptiometry, a method for measuring BMD
EU 27: The 27 member states of the European Union
EU 27+2: The 27 member states of the European Union and Switzerland and the United Kingdom
FLS: Fracture liaison services (osteoporosis coordinator programmes or care manager programs), provide a system for the routine assessment and management of individuals who have sustained a low trauma fracture
FRAX: Fracture risk assessment tool developed by the WHO Collaborating Centre, University of Sheffield Medical School, UK. FRAX calculates the 10- year probability of a major osteoporotic fracture and hip fracture in individuals from clinical risk factors and BMD.
GDP: Gross domestic product, the total value of goods produced, and services provided in a country in one year
GIOP: Glucocorticoid-induced osteoporosis
GP: General practitioner (primary care physician)
GPRD: General Practice Research Database
Incidence: The frequency of an event, usually expressed as a yearly rate e.g., 10 per 1000 of the population/year.
IOF: International Osteoporosis Foundation
NHANES: National health and nutrition examination survey
NHP: National Health Priority
NICE: National Institute of Health and Clinical Excellence
NOGG: National Osteoporosis Guideline Group
PMW: Postmenopausal women
Prevalence: The number of cases of disease (e.g., osteoporosis) for a given area or at a given time
Probability: The likelihood of an event e.g., fracture. Fracture probability depends on two hazards – the incidence of fracture and the incidence of death
QALY: Quality adjusted life year. The QALY is a multi-dimensional outcome measure that incorporates both the quality (health related) and quantity (length) of life. The value of a QALY was set at value of 2 × GDP per capita
QCT: Quantitative computed tomography
QoL: Quality of life
QUS: Quantitative ultrasound
SCOPE: Scorecard for osteoporosis in Europe
SD: Standard deviation
T-score: Describes the number of standard deviations (SD) by which the BMD in an individual differs from the mean value expected in young healthy women. The operational definition of osteoporosis is defined as a value for BMD 2.5 SD or more below the young female adult mean (T-score less than or equal to –2.5 SD).
TBS: Trabecular bone score
VFA: Vertebral fracture assessment
WHO: World Health Organization
WTP: Willingness to pay, used in Health Technology assessment to describe the value that society or a health care payer is prepared to pay to gain a QALY. The value of a QALY was set at value of 2 × GDP per capita.
YLD: Years of life with disability

